# Chiral Phosphoric Acid Promoted Chiral 1H NMR Analysis of Atropisomeric Quinolines

**DOI:** 10.3389/fchem.2021.672704

**Published:** 2021-06-10

**Authors:** Junlin Wan, Jun Jiang, Juan Li

**Affiliations:** College of Chemistry and Materials Engineering, Wenzhou University, Wenzhou, China

**Keywords:** chiral recognition, 1H NMR analysis, quinolines, chiral phosphoric acid, chiral shift reagents

## Abstract

An efficient enantioselective NMR analysis of atropisomeric quinolines in the promotion of chiral phosphoric acid is described, in which a variety of racemic 4-aryl quinolines were well-recognized with up to 0.17 ppm ΔΔδ value. Additionally, the optical purities of different nonracemic substrates could be evaluated fast via NMR analysis with high accuracy.

## Introduction

Axial chirality is one of the important types of molecular asymmetry created from restriction of carbon–carbon or carbon–nitrogen single-bond rotation. Since Christie and Kenner reported the first detection of atropisomerism in 1922 (Christie and Kenner, 1922), axial chirality was found in a lot of natural products and pharmaceutical compounds as exemplified by michellamines (Manfredi et al., 1991; Bringmann et al., 1993) and vancomycin(Nicolaou et al., 1999). Besides, many chiral ligands and catalysts, such as BINOL, BINAP, and phosphoric acids, have been developed based on axially chiral biaryl scaffolds(Miyashita et al., 1980; Akutagawa, 1995; Kumobayashi et al., 2001; Brunel, 2005; Brunel, 2007; Genet et al., 2014). It is well-known that the enantiopurities of chiral ligands and catalysts are critical to their enantiocontrol, and atropisomers of bioactive molecules always exhibit different pharmacodynamic and pharmacokinetic behavior both *in vivo* and in vitro (Eichelbaum and Gross, 1996; Clayden et al., 2009). Thus, the development of efficient methods to recognize and determine atropisomeric compounds becomes an interesting target and is always in high demand. As key analysis methods, GC (Schurig and Nowotny, 1990), IR (Reetz et al., 1998), HPLC (Han, 1997), circular dichroism (Ding et al., 1999; Nieto et al., 2008; Ghosn and Wolf, 2009; Nieto et al., 2010), fluorescence spectroscopy (James et al., 1995; Mei and Wolf, 2004; Pu, 2004; Zhao et al., 2004; Tumambac and Wolf, 2005; Liu et al., 2009), electrophoresis technologies (Reetz et al., 2000), and NMR spectroscopy have been efficiently employed in chiral determinations. Among these classic technologies, NMR analysis affords an ideal platform to explore efficient chiral analysis strategies because of its mild condition, easy operation, fast evaluation, high sample tolerance, etc. Over the past few decades, a lot of chiral shift reagents (CSRs) (Frazer et al., 1971; Goering et al., 1971; Yeh et al., 1986; Ghosh et al., 2004; Yang et al., 2005; Mori et al., 2013) or chiral solvating reagents (CSAs) (Pirkle, 1966; Lancelot et al., 1969; Parker, 1991; Wenzel and Wilcox, 2003; Seco et al., 2004; Lovely and Wenzel, 2006; Ema et al., 2007; Wenzel, 2007; Iwaniuk and Wolf, 2010; Moon et al., 2010; Gualandi et al., 2011; Pham and Wenzel, 2011; Quinn et al., 2011; Wenzel and Chisholm, 2011; Ma et al., 2012; Labuta et al., 2013; Zhou et al., 2015; Bian et al., 2016a; Akdeniz et al., 2016; Bian et al., 2016b; Huang et al., 2016) were successfully designed and employed in chiral NMR analysis. Encouraged by these achievements and our continuous efforts to study chiral interactions, we were particularly interested in exploring a novel NMR-based chiral analysis method for our synthetic targets: In 2017, we reported an enantioselective NMR analysis of indoloquinazoline alkaloid–type tertiary alcohols with chiral phosphoric acid (CPA) (Akiyama et al., 2006; Akiyama, 2007; Akiyama and Mori, 2015) promotion, in which a fast reaction condition optimization of amino acid metal salt–catalyzed asymmetric aldol reaction was also achieved (Liu et al., 2017); besides, a variety of racemic 4-aryl quinazolinones, such as afloqualone and IC-87114, were also well-recognized, and the optical purities of different nonracemic substrates could be evaluated fast with high accuracy (Wu et al., 2018). Encouraged by these results and our recent research on the catalytic asymmetric construction of atropisomeric quinolines, we wish to report an efficient chiral recognition of quinoline atropisomers by chiral phosphoric acid: In the presence of 1 equivalent of α-naphthyl phosphoric acid, a variety of racemic quinolines were well-recognized with up to 0.17 ppm ΔΔδ value; additionally, the corresponding analysis system can also be employed in the accurate determination of enantioselectivities of axial chiral quinolines.

## Results and Discussion

**FIGURE 1:**
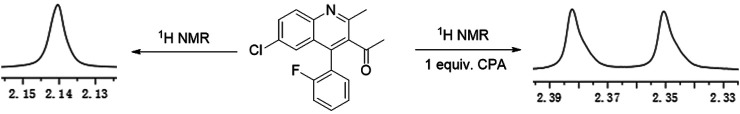
Chiral 1H NMR analysis of aryl quinolines with a chiral phosphoric acid.

As shown in Figure 1, the methyl peak on the benzyl position of racemic 1-(6-chloro-4-(2-fluorophenyl)-2-methylquinolin-3-yl) ethan-1-one **1a** is unimodal on 1H NMR spectrum in the absence of chiral phosphoric acid. Generally, the addition of 1 equivalent of chiral phosphoric acid brought obvious chemical shift nonequivalences of this methyl peak of **1a**, suggesting the strong chiral interaction between chiral phosphoric acids and 4-aryl quinoline. It was shown that the substituents on phosphoric acids had obvious influence on the recognition. For example, 3,3’-α-naphthyl–substituted phosphoric acid C1 afforded a baseline resolution and the largest chemical shift nonequivalence (ΔΔδ = 0.03) of a methyl H signal of **1a** in CD_3_OD at 25°С, while 3,3’-phenyl–substituted phosphoric acid C7 failed to differentiate atropisomers of **1a**. Besides, deuterated solvents also played an important role in chiral recognition. As shown in Table 1, chemical shift nonequivalence of methyl H of **1a**’s atropisomers was observed when CPA **C1** and **1a** were combined in CD_2_Cl_2_, acetone-D6, CD_3_CN, and C_6_D_6_, while highly polar solvent, such as DMF-D7 and DMSO-D6, seemed to break the interaction between the chiral sensor and analyte, resulting in no differentiation of atropisomers. Besides, different peaks overlapped together when CDCl_3_ was employed as solvent. Significantly, C_6_D_6_ enabled the best chiral recognition of up to ΔΔδ 0.1 ppm, albeit with poor solubility of CPA and quinoline analytes. Considering the fact that CPA and quinoline mixture dissolve well in CDCl_3_, binary solvents of CD_3_OD and CDCl_3_ (5/1) were chosen as analysis media in the purpose of balancing solubility and recognition, offering eminent solubility and baseline resolution (entry 16). Additionally, the amount of **1a** also influenced differentiation; for example, baseline resolution was not achieved when a 0.5 equivalent of chiral phosphoric acid C1 was used, while increasing the amount of C1 to 2 equivalent resulted in larger chemical shift nonequivalence (ΔΔδ = 0.05). Finally, under the balance of atom economy and recognition, 1 equivalent of (R)-C1 was employed as a chiral sensor (entry 17).

**TABLE 1:** Evaluating the chiral recognition abilities of chiral phosphoric acids (R)-C with 1a.^a^

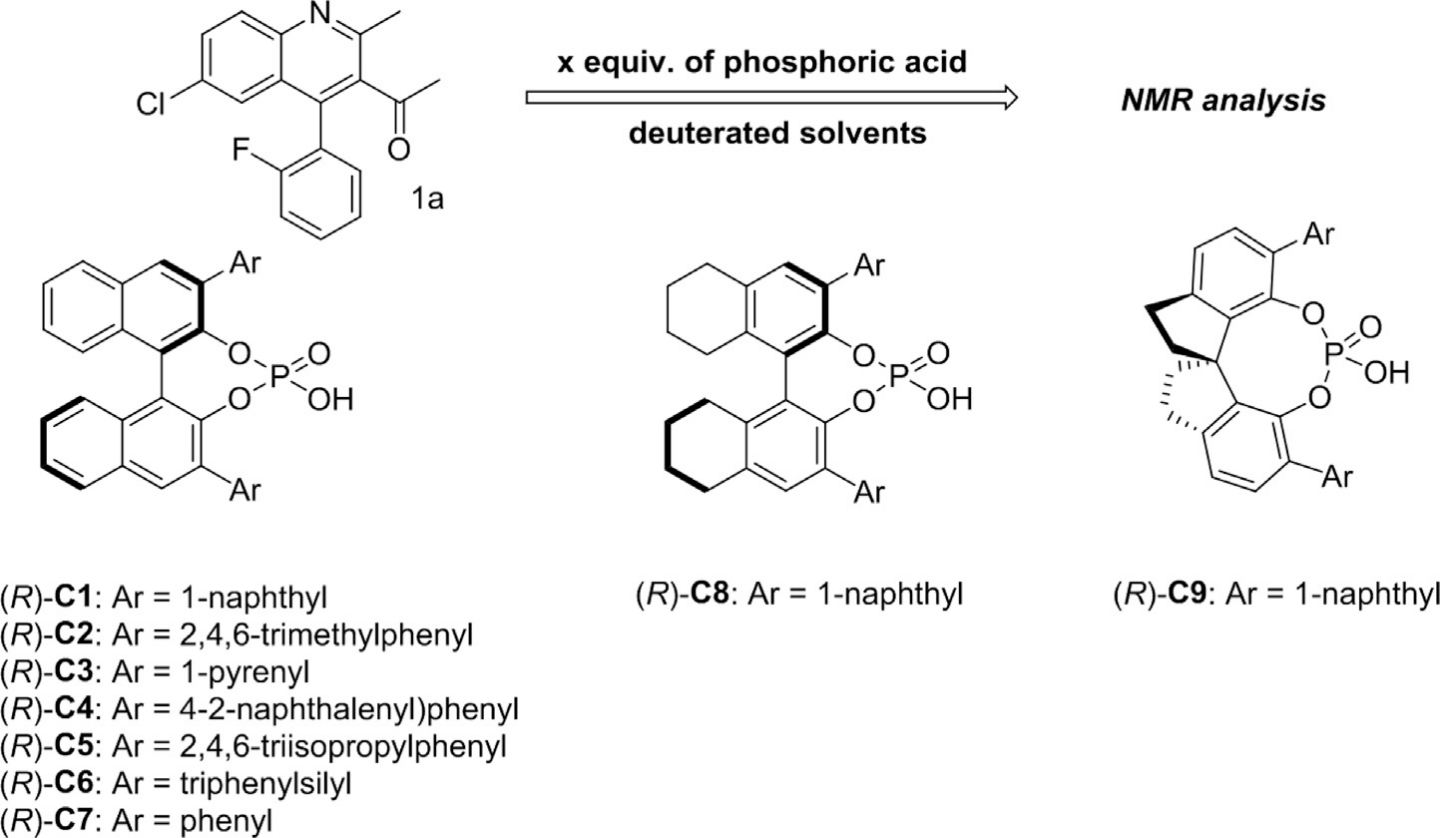

Entry	Chiral shift	Deuterated	ΔΔδ (ppm)
	Reagent	Solvents	
1	(R)-C1	CD_3_OD	0.03
2	(R)-C2	CD_3_OD	0.01
3	(R)-C3	CD_3_OD	0
4	(R)-C4	CD_3_OD	0
5	(R)-C5	CD_3_OD	0.01
6	(R)-C6	CD_3_OD	0
7	(R)-C7	CD_3_OD	0
8	(R)-C8	CD_3_OD	0.02
9	(R)-C9	CD_3_OD	0.01
10	(R)-C1	CDCl_3_	nd
11	(R)-C1	DMSO-D_6_	0
12	(R)-C1	DMF-D_7_	0
12	(R)-C1	Acetone-D_6_	0.02
14	(R)-C1	CD_3_CN	0.01
15	(R)-C1	C_6_D_6_	0.1
16	(R)-C1	CD_3_OD^b^	0.03
17	(R)-C1	CD_3_OD^c^	0.02
18	(R)-C1	CD_3_OD^d^	0.05

a Unless otherwise noted, all samples were prepared by mixing (R)-C (0.01 mmol) and the guests 2a (0.01 mmol) in CD_3_OD (0.5 ml) at 25°C.

b 0.1 ml CDCl_3_ was added.

c 0.5 equiv. of (R)-C1 was used.

d 2 equiv. of (R)-C1 was used.

Under optimized conditions, a series of 4-aryl quinoline guests were tested. First, the influence of substituents on quinoline (ring 1) was evaluated. It was shown that different electron-withdrawing groups on ring 1 were fit well under standard conditions, providing baseline resolutions and 0.02–0.17 ppm ΔΔδ values, respectively (Table 2, entries 1–5). Besides, different R3 groups on quinoline such as acetyl, ethyl formate, methyl formate and trifluoroacetyl were also tested, all of which led to clear recognition of atropisomers with up to 0.07 ppm ΔΔδ values. Subsequently, different 4-aryl groups (ring 2) were also studied. As shown in Table 2, a variety of electron-withdrawing or electron-donating groups on ring 2 were well-tolerated, and substituents with either moderate or bulky size on the 2’-position of ring 2 all resulted in clear baseline resolution with good chemical shift nonequivalences. Noticeably, when 1-{4-[(1,1’-biphenyl)-2-yl]-2-methylquinolin-3-yl} ethan-1-one **1g** was employed as analyte, the largest chemical shift nonequivalence of 0.17 ppm ΔΔδ was obtained. Interestingly, when **1k-1n** were employed as guests, obvious split peaks on α-H of oxygen were observed. It is also worth noting that nitro-substituted substrates **1b** and **1g** also afforded good differentiation results (chemical shift nonequivalence of 0.11 and 0.17 ppm ΔΔδ, respectively), possibly due to the steric hindrance effect of nitro group.

**TABLE 2:** Measurements of 1H chemical shift nonequivalences (DDd) of racemic aryl quinolinones.^a^

Entry	Aryl quinolinone	Spectra	ΔΔδ (ppm)
1^b^	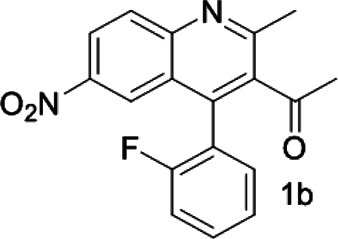	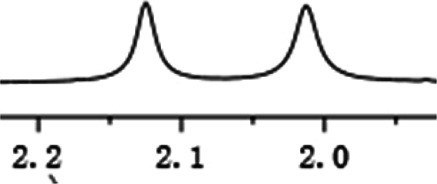	0.11
2	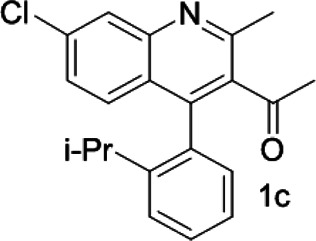	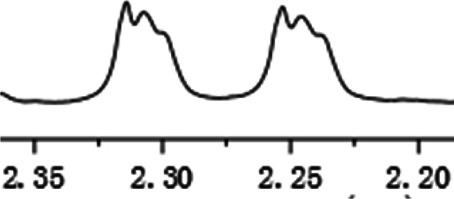	0.06
3^b^	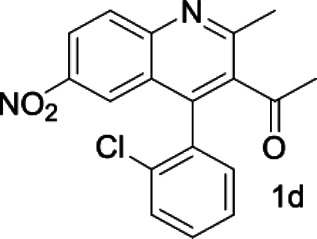	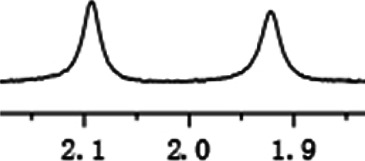	0.17
4	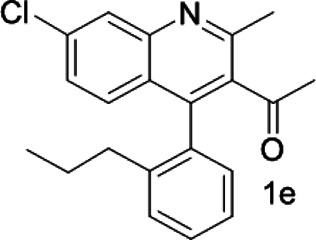	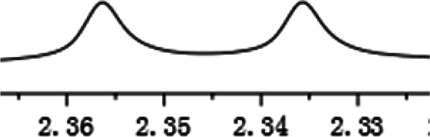	0.02
5^b^	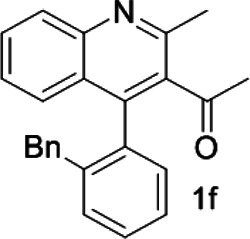	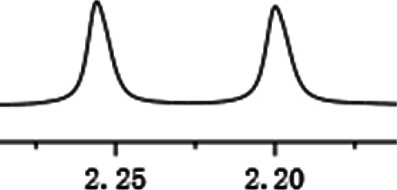	0.06
6^b^	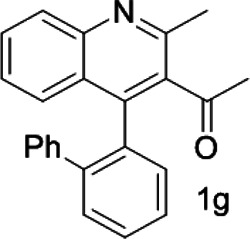	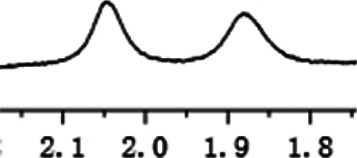	0.17
7	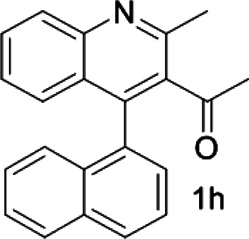	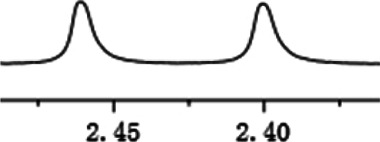	0.06
8^c^	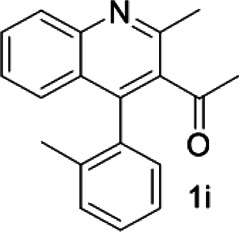	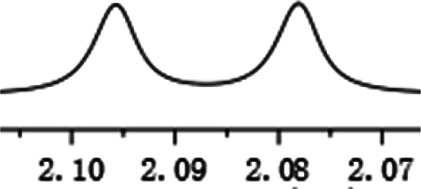	0.02
9	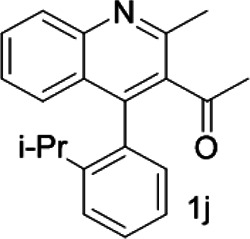	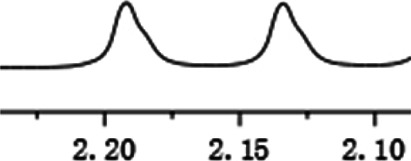	0.07
10	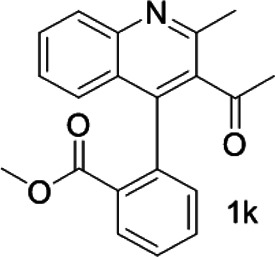	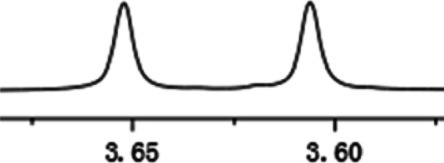	0.04
11^b^	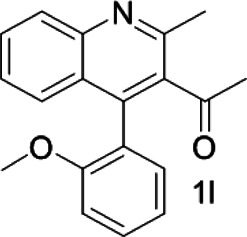	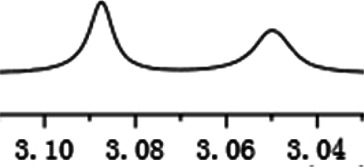	0.04
12	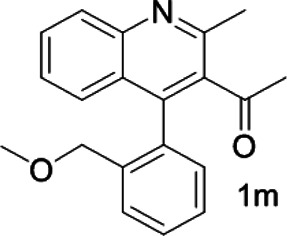	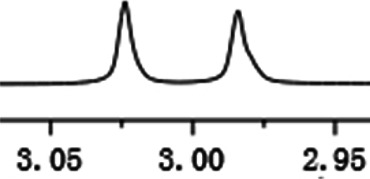	0.04
13	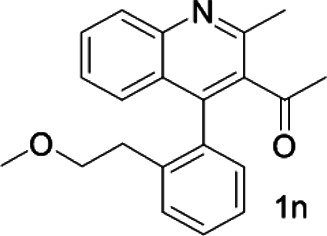	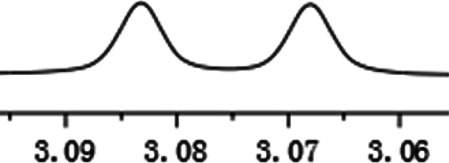	0.01
14	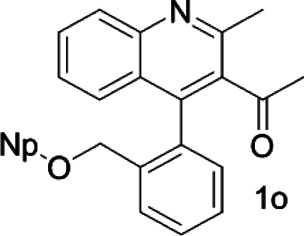	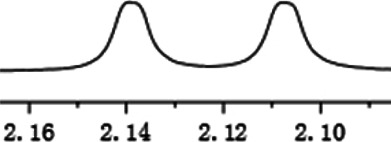	0.03
15	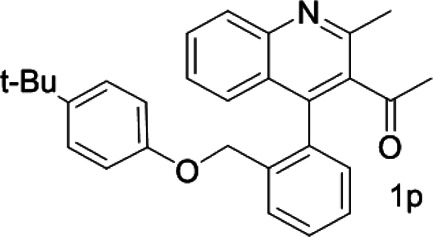	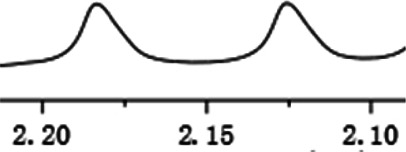	0.05
16	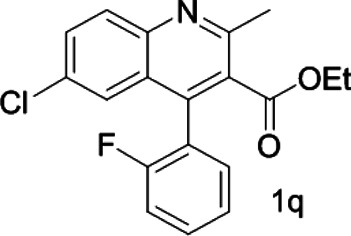	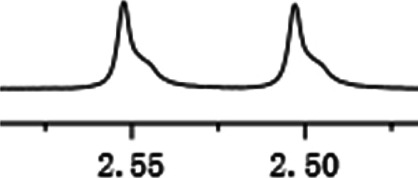	0.05
17	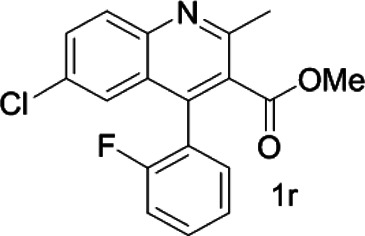	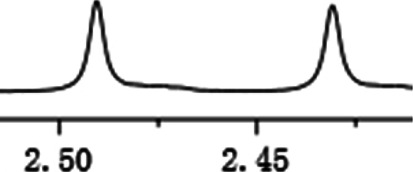	0.06
18^b^	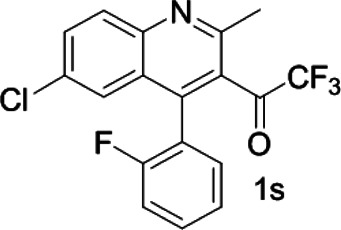	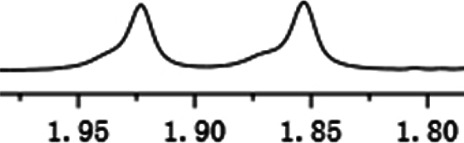	0.07

^a^Unless otherwise noted, all samples were prepared by mixing (R)-C1 (0.01 mmol) and the guests **2** (0.01 mmol) in CD_3_OD (0.5 ml) and CDCl_3_ (0.1 ml) at 25°C.

b 0.5 ml C_6_D_6_ was used.

c 2 equiv. of (R)-C1 was used.

**FIGURE 2:**
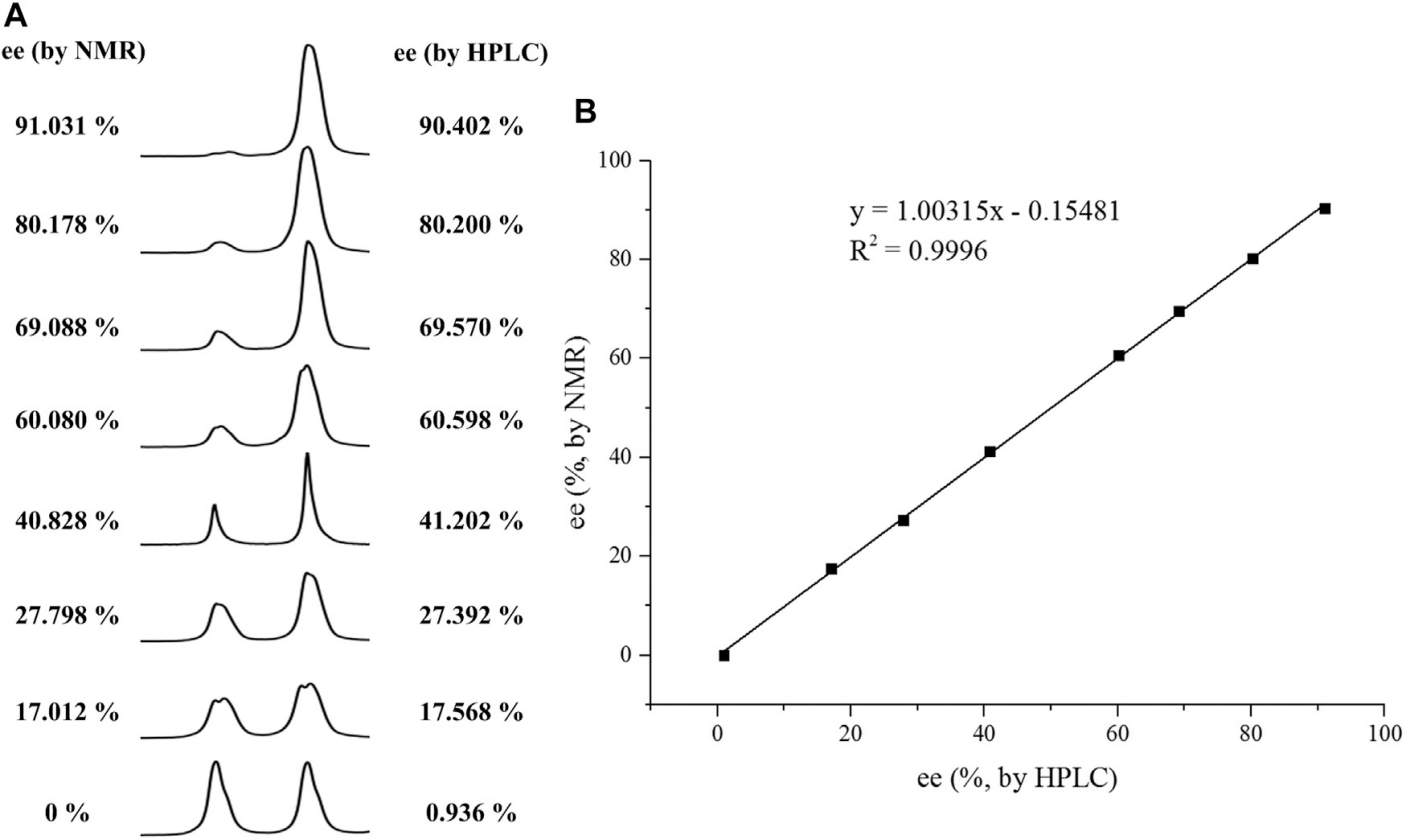
**(A)** Selected regions of the 1H NMR spectra of nonracemic aryl quinolinone samples (varied ee values) with (R)-C1 in 0.5 ml CD_3_OD and 0.1 ml CDCl_3_; **(B)** linear correlation between ee values determined by HPLC and NMR ee values, *R* ^2^ = correlation coefficient.

With this optimal recognition condition, the possibility of our methodology in the enantiomeric determination of various nonracemic **1j** samples was explored. As shown in Figure 2, **1j** samples with different enantiopurities was combined with 1 equivalent of CPA C1 and then monitored by NMR. It was revealed that the optical purities of **2a** could be accurately obtained by integrating the corresponding H signals of the methyl group of **1j**, which were very close to the exact results measured by HPLC. Compared with those data obtained from chiral HPLC analysis, an excellent linear relationship of a correlation coefficient *R* ^2^ 0.9996 and up to 0.03% absolute error was obtained.

## Conclusion

In conclusion, an efficient phosphoric acid–promoted chiral recognition of atropisomeric quinolines via NMR analysis was successfully developed. With this method, atropisomers of various quinolines were well-discriminated with base resolution; besides, the optical purities of different nonracemic quinoline **1j** could be evaluated fast with high accuracy. This method broadens the chiral analysis ability of chiral phosphoric acids, which encourages us to further explore the interaction of chiral acids with different analytes.

## Data Availability

The original contributions presented in the study are included in the article/Supplementary Material, further inquiries can be directed to the corresponding author.
