# Supplementary material for: Chiral Phosphoric Acid Promoted Chiral 1H NMR Analysis of Atropisomeric Quinolines
Source: Front Chem. 2021 Jun 10;9:672704. doi: 10.3389/fchem.2021.672704 (PMC8222546; doi:10.3389/fchem.2021.672704)

***Supporting information***

**Contents
1.** General information ................................................................................................. 2
**2.** General procedure for the synthesis of substrates **1a-1s** and analytic data............... 2 **3.** Procedure for the chiral analysis of Atropisomeric Aryl Quinolinones.................. 11
**4.** Evaluating the chiral recognition abilities of chiral sensors CPA with **1a**.............. 12
**5.** ^1^H NMR spectra of chiral sensors CPA and racemic **1a**......................................... 13
**6.** ^1^H NMR spectra of (*R*)-**C1** and racemic Aryl Quinolinones **1b-1s**........................ 22
**7.** 1H NMR spectra/HPLC data of (*R*)-**C1** and **1j** with different optical purities ...... 31

**8.** References................................................................................................................ 40

**9.** ^1^H and ^13^C NMR Spectra of Aryl Quinolinones **1a-1s**............................................. 41

1. **General:**

NMR spectra were recorded on Brucker-500MHz or Instruments Ltd.-400MHz spectrometers using tetramethylsilane as an internal reference. HRMS(Micromass GCT-MS) spectra were recorded on BRUKER micrOTOF-QII. HPLC analysis was performed on HITACHI Primaide liquid chromatography. Chiralpak AD-H were purchased from Daicel Chemical Industries, LTD. For HPLC analysis, the chiral HPLC methods were calibrated with the corresponding racemic mixtures. Bromobenzene, 2-Aminobenzophenone and other chemicals were purchased from the Adamas, Energy Chemical, Alfa Aesar, J&K and TCI, and used directly. THF, CH_2_Cl_2_ and EtOAc were distilled before use.

Catalysts (*R*)-**C1-7** were prepared according to the literature.^[1]^ Catalyst (*R*)-**C8** and (*R*)-**C9** were purchased from Daicel Chiral Technologies (China) CO., LTD. and used directly.

1. **General procedure for the synthesis of substrates 1a-1s and analytic data**

**2.1. General Procedure for the synthesis of Bromobenzenes^[2,3,4]^ :**

NaH (60% w/w dispersion in mineral oil, 1.2 equiv) in several portions was added into dry THF at 0°С under N_2_ atmosphere.Then the mixture were stirred 30 min and 2-bromobenzyl alcohol (1 equiv.) was added via syringe. The solution was then allowed to warm to r.t. and stir for another 18 h.After the reaction was completed, it was stopped by adding saturated aqueous NaCl. The crude mixture was extracted with EtOAc (3x20 mL) and the combined organic extracts were washed with brine, dried over anhydrous Na_2_SO_4_, and concentrated in vacuo. The residue was purified by column chromatography (eluent: 9:1 PE/EA) to afford the title compound as a colourless oil.

The mixture of 2-bromobenzylbromide (1 equiv.), phenol (2 equiv.), K_2_CO_3_ (3 equiv.), and acetone were stirred at room temperature for 18 h. After the reaction was completed the mixture was concentrated under reduced pressure and diluted with CH_2_Cl_2_ and H_2_O (1: 1), and then extracted with CH_2_Cl_2_ three times and Et_2_O one time. The combined organic layers were washed with brine, dried over anhydrous Na_2_SO_4_, and concentrated under reduced pressure. The product was purified by silica gel column chromatography (hexane/acetone = 50/1) to afford the title compound as a white powder.

In a dry flask under N_2_ atmosphere, t-BuOK (1.5 equiv.) was added to a suspension of Ethyltriphenylphosphonium bromide (1.5 equiv.) in dry THF at 0 °C and then the mixture was stirred for 30 min. 2-bromobenzaldehyde (1.0 equiv.) was added via syringe slowly and the reaction was stirred overnight at r.t.. Then the solvent was remove under reduced pressure, and PE was added to the obtained residue with vigorous stirring. The separated solid was filtered through a Buchner funnel and the solvent was evaporated under reduced pressure to give the crude material. The crude was purified by column chromatography (Petroleum ether) on silica gel affording 2-bromostyrene (E/Z) as a colorless oil.

The mixture of 2-bromostyrene (1 equiv.), TsNHNH_2_ (5 equiv.), NaOAc·3H_2_O (5 equiv.), and THF (0.3 M) were heated at reflux for 24 h. When the reaction was complete as determined by TLC, the reaction mixture was cooled to room temperature and H_2_O was added. The crude mixture was extracted with EtOAc (3x20) and the combined organic extracts were washed with brine, dried over anhydrous Na_2_SO_4_, and concentrated in vacuo. The crude was purified by column chromatography (Petroleum ether) on silica gel to give 1-bromo-2-propylbenzene as a colorless oil.

To a flame dried 1 neck round bottom flask containing a solution of 2-romobenzylbromide (3g, 12 mmol) in 36 mL of benzene was added a magnetic stir bar and AlCl_3_ ( 1.0g, 12 mmol) dissolved in 15 mL of nitrobenzene all conducted under N_2_ atmosphere. The mixture was then allowed to reflux for 6 h. Upon completion, the mixture was allowed to cool to room temperature and was extracted with Et_2_O (3 x 30 mL), dried over anhydrous Na_2_SO_4_, and evaporated under reduced pressure. The crude was purified by column chromatography (Petroleum ether) on silicagel to afford benzyl-2-bromobenzene as a colorless oil.

- 1. **General Procedure for the synthesis of** **2-Aminobenzophenones^[5]^:**

A mixture of 2-aminobenzophenone-2'-carboxylic acid (482.5 mg, 2.0 mmol), absolute MeOH (20 mL), and concd. H_2_SO_4_ (0.25 mL) refluxed for 16 h, then the reaction was stopped by adding saturated aqueous Na_2_CO_3_.The mixture was extracted with EA (3 x 20 mL), and the combined extracts were washed with saturated NaCl, dried over anhydrous Na_2_SO_4_ and concentrated in vacuo. The crude was purified by column chromatography (PE/EA =10: 1) on silica gel to give 2-aminobenzophenones.

To a solution of N,O-dimethylhydroxylamine hydrochloride (2.0 equiv.) in 90% aqueous ethanol was added triethlyamine (2.0 equiv.) and after 20 min stirring at room temperature isatonic anhydride 1 (1.0 equiv) in several portions was added into the reaction mixture. After that, this solution reflux for 3 h. After the reaction was complete as determined by TLC, the reaction was stopped by poured onto an equivual volume of ice and saturated aqueous Na_2_CO_3_ and stir with a glass rod. The separated solid was filtered through a Buchner funnel.Then the ethanol was removed under reduce pressure and the resulting aqueous mixture was extracted with EtOAc three times and the combined organic extracts were washed with brine, dried over anhydrous Na_2_SO_4_, and concentrated in vacuo. The crude was purified by column chromatography (PE/EA = 2:1) on silicagel to give 2.

The mixture of 2 (1.0 equiv.), aryl bromide (1.0 equiv.) and dry THF in a flamed-dried schlenk tubewere stir at -78°С under N_2_ atmosphere for 30 min. Then n-BuLi (1.6 mol/L in hexene, 2.0 equiv) was added dropwise into the reaction mixture over 30 min and stirred at this temperature for another 3h. Then the reaction was stopped by adding 1.0 N HCl (2.0 equiv) at -78 °C. The solution was then allowed to warm to rt and the mixture was extracted with EA (3 x 20 mL), and the combined extracts were washed with saturated Na_2_CO_3_, dried over anhydrous Na_2_SO_4_ and concentrated in vacuo. The crude was purified by column chromatography (PE/EA = 30:1 to 15:1) on silicagel to give 2-aminobenzophenones 3.

- 1. **General Procedure for the synthesis of** **Aryl Quinolinones^[6]^ :**

The mixture of 2-Aminobenzophenones (1.0 equiv.), an appropriate carbonyl component (1.0 equiv) and dry DMF in a flamed-dried schlenk tube were stir at 80°С overnight. After cooling to rt, the reaction was poured onto an equivual volume of H_2_O.

The mixture was extracted with EA (3 x 20 mL), and the combined extracts were washed with saturated NaCl, dried over anhydrous Na_2_SO_4_ and concentrated in vacuo. The crude was purified by column chromatography (PE/EA = 6:1) on silicagel to give Aryl Quinolinones.

1-(6-chloro-4-(2-fluorophenyl)-2-methylquinolin-3-yl)ethan-1-one **1a** was obtained as a white solid.^1^H NMR (500 MHz, CDCl_3_) δ 8.02 (d, *J* = 8.9 Hz, 1H), 7.66 (dd, *J* = 9.0, 2.3 Hz, 1H), 7.57-7.53 (m, 1H), 7.40-7.38 (m, 1H), 7.33-7.22 (m, 3H), 2.70 (s, 3H), 2.14 (s, 3H).^13^C NMR (126 MHz, CDCl_3_) δ 204.33, 160.36, 158.39, 153.90, 145.74, 137.38, 136.32, 132.80, 132.11, 132.09, 131.68, 131.62, 131.16, 130.65, 125.86, 124.85, 124.82, 124.50, 122.15, 122.02, 116.27, 116.10, 77.26, 77.00, 76.75, 31.40, 23.78. HRMS (BRUKER micrOTOF-QII) calcd for C_18_H_13_ClFNONa (M+Na)^+^ 336.0568, found 336.0568.

1-(4-(2-fluorophenyl)-2-methyl-6-nitroquinolin-3-yl)ethan-1-one **1b** was obtained as a brown solid. ^1^H NMR (500 MHz, CDCl_3_) δ 8.50 (d, *J* = 9.2 Hz, 1H), 8.39 (s, 1H), 8.21 (d, *J* = 9.1 Hz, 1H), 7.63-7.59 (m, 1H), 7.38-7.31 (m, 2H), 7.29-7.27 (m, 1H), 2.76 (s, 3H), 2.15 (s, 3H), 1.60 (s, 3H). ^13^C NMR (126 MHz, CDCl_3_) δ 203.57, 157.96, 149.31, 145.92, 139.96, 137.16, 132.34, 132.28, 132.01, 130.90, 125.15, 124.39, 123.74, 122.67, 116.53, 116.36, 77.24, 76.99, 76.74, 31.29, 24.20. HRMS (BRUKER micrOTOF-QII) calcd for C_18_H_14_FN_2_O_3_ (M+H)^+^ 325.0983, found 325.0985.

1-(7-chloro-4-(2-isopropylphenyl)-2-methylquinolin-3-yl)ethan-1-one **1c** was obtained as a yellow solid. ^1^H NMR (500 MHz, CDCl_3_) δ 8.08 (d, *J* = 1.8 Hz, 1H), 7.51-7.47 (m, 2H), 7.35 (dd, *J* = 8.9, 1.9 Hz, 1H), 7.30-7.27 (m, 2H), 7.10 (d, *J* = 7.7 Hz, 1H), 2.69 (s, 3H), 2.48-2.42 (m, 1H), 2.11 (s, 3H), 1.15 (d, *J* = 6.8 Hz, 3H), 0.93 (d, *J* = 6.8 Hz, 3H). ^13^C NMR (126 MHz, CDCl_3_) δ 204.58, 155.05, 147.74, 143.77, 136.16, 135.27, 132.81, 129.76, 129.64, 127.88, 127.84, 127.31, 126.46, 125.68, 124.39, 32.03, 30.45, 24.92, 23.94, 22.94. HRMS (BRUKER micrOTOF-QII) calcd for C_21_H_20_ClNONa (M+Na)^+^ 360.1131, found 360.1132.

1-(4-(2-chlorophenyl)-2-methyl-6-nitroquinolin-3-yl)ethan-1-one **1d** was obtained as a yellow solid. ^1^H NMR (500 MHz, Chloroform-*d*) δ 8.51-8.45 (m, 1H), 8.27-8.15 (m, 2H), 7.63 (d, *J* = 7.5 Hz, 1H), 7.55 (t, *J* = 7.8 Hz, 1H), 7.47 (t, *J* = 7.4 Hz, 1H), 7.28-7.26 (m, 1H), 2.76 (s, 3H), 2.16 (s, 3H). ^13^C NMR (126 MHz, CDCl_3_) δ 203.52, 158.14, 149.27, 145.93, 142.97, 136.58, 133.24, 132.57, 132.01, 131.42, 130.93, 130.31, 127.64, 124.01, 123.71, 122.73, 77.24, 76.99, 76.73, 31.11, 24.22. HRMS (BRUKER micrOTOF-QII) calcd for C_18_H_13_ClN_2_O_3_Na (M+Na)^+^ 363.0513, found 363.0515.

1-(7-chloro-2-methyl-4-(2-propylphenyl)quinolin-3-yl)ethan-1-one **1e** was obtained as a yellow solid. ^1^H NMR (500 MHz, CDCl_3_) δ 8.08 (d, *J* = 1.8 Hz, 1H), 7.45 (t, *J* = 7.5 Hz, 1H), 7.40 (d, *J* = 7.5 Hz, 1H), 7.35 (dd, *J* = 8.9, 1.9 Hz, 1H), 7.30 (t, *J* = 7.7 Hz, 2H), 7.12 (d, *J* = 7.2 Hz, 1H), 2.69 (s, 3H), 2.29-2.17 (m, 2H), 2.06 (s, 3H), 1.47-1.35 (m, 2H), 0.71 (t, *J* = 7.3 Hz, 3H). ^13^C NMR (126 MHz, CDCl_3_) δ 204.63, 155.14, 147.76, 143.72, 141.18, 136.16, 135.18, 133.78, 129.96, 129.37, 129.34, 127.90, 127.66, 127.44, 125.88, 124.10, 35.07, 31.73, 23.94, 23.13, 13.96. HRMS (BRUKER micrOTOF-QII) calcd for C_21_H_20_ClNONa (M+Na)^+^ 360.1131, found 360.1134.

1-(4-(2-benzylphenyl)-2-methylquinolin-3-yl)ethan-1-one **1f** was obtained as a white solid. ^1^H NMR (500 MHz, Chloroform-*d*) δ 8.06 (d, *J* = 8.4 Hz, 1H), 7.72 – 7.65 (m, 1H), 7.41 (t, *J* = 7.5 Hz, 1H), 7.33 (q, *J* = 7.8, 7.3 Hz, 2H), 7.30-7.25 (m, 2H), 7.18 (d, *J* = 7.5 Hz, 1H), 7.08-7.02 (m, 3H), 6.76 (d, *J* = 7.5 Hz, 2H), 3.71-3.57 (m, 2H), 2.70 (s, 3H), 1.94 (s, 3H). ^13^C NMR (126 MHz, CDCl_3_) δ 205.30, 153.61, 147.34, 143.08, 140.22, 139.36, 135.16, 134.52, 130.27, 130.14, 130.07, 129.25, 129.19, 128.82, 128.16, 126.49, 126.31, 126.13, 126.06, 125.25, 77.25, 76.99, 76.74, 39.55, 31.52, 23.85. HRMS (BRUKER micrOTOF-QII) calcd for C_25_H_21_NONa (M+Na)^+^ 374.1521, found 374.1520.

1-(4-([1,1'-biphenyl]-2-yl)-2-methylquinolin-3-yl)ethan-1-one **1g** was obtained as a yellow solid.^1^H NMR (500 MHz, CDCl_3_) δ 7.98 (d, *J* = 8.5 Hz, 1H), 7.63 (t, *J* = 7.6 Hz, 1H), 7.60-7.56 (m, 2H), 7.48-7.45 (m, 2H), 7.34 (t, *J* = 7.6 Hz, 1H), 7.30 (d, *J* = 7.6 Hz, 1H), 7.09-7.01 (m, 5H), 2.62 (s, 3H), 2.06 (s, 3H).^13^C NMR (126 MHz, CDCl_3_) δ 204.34, 153.63, 147.16, 144.14, 141.64, 139.95, 135.15, 133.58, 131.13, 130.86, 129.93, 129.43, 128.77, 128.72, 127.95, 127.22, 127.16, 126.62, 126.35, 125.60, 77.24, 76.99, 76.73, 31.63, 24.06. HRMS (BRUKER micrOTOF-QII) calcd for C_24_H_19_NONa (M+Na)^+^ 360.1365, found 336.1374.

1-(2-methyl-4-(naphthalen-1-yl)quinolin-3-yl)ethan-1-one **1h** was obtained as a white solid. ^1^H NMR (500 MHz, Chloroform-*d*) δ 8.12 (d, *J* = 8.4 Hz, 1H), 8.00 (d, *J* = 8.3 Hz, 1H), 7.95 (d, *J* = 8.2 Hz, 1H), 7.70 (t, *J* = 7.6 Hz, 1H), 7.58 (t, *J* = 7.6 Hz, 1H), 7.51 (t, *J* = 7.5 Hz, 1H), 7.40 (d, *J* = 7.0 Hz, 1H), 7.36 (t, *J* = 7.6 Hz, 1H), 7.31-7.25 (m, 2H), 7.20 (d, *J* = 8.3 Hz, 1H), 2.75 (s, 3H), 1.88 (s, 3H). ^13^C NMR (126 MHz, CDCl_3_) δ 205.03, 153.91, 147.32, 142.70, 135.92, 133.40, 132.71, 131.84, 130.16, 129.45, 128.92, 128.71, 128.50, 126.92, 126.59, 126.56, 126.42, 125.96, 125.83, 125.38, 77.25, 77.00, 76.74, 31.56, 23.94. HRMS (BRUKER micrOTOF-QII) calcd for C_22_H_17_NONa (M+Na)^+^ 334.1208, found 334.1204.

1-(2-methyl-4-(o-tolyl)quinolin-3-yl)ethan-1-one **1i** was obtained as a yellow solid. ^1^H NMR (500 MHz, CDCl_3_) δ 8.08 (d, *J* = 8.4 Hz, 1H), 7.71 (t, *J* = 7.6 Hz, 1H), 7.41 (q, *J* = 7.0 Hz, 2H), 7.37-7.28 (m, 3H), 7.15 (d, *J* = 7.4 Hz, 1H), 2.71 (s, 3H), 2.08 (s, 3H), 2.00 (s, 3H). ^13^C NMR (126 MHz, CDCl_3_) δ 205.01, 153.76, 147.38, 143.71, 136.68, 134.98, 134.70, 130.48, 130.10, 129.93, 129.09, 128.96, 126.61, 126.00, 125.96, 125.09, 77.25, 77.00, 76.74, 31.58, 23.87, 19.86. HRMS (BRUKER micrOTOF-QII) calcd for C_19_H_17_NONa (M+Na)^+^ 298.1208, found 298.1208.

1-(4-(2-isopropylphenyl)-2-methylquinolin-3-yl)ethan-1-one **1j** was obtained as a white solid. ^1^H NMR (500 MHz, Chloroform-*d*) δ 8.07 (d, *J* = 8.4 Hz, 1H), 7.71 (t, *J* = 7.6 Hz, 1H), 7.48 (d, *J* = 4.3 Hz, 2H), 7.40 (t, *J* = 7.5 Hz, 1H), 7.35 (d, *J* = 8.3 Hz, 1H), 7.30-7.27 (m, 1H), 7.12 (d, *J* = 7.5 Hz, 1H), 2.71 (s, 3H), 2.52-2.47 (m, 1H), 2.12 (s, 3H), 1.15 (d, *J* = 6.8 Hz, 3H), 0.94 (d, *J* = 6.8 Hz, 3H). ^13^C NMR (126 MHz, CDCl_3_) δ 204.98, 153.60, 147.80, 147.32, 143.76, 135.16, 133.33, 130.11, 129.76, 129.52, 128.80, 126.53, 126.33, 126.29, 125.90, 125.54, 77.24, 76.99, 76.74, 32.10, 30.43, 24.90, 23.92, 22.97. HRMS (BRUKER micrOTOF-QII) calcd for C_21_H_21_NONa (M+Na)^+^ 326.1521, found 326.1525.

methyl 2-(3-acetyl-2-methylquinolin-4-yl)benzoate **1k** was obtained as a white solid. ^1^H NMR (500 MHz, Chloroform-*d*) δ 8.13 (d, *J* = 7.7 Hz, 1H), 8.07 (d, *J* = 8.5 Hz, 1H), 7.69-7.58 (m, 3H), 7.38 (t, *J* = 7.6 Hz, 1H), 7.28-7.25 (m, 2H), 3.49 (s, 3H), 2.71 (s, 3H), 2.09 (s, 3H). ^13^C NMR (126 MHz, CDCl_3_) δ 205.35, 166.51, 153.37, 147.02, 144.10, 136.20, 133.92, 132.35, 131.89, 130.99, 130.66, 129.84, 129.09, 128.95, 126.51, 125.30, 125.13, 77.25, 77.00, 76.74, 52.05, 31.47, 23.89. HRMS (BRUKER micrOTOF-QII) calcd for C_20_H_17_NO_3_Na (M+Na)^+^ 342.1106, found 342.1113.

1-(4-(2-methoxyphenyl)-2-methylquinolin-3-yl)ethan-1-one **1l** was obtained as a yellow solid. ^1^H NMR (500 MHz, Chloroform-*d*) δ 8.06 (d, *J* = 8.4 Hz, 1H), 7.70-7.67 (m, 1H), 7.48 (t, *J* = 7.8 Hz, 1H), 7.43-7.37 (m, 2H), 7.16-7.11 (m, 1H), 7.10-7.02 (m, 2H), 3.71 (s, 3H), 2.71 (s, 3H), 2.09 (s, 3H). ^13^C NMR (126 MHz, CDCl_3_) δ 205.17, 156.75, 153.64, 147.31, 141.69, 135.28, 131.74, 130.66, 129.85, 128.78, 126.22, 126.19, 125.51, 124.11, 120.95, 111.07, 77.25, 76.99, 76.74, 55.44, 31.25, 23.90. HRMS (BRUKER micrOTOF-QII) calcd for C_19_H_17_NO_2_Na (M+Na)^+^ 414.1157, found 414.1154.

1-(4-(2-(methoxymethyl)phenyl)-2-methylquinolin-3-yl)ethan-1-one **1m** was obtained as a white solid. ^1^H NMR (500 MHz, Chloroform-*d*) δ 8.08 (d, *J* = 8.4 Hz, 1H), 7.72 (t, *J* = 7.7 Hz, 1H), 7.64 (d, *J* = 7.8 Hz, 1H), 7.52 (t, *J* = 7.6 Hz, 1H), 7.43-7.39 (m, 2H), 7.33 (d, *J* = 8.4 Hz, 1H), 7.18 (d, *J* = 7.5 Hz, 1H), 4.14-4.02 (m, 2H), 3.12 (s, 3H), 2.71 (s, 3H), 2.10 (s, 3H). ^13^C NMR (126 MHz, CDCl_3_) δ 205.10, 153.60, 147.28, 142.53, 137.34, 135.12, 133.49, 130.18, 130.05, 129.29, 128.88, 128.29, 127.57, 126.64, 126.02, 125.20, 77.24, 76.99, 76.73, 72.07, 58.46, 31.80, 23.87. HRMS (BRUKER micrOTOF-QII) calcd for C_20_H_19_NO_2_Na (M+Na)^+^ 328.1314, found 328.1317.

1-(4-(2-(2-methoxyethyl)phenyl)-2-methylquinolin-3-yl)ethan-1-one **1n** was obtained as a yellow solid. ^1^H NMR (500 MHz, CDCl_3_) δ 8.07 (d, *J* = 8.4 Hz, 1H), 7.71 (t, *J* = 7.6 Hz, 1H), 7.47-7.44 (m, 2H), 7.41 (t, *J* = 7.6 Hz, 1H), 7.37-7.31 (m, 2H), 7.17 (d, *J* = 7.5 Hz, 1H), 3.40-3.33 (m, 2H), 3.10 (s, 3H), 2.70 (s, 3H), 2.57 (t, *J* = 6.8 Hz, 2H), 2.10 (s, 3H). ^13^C NMR (126 MHz, CDCl_3_) δ 205.07, 153.61, 147.37, 143.28, 138.04, 135.12, 134.71, 130.13, 130.11, 129.79, 129.17, 128.88, 126.50, 126.27, 126.25, 125.56, 77.25, 76.99, 76.74, 72.02, 58.25, 33.16, 31.82, 23.85. HRMS (BRUKER micrOTOF-QII) calcd for C_21_H_21_NO_2_Na (M+Na)^+^ 342.1470, found 342.1469.

1-(2-methyl-4-(2-((naphthalen-1-yloxy)methyl)phenyl)quinolin-3-yl)ethan-1-one **1o** was obtained as a white solid. ^1^H NMR (500 MHz, Chloroform-*d*) δ 8.06 (d, *J* = 8.5 Hz, 1H), 8.00 (d, *J* = 8.4 Hz, 1H), 7.85 (d, *J* = 7.8 Hz, 1H), 7.73-7.67 (m, 2H), 7.58 (t, *J* = 7.6 Hz, 1H), 7.48 (t, *J* = 7.5 Hz, 1H), 7.45-7.32 (m, 5H), 7.26 (d, *J* = 4.3 Hz, 1H), 7.18 (t, *J* = 8.0 Hz, 1H), 6.52 (d, *J* = 7.6 Hz, 1H), 4.97-4.85 (m, 2H), 2.70 (s, 3H), 2.14 (s, 3H). ^13^C NMR (126 MHz, CDCl_3_) δ 205.14, 154.01, 153.53, 147.37, 142.13, 136.17, 135.23, 134.44, 133.69, 130.29, 130.17, 129.52, 129.00, 128.78, 128.00, 127.37, 126.86, 126.28, 125.91, 125.61, 125.54, 125.16, 125.08, 121.87, 120.56, 105.17, 77.25, 77.00, 76.75, 67.79, 31.97, 23.84. HRMS (BRUKER micrOTOF-QII) calcd for C_29_H_23_NO_2_Na (M+Na)^+^ 440.1627, found 440.1622.

1-(4-(2-((4-(tert-butyl)phenoxy)methyl)phenyl)-2-methylquinolin-3-yl)ethan-1-one **1p** was obtained as a white solid. ^1^H NMR (500 MHz, Chloroform-*d*) δ 8.06 (d, *J* = 8.5 Hz, 1H), 7.73-7.69 (m, 2H), 7.54 (t, *J* = 7.6 Hz, 1H), 7.46-7.36 (m, 3H), 7.23 (d, *J* = 7.5 Hz, 1H), 7.14 (d, *J* = 8.7 Hz, 2H), 6.55 (d, *J* = 8.7 Hz, 2H), 4.74-4.63 (m, 2H), 2.70 (s, 3H), 2.15 (s, 3H), 1.23 (s, 9H). ^13^C NMR (126 MHz, CDCl_3_) δ 205.18, 156.13, 153.50, 147.31, 143.73, 142.20, 136.27, 135.20, 133.70, 130.22, 130.15, 129.41, 128.95, 128.79, 127.94, 126.73, 126.05, 125.98, 125.15, 114.08, 77.25, 77.00, 76.74, 67.86, 33.99, 31.93, 31.43, 23.83. HRMS (BRUKER micrOTOF-QII) calcd for C_29_H_29_NO_2_Na (M+Na)^+^ 446.2096, found 446.2096.

ethyl 6-chloro-4-(2-fluorophenyl)-2-methylquinoline-3-carboxylate **1q** was obtained as a white solid. ^1^H NMR (500 MHz, CDCl_3_) δ 8.02 (d, *J* = 9.0 Hz, 1H), 7.66 (dd, *J* = 8.9, 1.8 Hz, 1H), 7.53-7.48 (m, 1H), 7.41 (s, 1H), 7.28-7.23 (m, 3H), 4.12-4.07 (m, 2H), 2.80 (s, 3H), 0.98 (t, *J* = 7.1 Hz, 3H). ^13^C NMR (126 MHz, CDCl_3_) δ 167.53, 160.68, 158.70, 155.34, 145.98, 139.99, 132.68, 131.34, 131.20, 131.18, 131.12, 131.06, 130.65, 128.68, 125.88, 124.79, 124.26, 124.23, 122.88, 122.74, 115.91, 115.74, 77.28, 77.03, 76.77, 61.49, 23.87, 13.59. HRMS (BRUKER micrOTOF-QII) calcd for C_19_H_15_ClFNO_2_Na (M+Na)^+^ 366.0673, found 366.0673.

methyl 6-chloro-4-(2-fluorophenyl)-2-methylquinoline-3-carboxylate **1r** was obtained as a yellow solid. ^1^H NMR (500 MHz, Chloroform-*d*) δ 8.03 (d, *J* = 9.0 Hz, 1H), 7.67 (dd, *J* = 8.9, 2.0 Hz, 1H), 7.54-7.49 (m, 1H), 7.41 (s, 1H), 7.31-7.23 (m, 4H), 3.61 (s, 3H), 2.78 (s, 3H). ^13^C NMR (126 MHz, CDCl_3_) δ 168.09, 160.55, 158.57, 155.29, 146.05, 140.21, 132.75, 131.45, 131.19, 131.13, 131.03, 131.01, 130.66, 128.51, 125.81, 124.86, 124.29, 124.26, 122.78, 122.65, 115.96, 115.79, 77.25, 76.99, 76.74, 52.27, 23.90. HRMS (BRUKER micrOTOF-QII) calcd for C_18_H_14_ClFNO_2_ (M+H)^+^ 330.0692, found 330.0692.

1-(6-chloro-4-(2-fluorophenyl)-2-methylquinolin-3-yl)-2,2,2-trifluoroethan-1-one **1s** was obtained as a white solid. ^1^H NMR (500 MHz, Chloroform-*d*) δ 8.07 (d, *J* = 8.9 Hz, 1H), 7.74 (d, *J* = 8.5 Hz, 1H), 7.59-7.54 (m, 1H), 7.48 (s, 1H), 7.32-7.20 (m, 3H), 2.71 (s, 3H). ^13^C NMR (126 MHz, CDCl_3_) δ 188.49, 160.56, 158.57, 153.80, 146.59, 141.04, 133.50, 132.40, 132.35, 132.28, 131.98, 130.86, 129.11, 125.20, 124.70, 116.36, 116.19, 77.24, 76.99, 76.73, 23.77. HRMS (BRUKER micrOTOF-QII) calcd for C_18_H_11_ClF_4_NO (M+H)^+^ 368.0460, found 368.0463.

**3.** **Procedure for the chiral analysis of** **Atropisomeric** **Aryl Quinolinones**

Phosphoric acid (*R*)-**C1** (0.01 mmol) and the Aryl Quinolinones (0.01 mmol) were mixed in CDCl3 (0.1 mL) and CD_3_OD (0.5 mL) or C6D6 (0.6 mL), and ^1^H NMR data were collected on a 500 MHz or 400 MHz spectrometer at 25 °C. Chiral HPLC analyses were performed on a HITACHI Primaide liquid chromatography (Daicel Chiralpak AD-H, hexane/iso-propanol=90/10, flow rate 1.0mL/min).

**4.** **Evaluating the chiral recognition abilities of chiral sensors** (*R*)-**C1 -** (*R*)-**C9 (0.01 mmol) with 1a (0.01 mmol) in CD_3_OD at 25°С.**

**5. ^1^H NMR spectra of chiral sensors (*R*)-C1 - (*R*)-C9 (0.01 mmol, 1.0 equiv.) and racemic Aryl Quinolinones (0.01 mmol, 1.0 equiv.).**

**5.1. ^1^H NMR (400 MHz, CDCl3) of racemic 1a.**

**
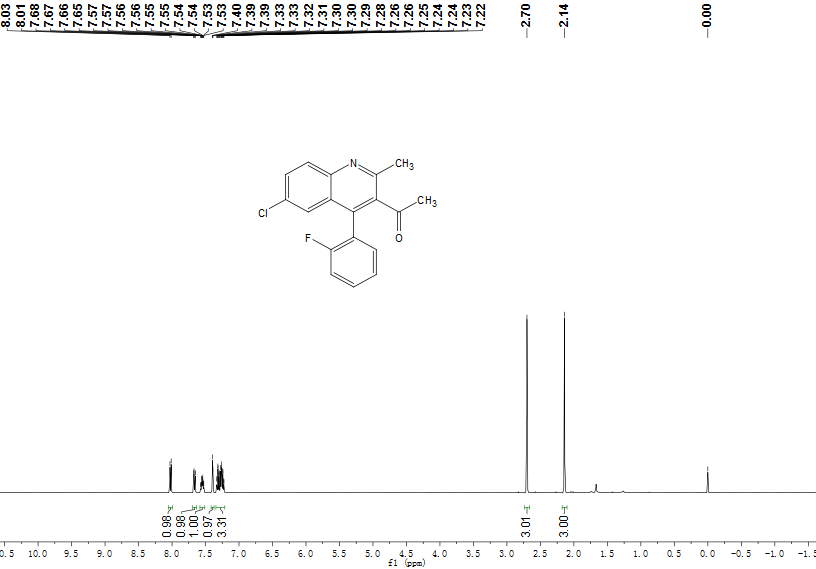
**

**5.2. ^1^H NMR (400 MHz, CD_3_OD) of (*R*)-C1 and guest 1a.**


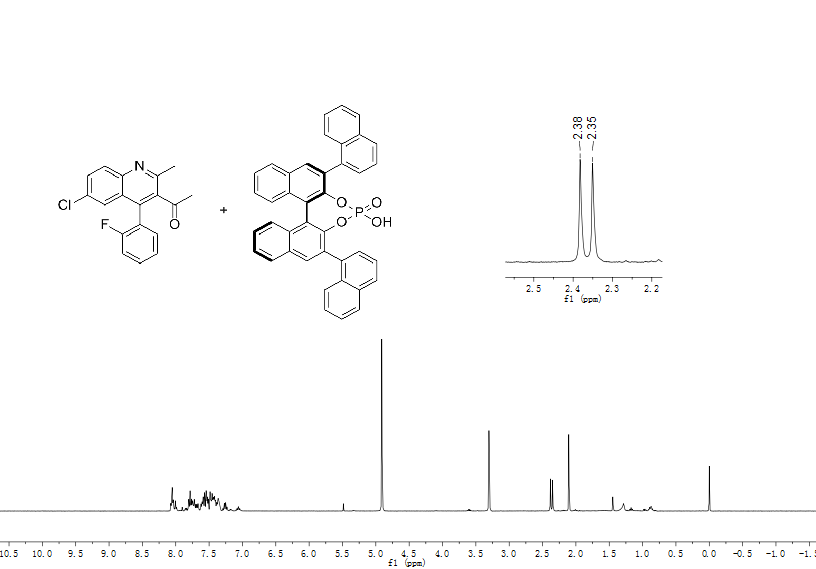


**5.3. ^1^H NMR (400 MHz, CD_3_OD) of (*R*)-C2 and guest 1a.**

**
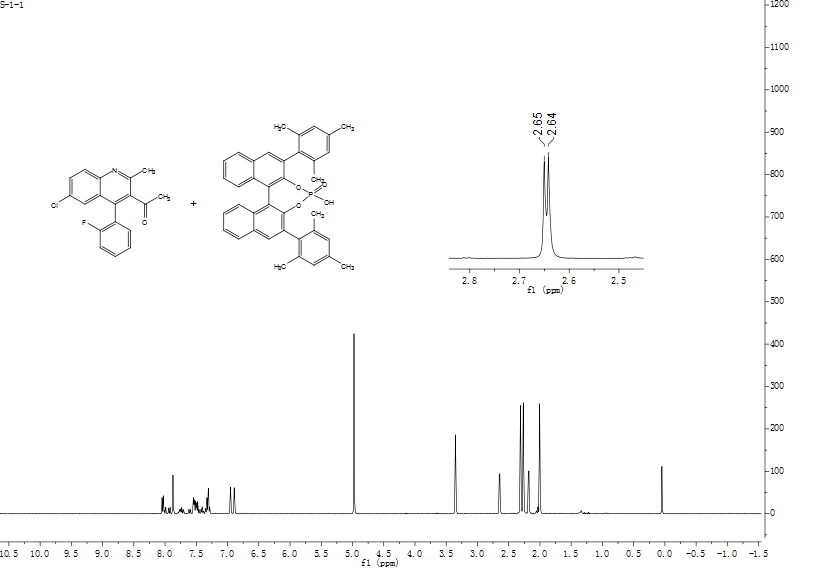
**

**5.4. ^1^H NMR (400 MHz, CD_3_OD) of (*R*)-C3 and guest 1a.**

**
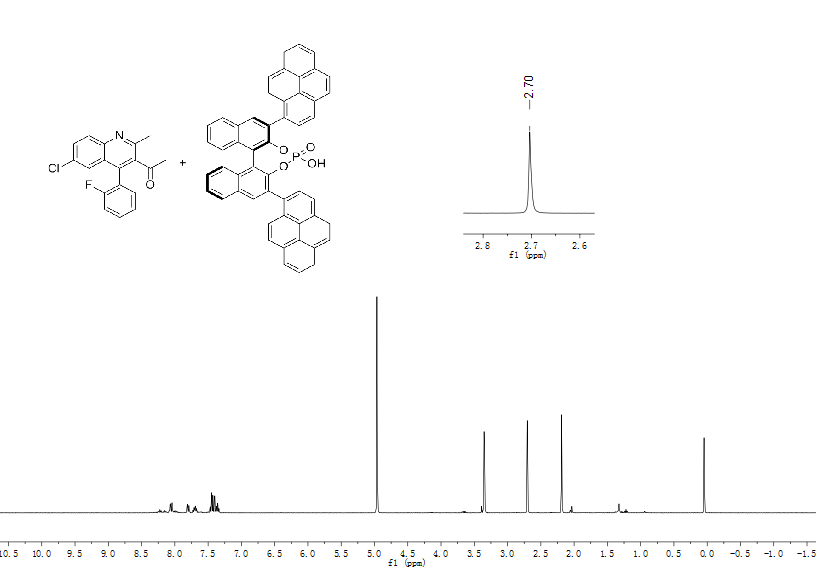
**

**5.5. ^1^H NMR (400 MHz, CD_3_OD) of (*R*)-C4 and guest 1a.**


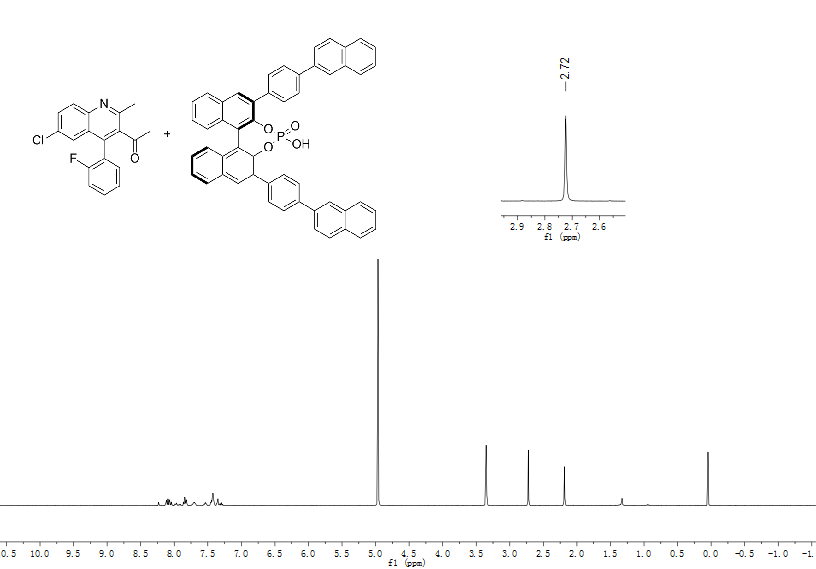


**5.6. ^1^H NMR (400 MHz, CD_3_OD) of (*R*)-C5 and guest 1a.**


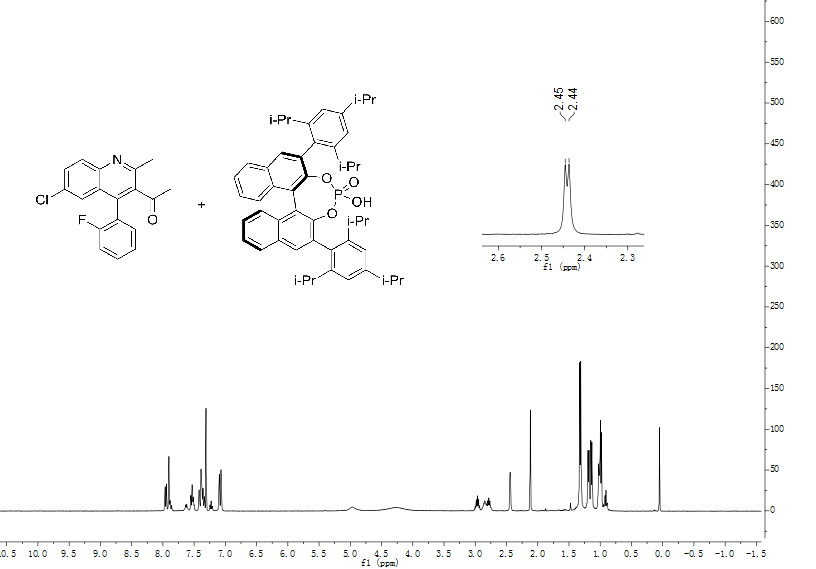


**5.7. ^1^H NMR (400 MHz, CD_3_OD) of (*R*)-C6 and guest 1a.**


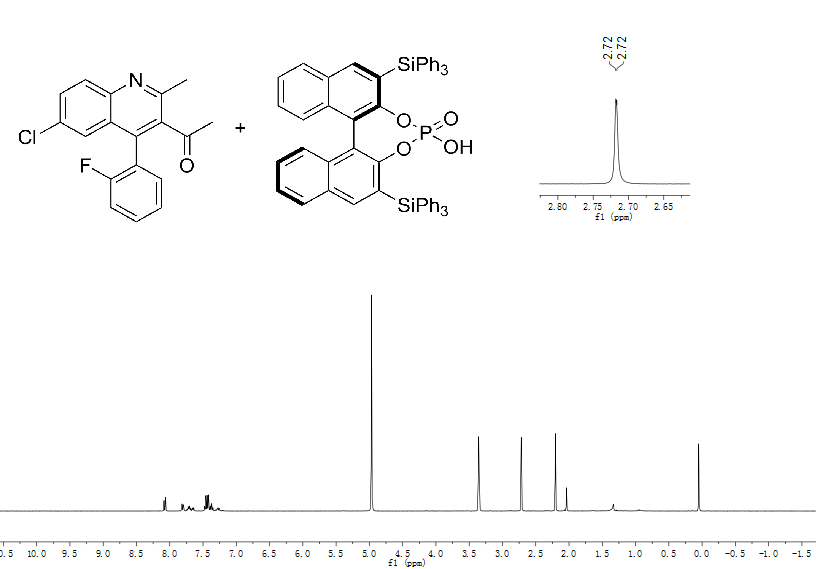


**5.8. ^1^H NMR (400 MHz, CD_3_OD) of (*R*)-C7 and guest 1a.**


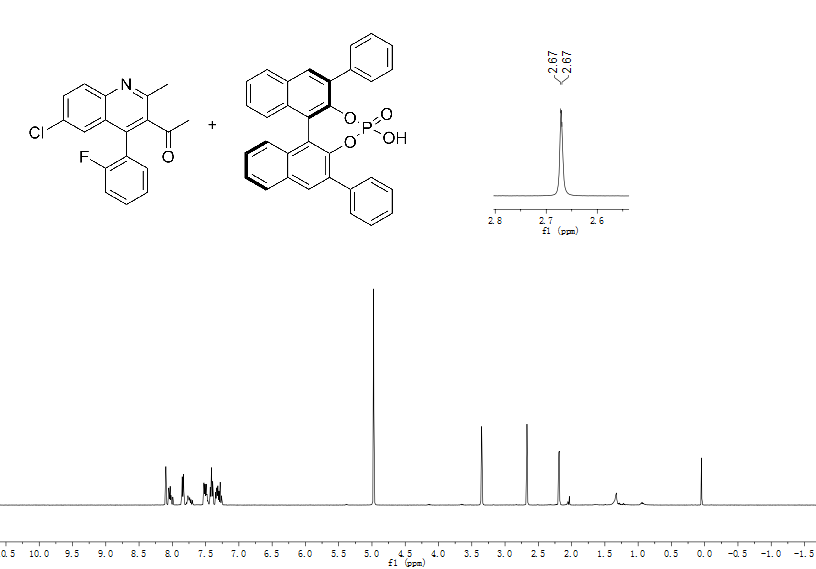


**5.9. ^1^H NMR (400 MHz, CD_3_OD) of (*R*)-C8 and guest 1a.**


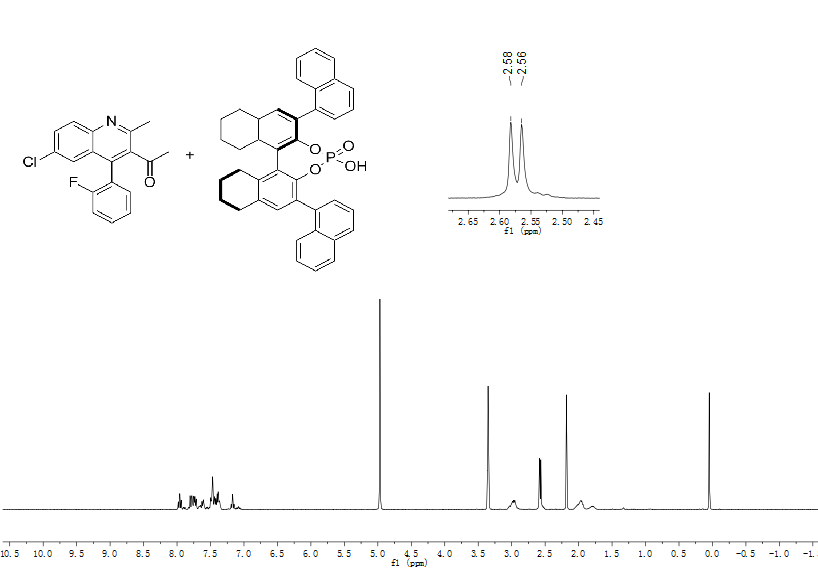


**5.10. ^1^H NMR (400 MHz, CD_3_OD) of (*R*)-C9 and guest 1a.**


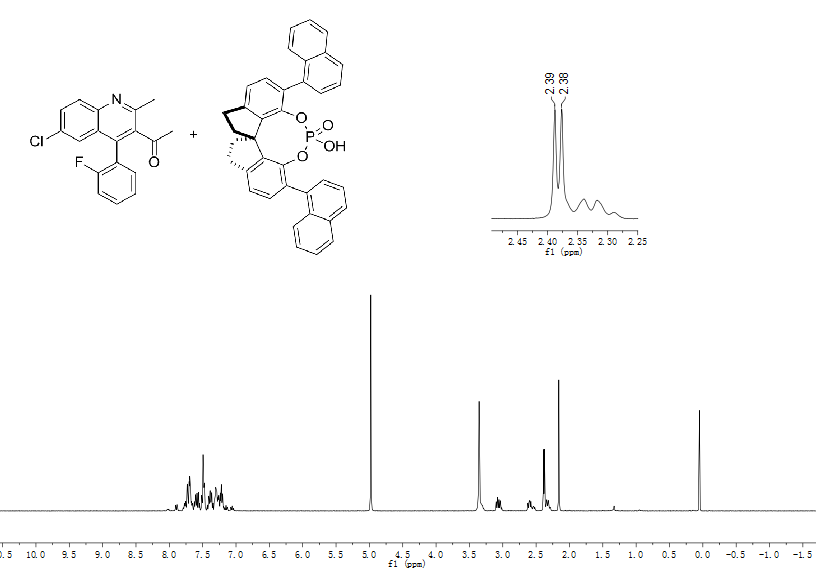


**5.11. ^1^H NMR (400 MHz, CDCl_3_) of (*R*)-C1 and guest 1a.**


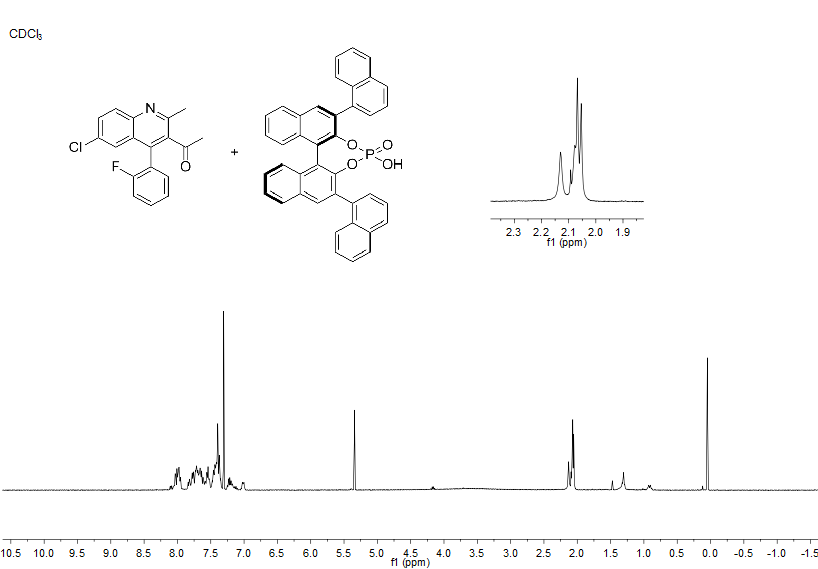


**5.12. ^1^H NMR (400 MHz, DMSO-D_6_) of (*R*)-C1 and guest 1a.**


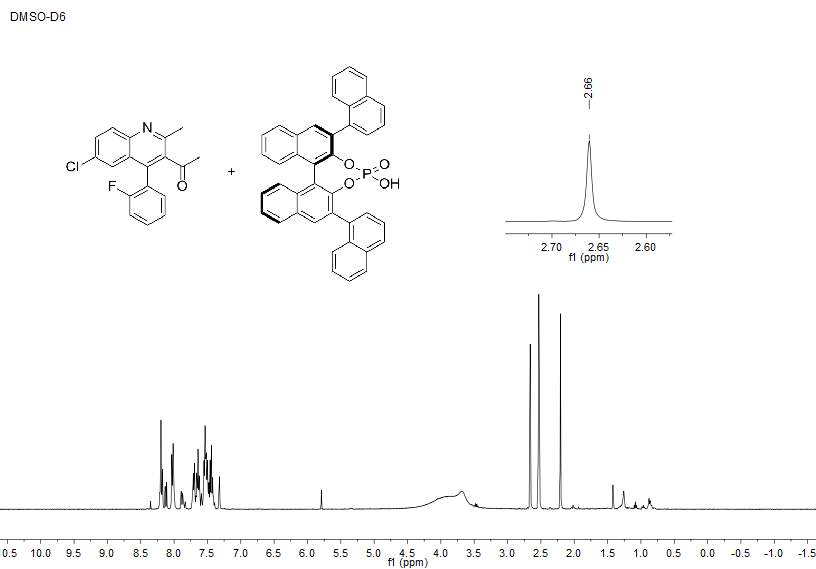


**5.13. ^1^H NMR (400 MHz,** **CD_3_CN) of (*R*)-C1 and guest 1a.**


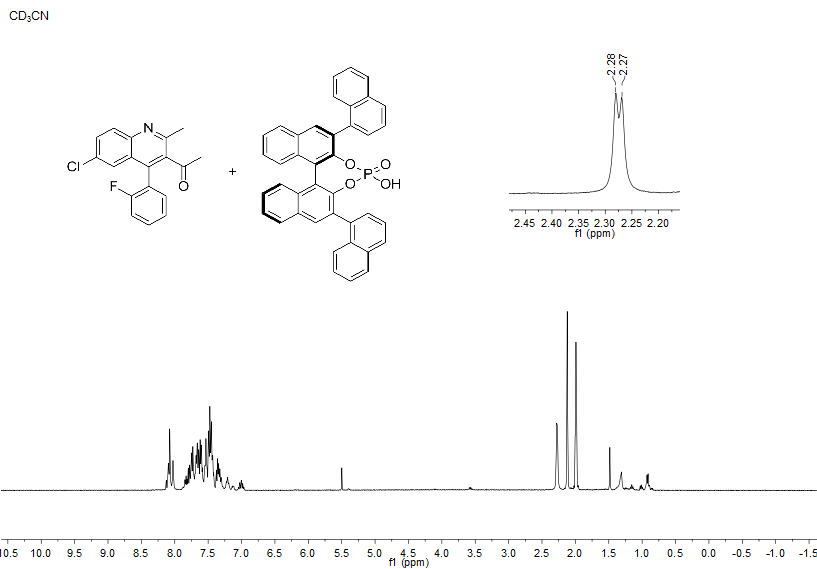


**5.14. ^1^H NMR (400 MHz, Acetone-D_6_) of (*R*)-C1 and guest 1a.**


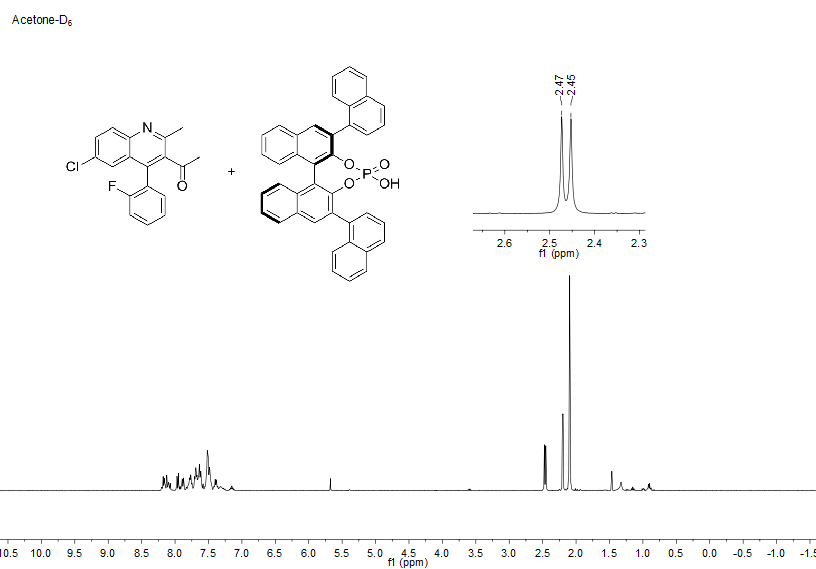


**5.15. ^1^H NMR (400 MHz, CD_3_OD+CDCl_3_) of (*R*)-C1 and guest 1a.**


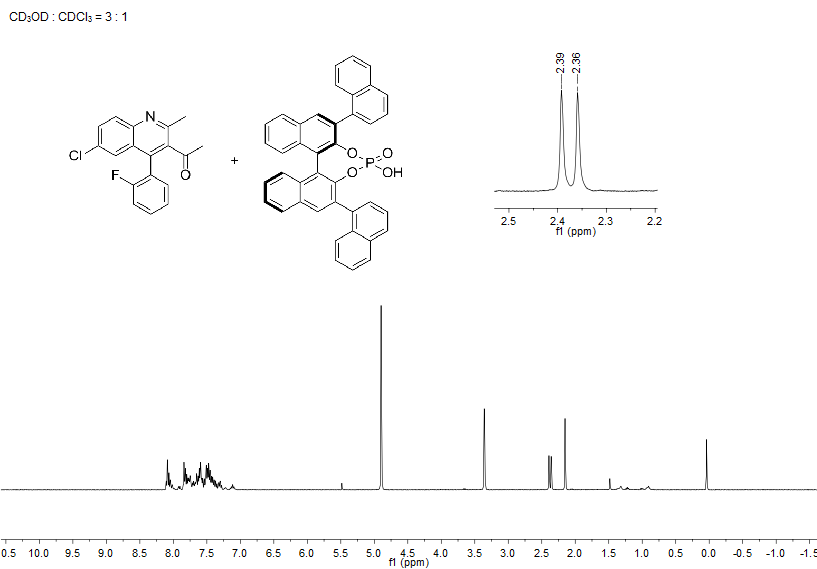


**5.16. ^1^H NMR (500 MHz, C_6_D_6_) of (*R*)-C1 and guest 1a.**


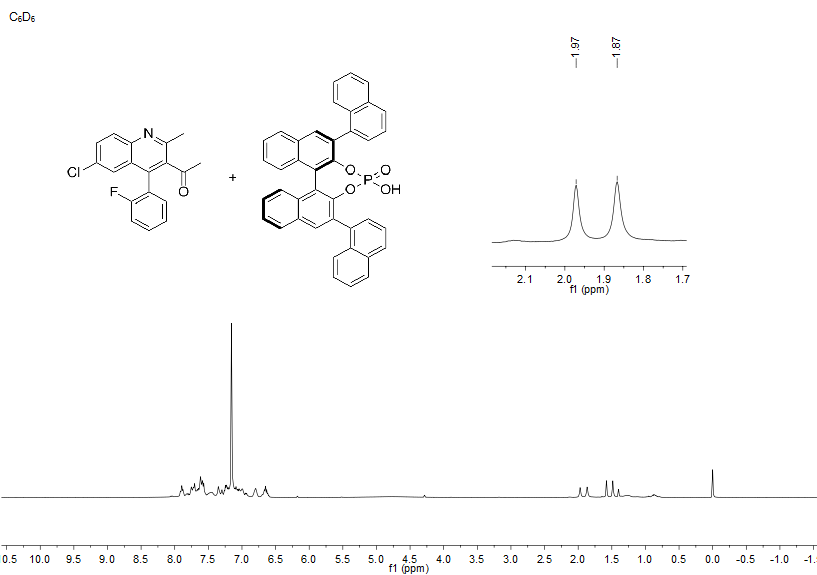


**5.17. ^1^H NMR (500 MHz, DMF-D_7_) of (*R*)-C1 and guest 1a.**


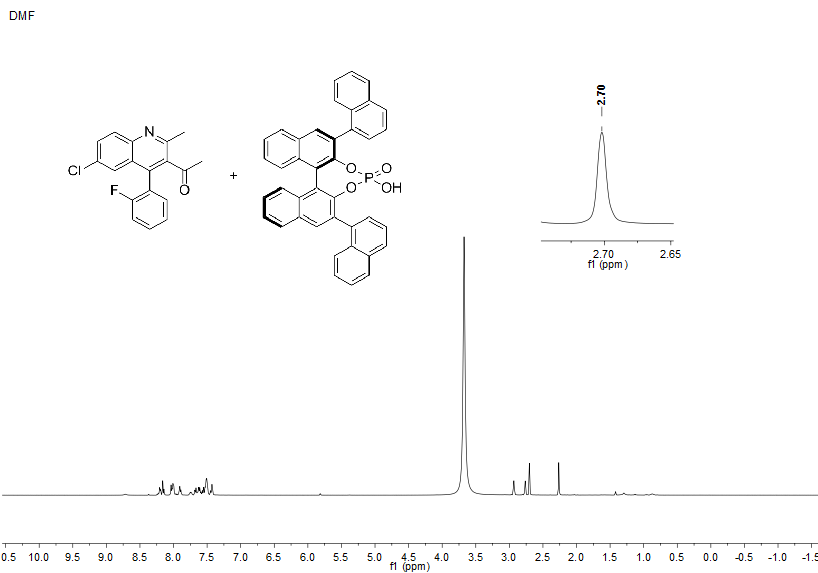


**5.18. ^1^H NMR (400 MHz, CD_3_OD) of (*R*)-C1 (0.005 mmol, 50% equiv.) and guest 1a (0.01 mmol, 1.0 equiv.).**


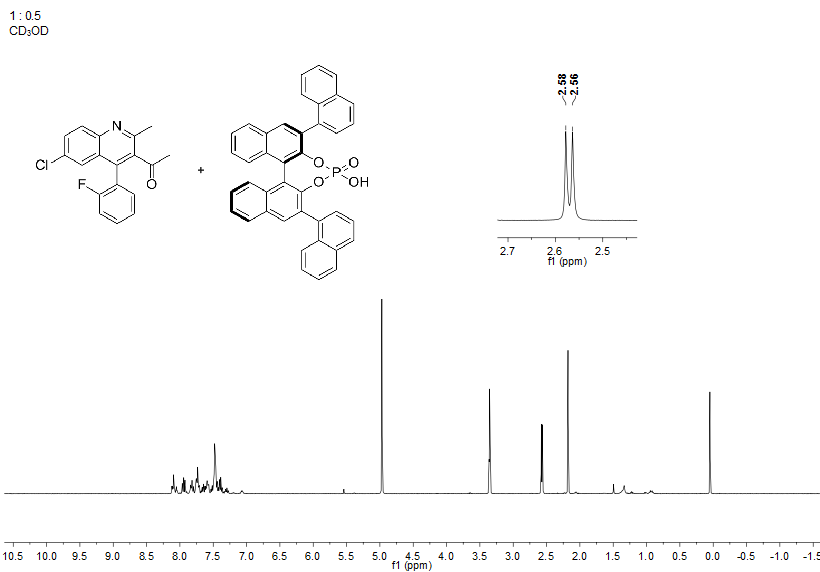


**5.19. ^1^H NMR (400 MHz, CD_3_OD) of (*R*)-C1 (0.02 mmol, 2.0 equiv.) and guest 1a (0.01 mmol, 1.0 equiv.).**


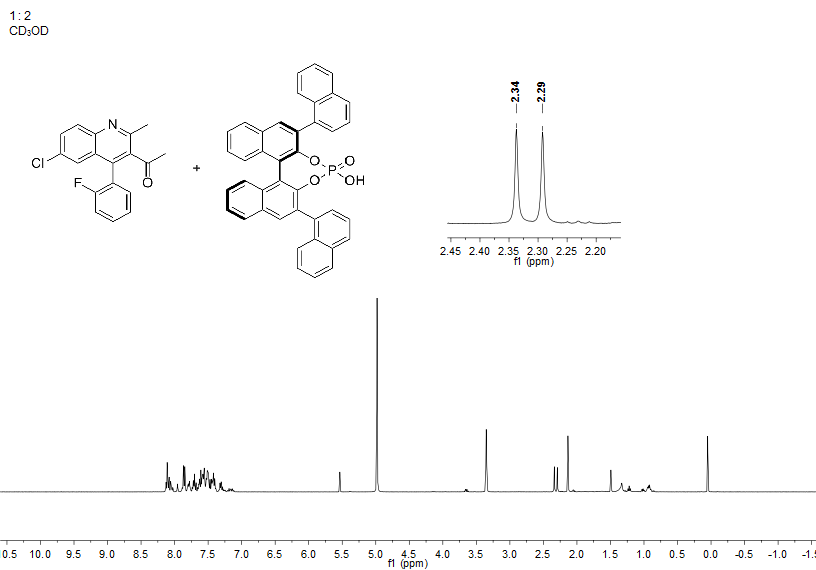


**6.** **^1^H NMR spectra of (R)-C1 (0.01 mmol, 1.0 equiv.) and racemic Aryl Quinolinones 1b-1s (0.01 mmol, 1.0 equiv.)**

**6.1. ^1^H NMR (400 MHz, C_6_D_6_) of racemic guest 1b.**


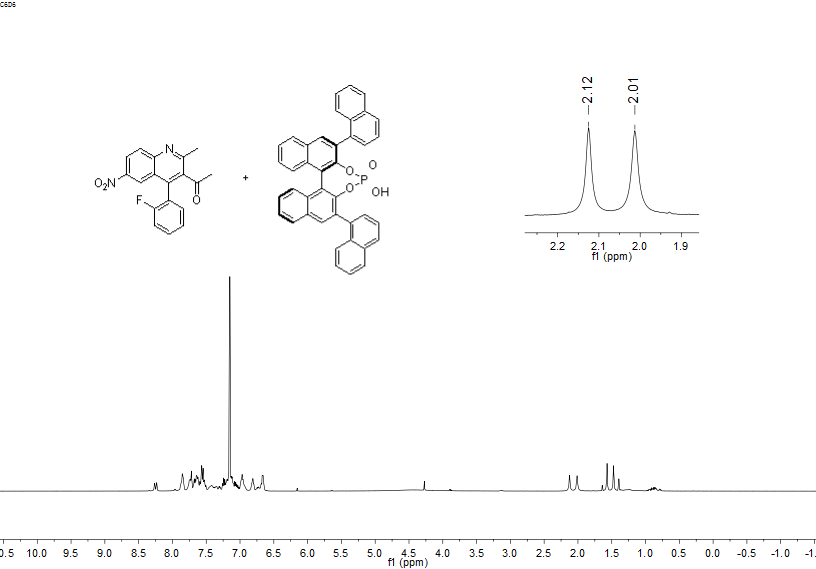


**6.2. ^1^H NMR (400 MHz, 0.5ml CD_3_OD + 0.1ml CDCl_3_) of racemic guest 1c.**


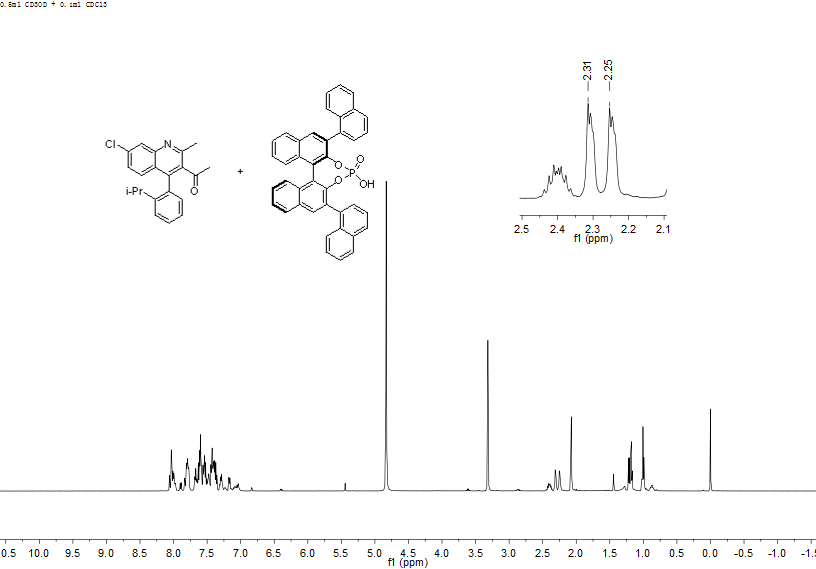


**6.3. ^1^H NMR (400 MHz,** **C_6_D_6_) of racemic guest 1d.**


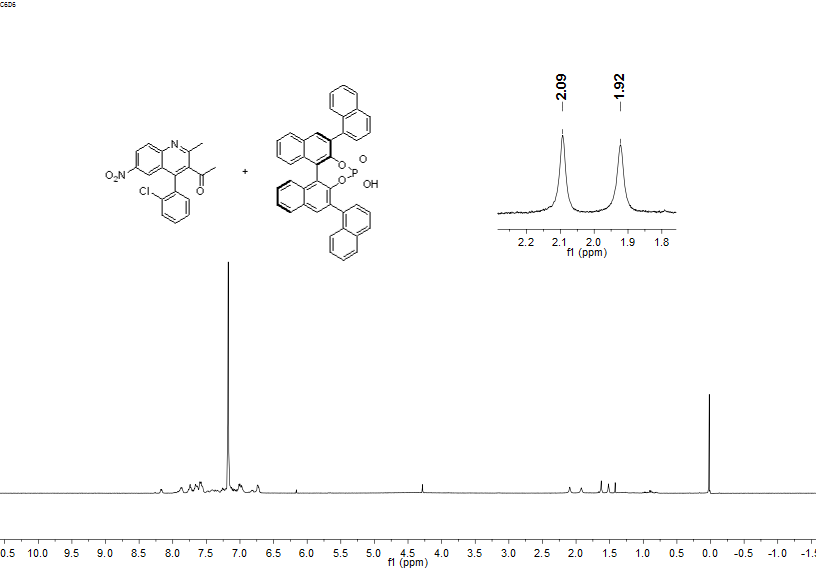


**6.4. ^1^H NMR (****400 MHz,** **0.5ml CD_3_OD + 0.1ml CDCl_3_) of racemic guest 1e.**


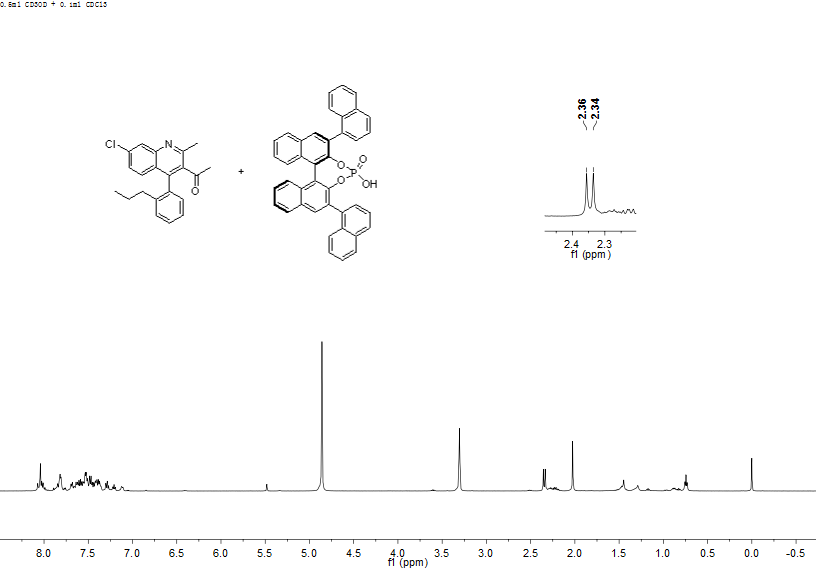


**6.5. ^1^H NMR (500 MHz, 0.5ml CD_3_OD + 0.1ml CDCl_3_) of racemic guest 1f.**


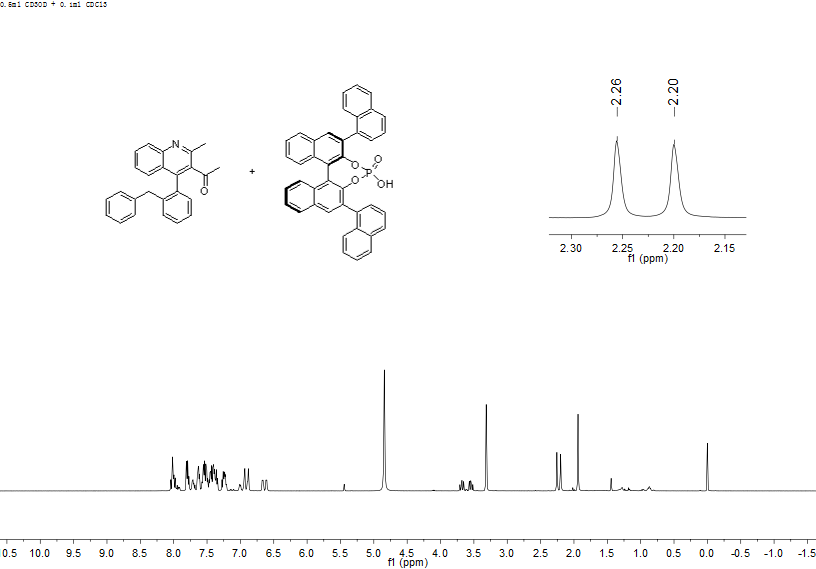


**6.6. ^1^H NMR (400 MHz, C_6_D_6_) of racemic guest 1g.**


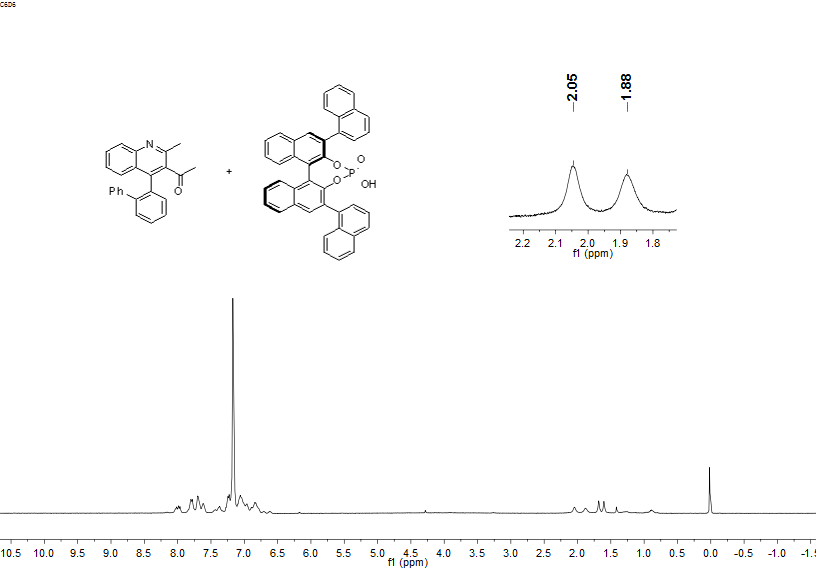


**6.7. ^1^H NMR (500 MHz, 0.5ml CD_3_OD + 0.1ml CDCl_3_) of racemic guest 1h.**


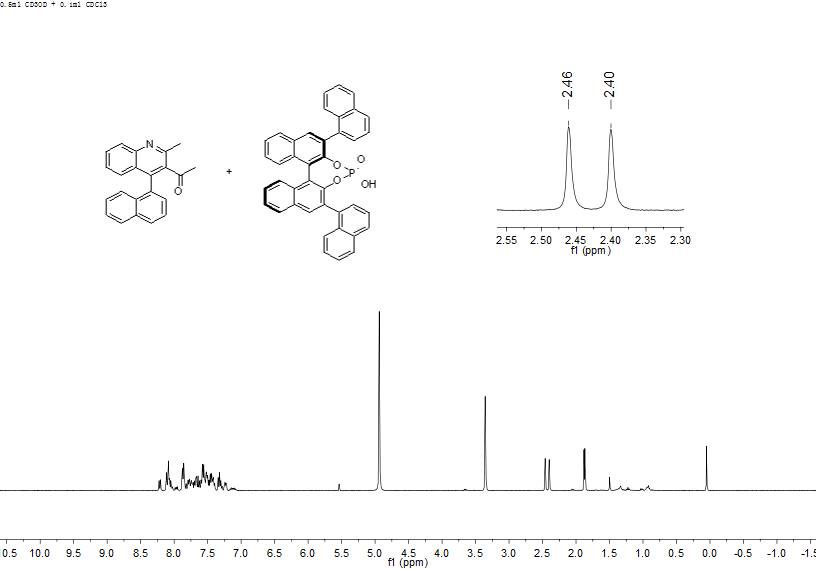


**6.8. ^1^H NMR (500 MHz, 0.5ml CD_3_OD + 0.1ml CDCl_3_, 2 equiv. of (R)-C1 was used) of racemic guest 1i.**


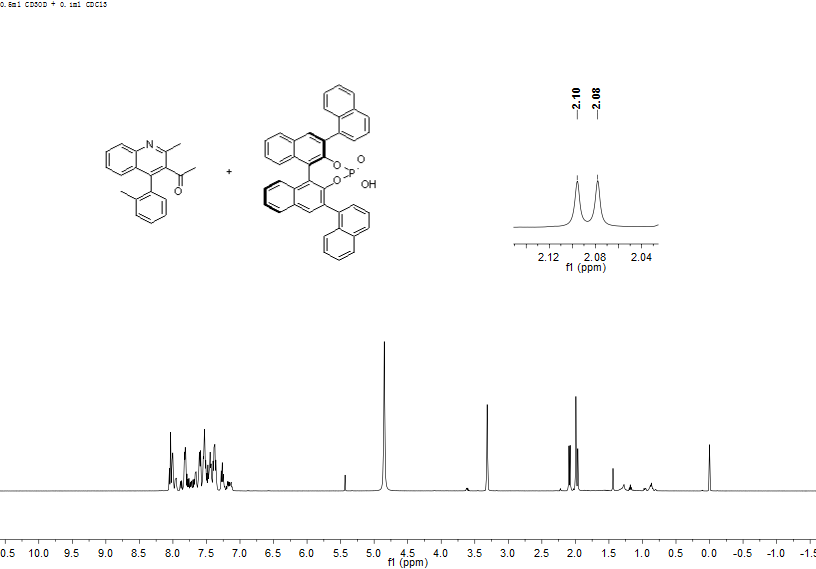


**6.9. ^1^H NMR (500 MHz, 0.5ml CD_3_OD + 0.1ml CDCl_3_) of racemic guest 1j.**


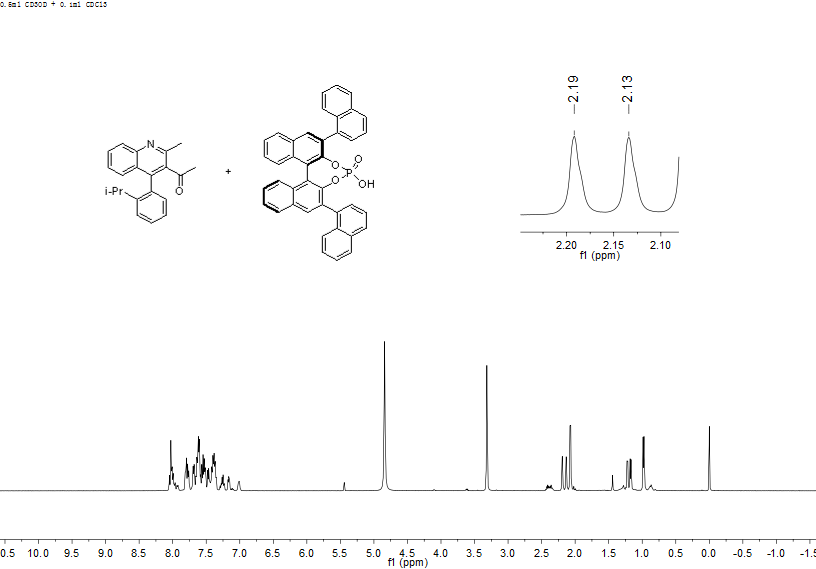


**6.10. ^1^H NMR (500 MHz, 0.5ml CD_3_OD + 0.1ml CDCl_3_) of racemic guest 1k.**


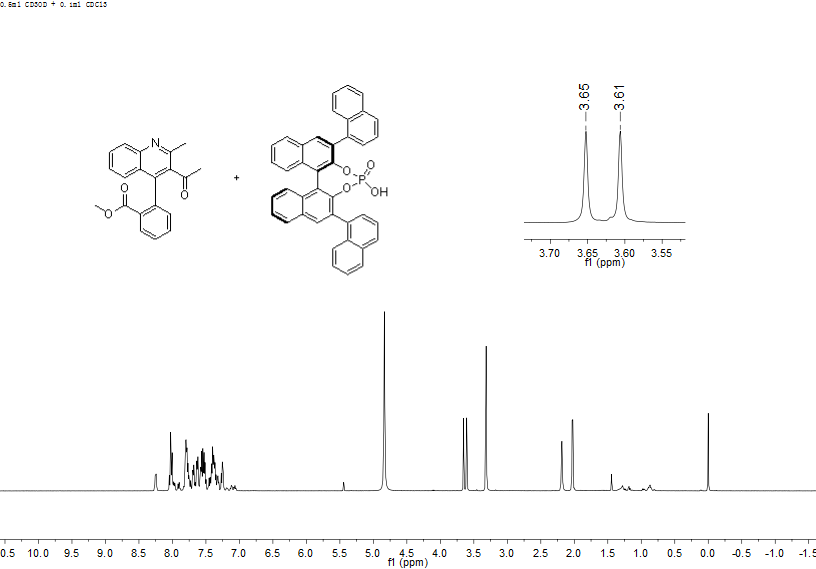


**6.11. ^1^H NMR (500 MHz, C_6_D_6_) of racemic guest 1l.**


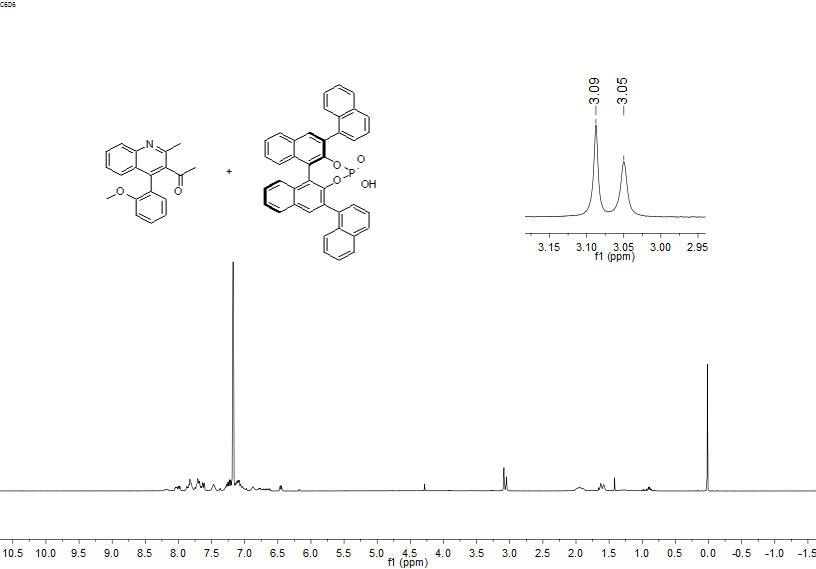


**6.12. ^1^H NMR (500 MHz, 0.5ml CD_3_OD + 0.1ml CDCl_3_) of racemic guest 1m.**


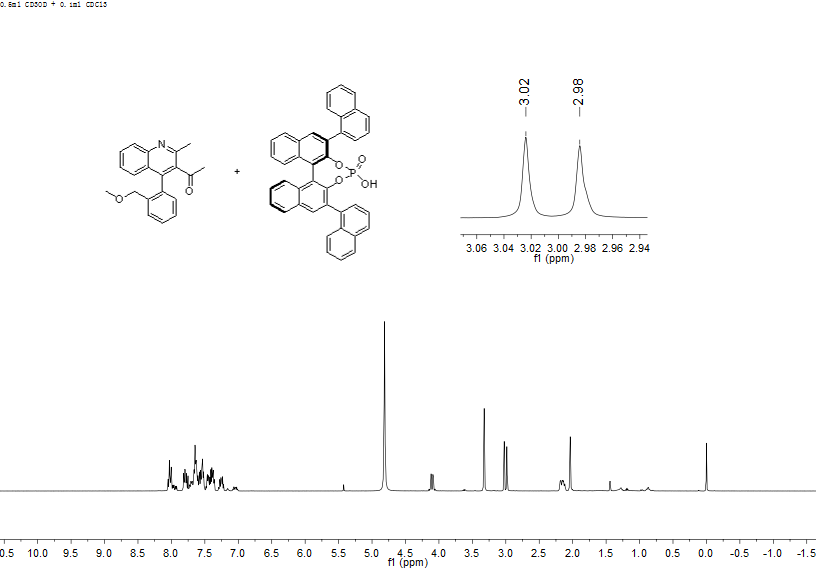


**6.13. ^1^H NMR (500 MHz, 0.5ml CD_3_OD + 0.1ml CDCl_3_) of racemic guest 1n.**


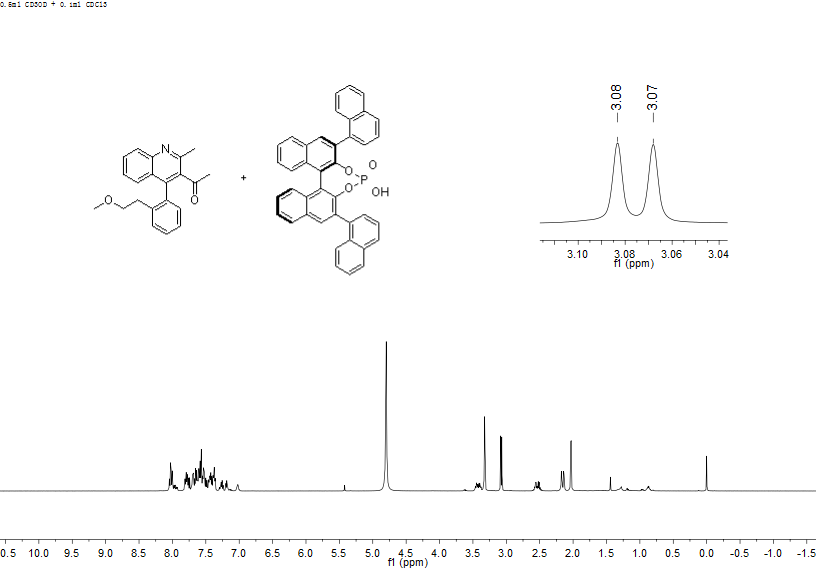


**6.14. ^1^H NMR (500 MHz, 0.5ml CD_3_OD + 0.1ml CDCl_3_) of racemic guest 1o.**


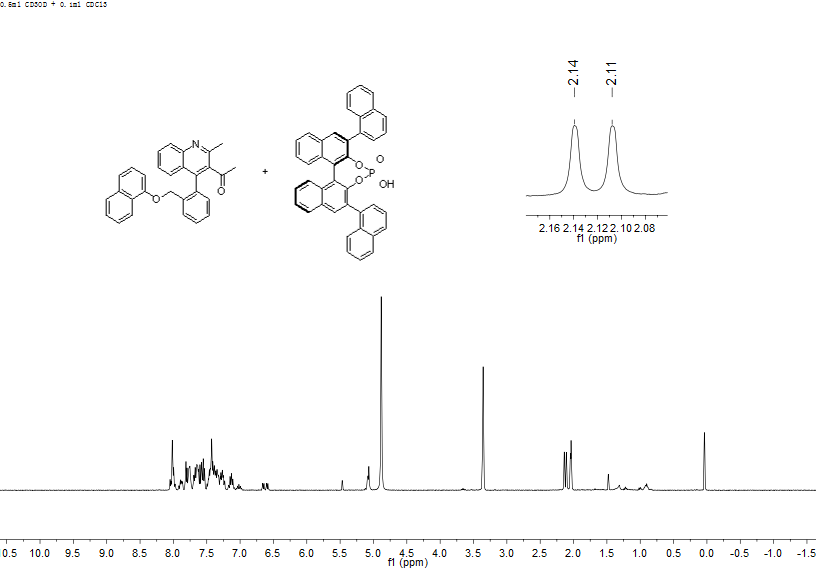


**6.15. ^1^H NMR (500 MHz, 0.5ml CD_3_OD + 0.1ml CDCl_3_) of racemic guest 1p.**


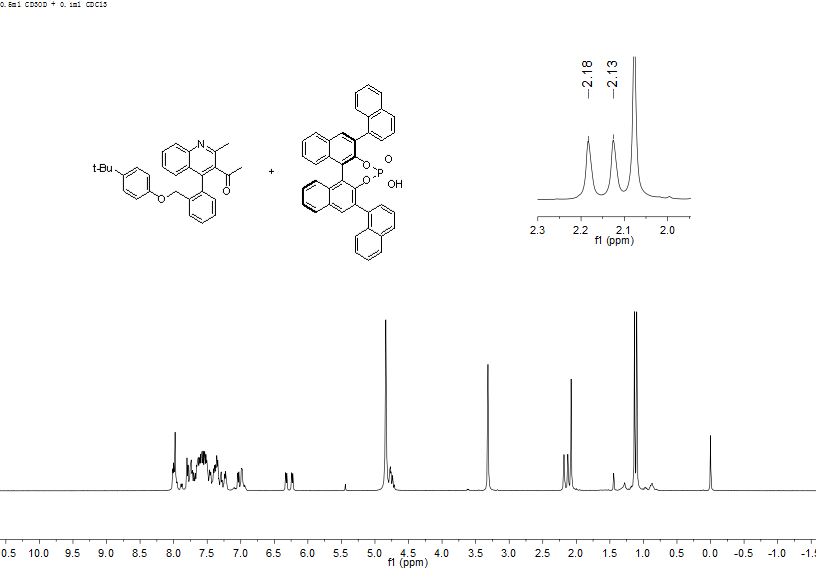


**6.16. ^1^H NMR (400 MHz, 0.5ml CD_3_OD + 0.1ml CDCl_3_) of racemic guest 1q.**


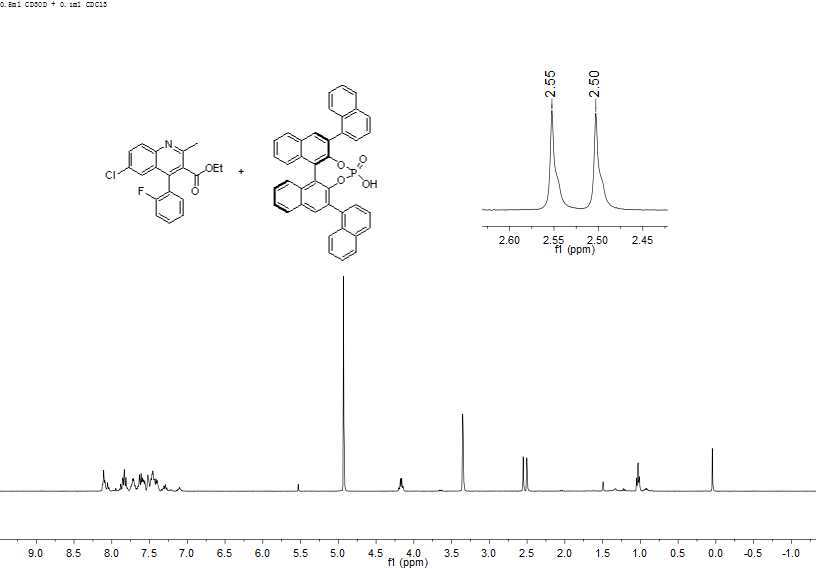


**6.17. ^1^H NMR (400 MHz, 0.5ml CD_3_OD + 0.1ml CDCl_3_) of racemic guest 1r.**


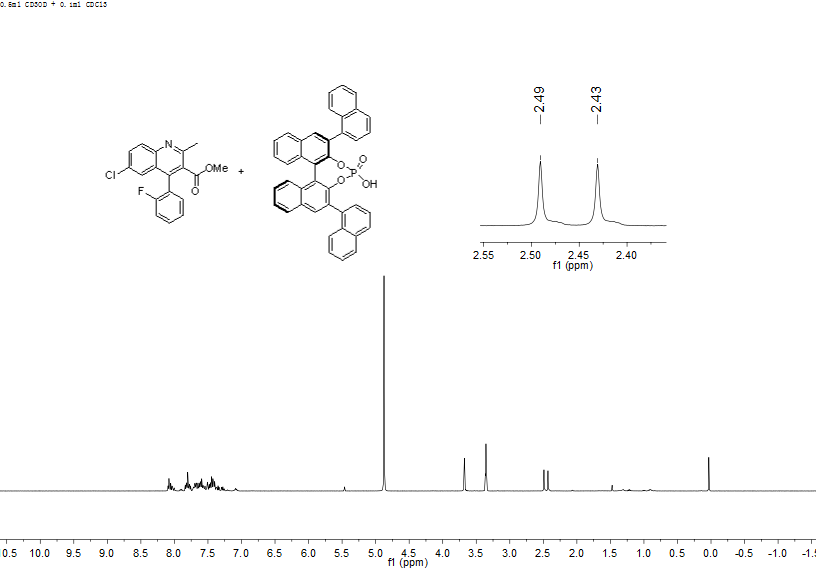


**6.18. ^1^H NMR (500 MHz, C_6_D_6_) of racemic guest 1s.**


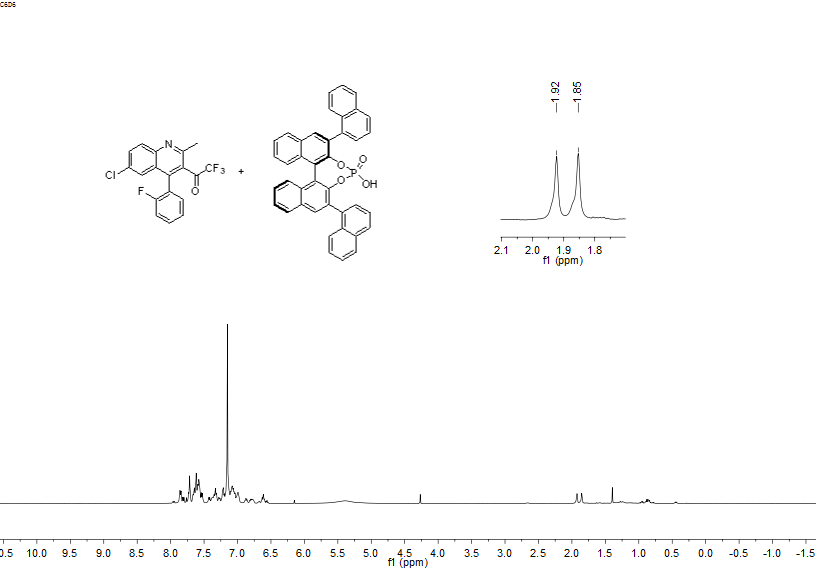


**7. ^1^H NMR spectra/HPLC data of** **(*R*)-C1 (0.01 mmol, 1.0 equiv.) and 1j (0.01 mmol, 1.0 equiv.) with different optical purities.**

**^1^H NMR** **(500 MHz, 0.5ml CD_3_OD + 0.1ml CDCl_3_) of (*R*)-C1 and guest 1j (Sample 1, ee 91.031%).**

**
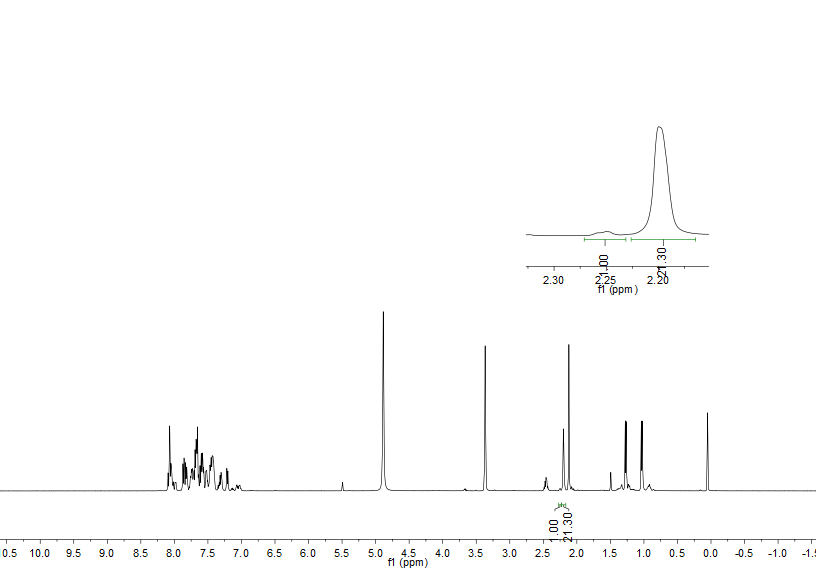
**

**HPLC of guest 1j'** **Daicel Chiralpak AD-H, hexane/iso-propanol= 90/10, flow rate 1.0 mL/min, λ = 254 nm, 25°С(Sample 1, ee 90.402%).**


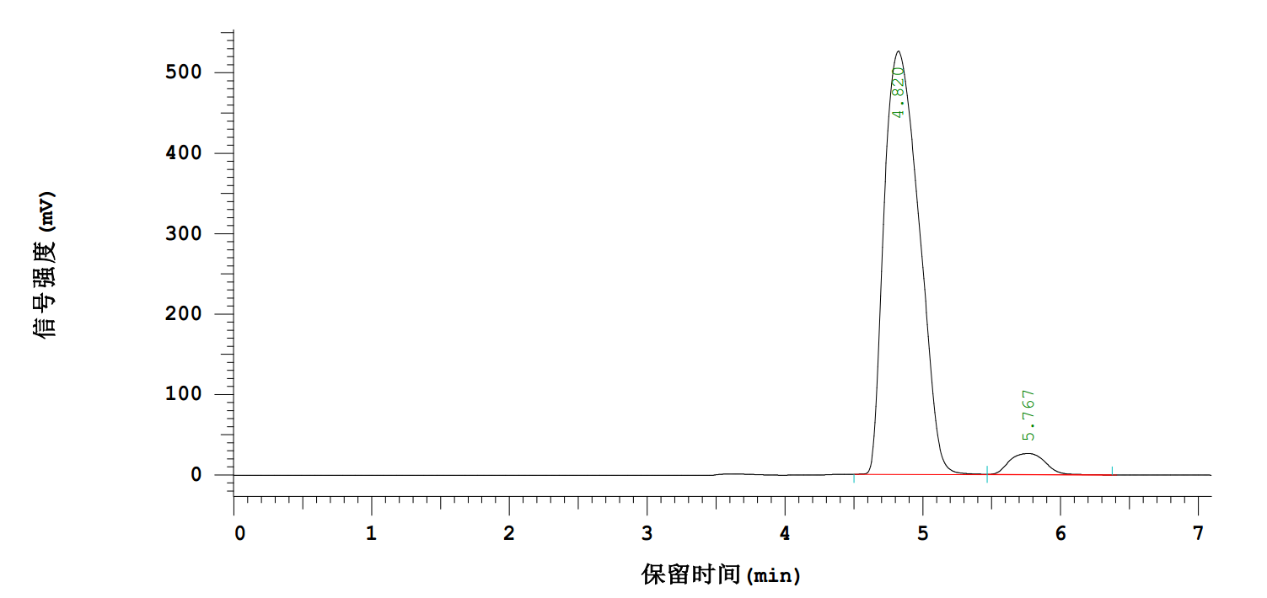


| Peak | Ret. Time | Area% |
| --- | --- | --- |
| A | 4.820 | 95.201 |
| B | 5.767 | 4.799 |

**^1^H NMR (500 MHz, 0.5ml CD_3_OD + 0.1ml CDCl_3_) of (*R*)-C1 and guest 1j (Sample 2, ee 80.178%).**

**
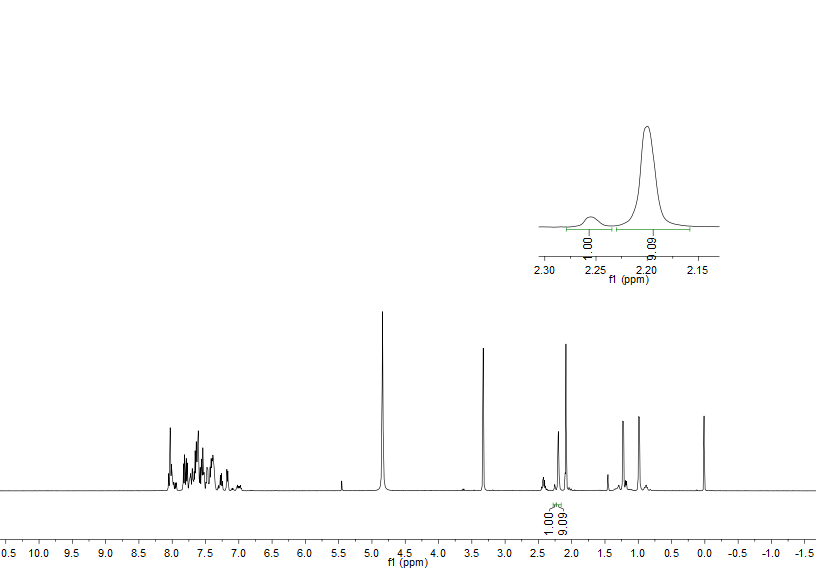
**

**HPLC of guest 1j' Daicel Chiralpak AD-H, hexane/iso-propanol= 90/10, flow rate 1.0 mL/min, λ = 254 nm, 25°С(Sample 2, ee 80.200%).**


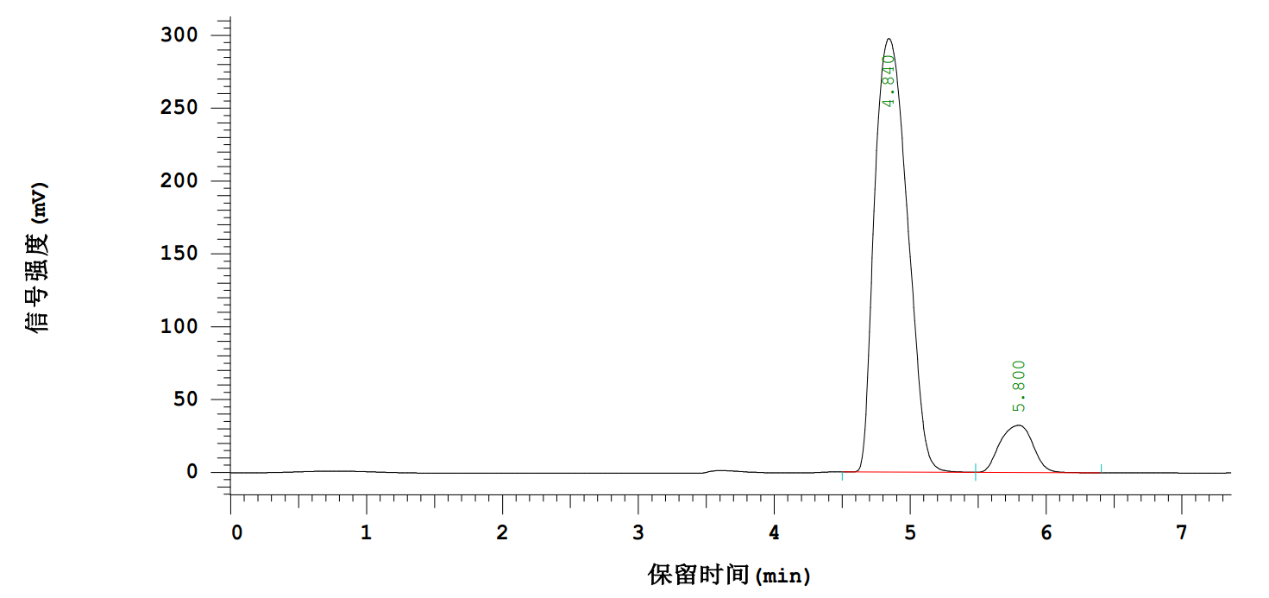


| Peak | Ret. Time | Area% |
| --- | --- | --- |
| A | 4.840 | 90.100 |
| B | 5.800 | 9.900 |

**^1^H NMR (500 MHz, 0.5ml CD_3_OD + 0.1ml CDCl_3_) of (*R*)-C1 and guest 1j (Sample 3, ee 69.088%).**

**
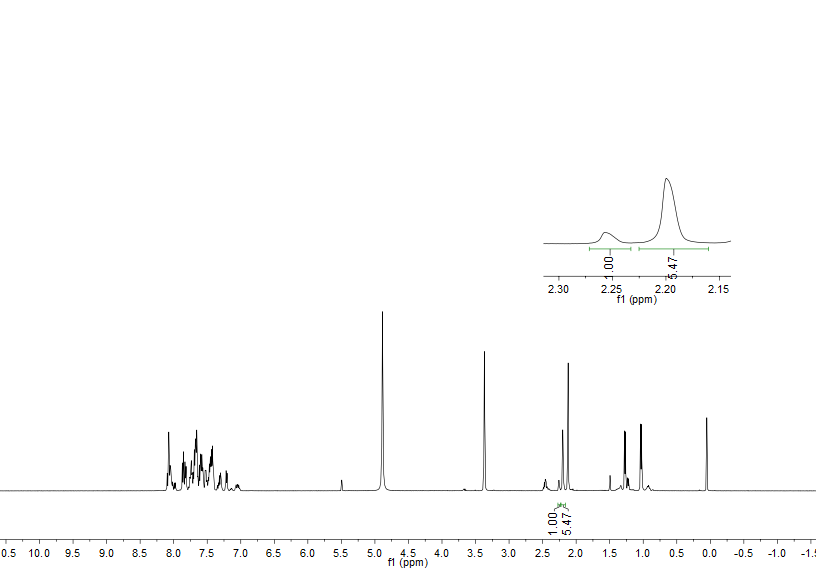
**

**HPLC of guest 1j' Daicel Chiralpak AD-H, hexane/iso-propanol= 90/10, flow rate 1.0 mL/min, λ = 254 nm, 25°С(Sample 3, ee 69.570%).**


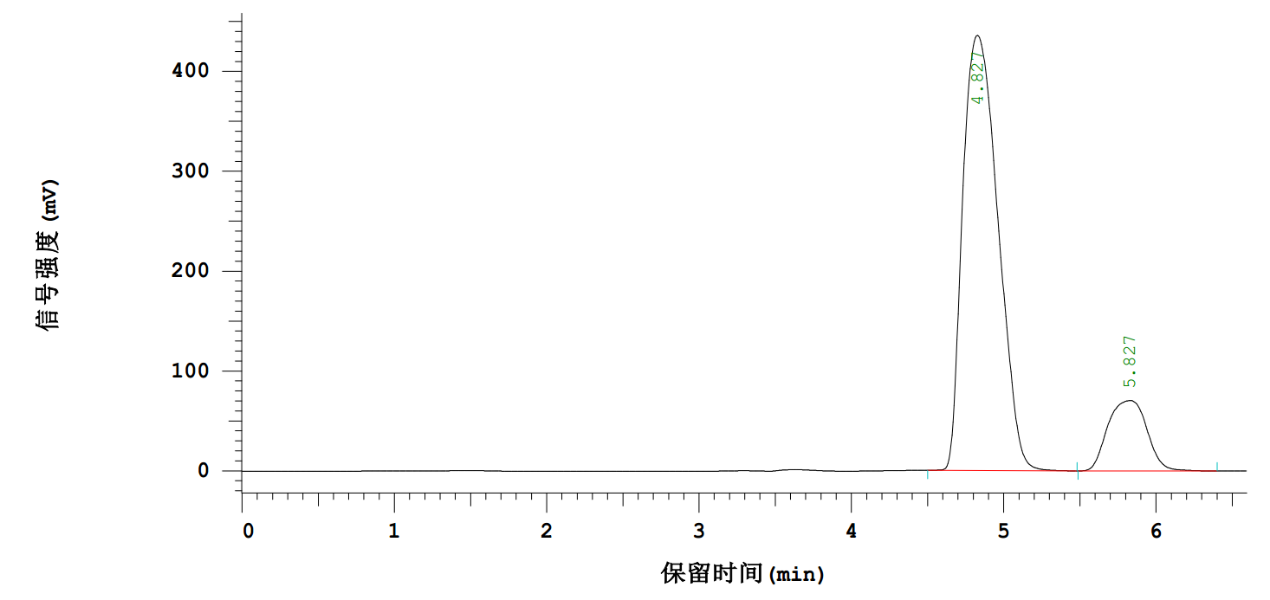


| Peak | Ret. Time | Area% |
| --- | --- | --- |
| A | 4.827 | 84.785 |
| B | 5.827 | 15.215 |

**^1^H NMR (500 MHz, 0.5ml CD_3_OD + 0.1ml CDCl_3_) of (*R*)-C1 and guest 1j (Sample 4, ee 60.080%).**

**
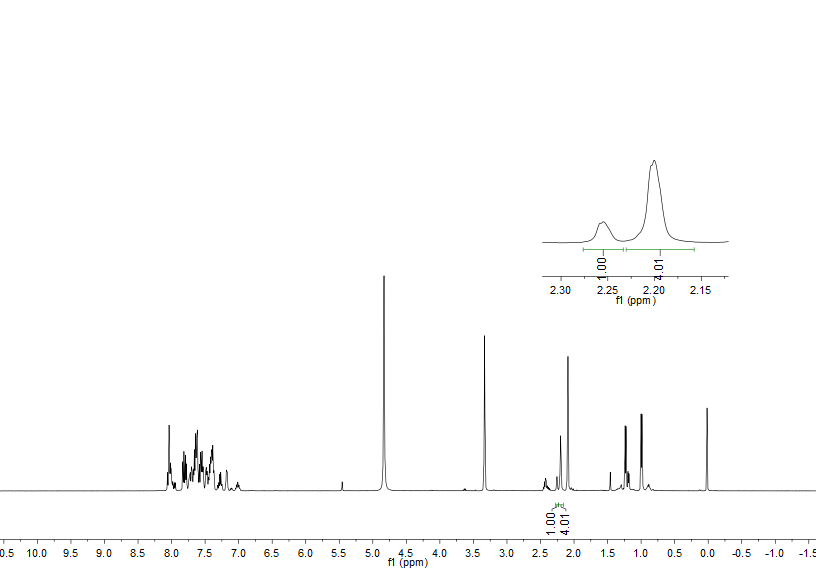
**

**HPLC of guest 1j' Daicel Chiralpak AD-H, hexane/iso-propanol= 90/10, flow rate 1.0 mL/min, λ = 254 nm, 25°С(Sample 4, ee 60.598%).**


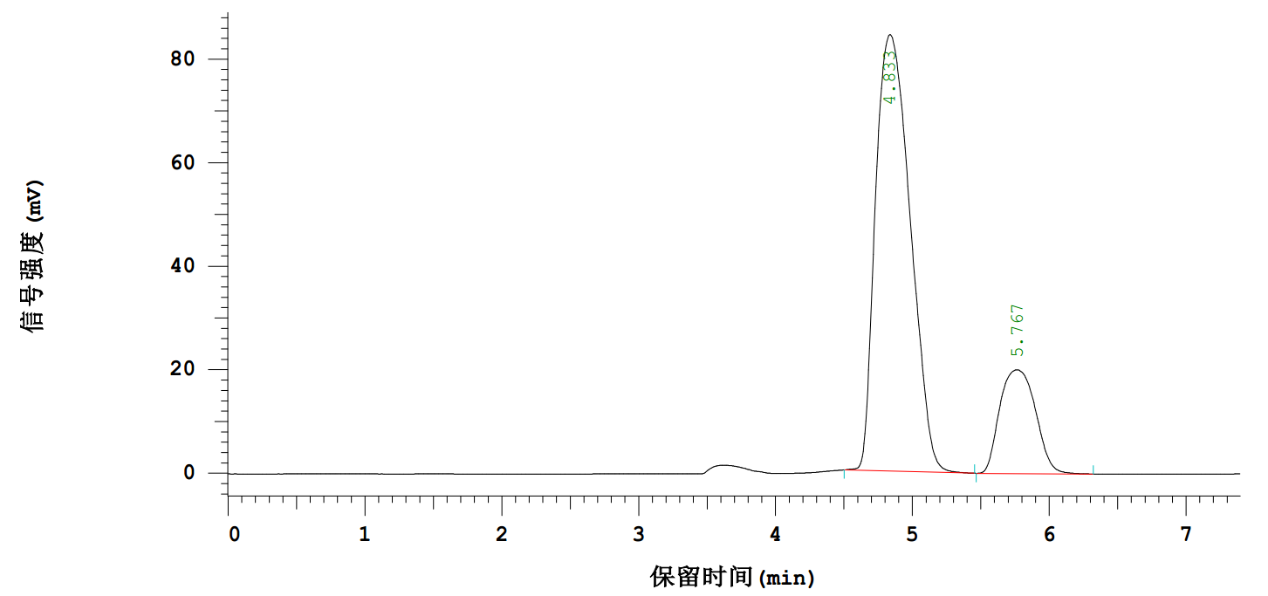


| Peak | Ret. Time | Area% |
| --- | --- | --- |
| A | 4.833 | 80.299 |
| B | 5.767 | 19.701 |

**^1^H NMR (500 MHz, 0.5ml CD_3_OD + 0.1ml CDCl_3_) of (*R*)-C1 and guest 1j (Sample 5, ee 40.828/%).**

**
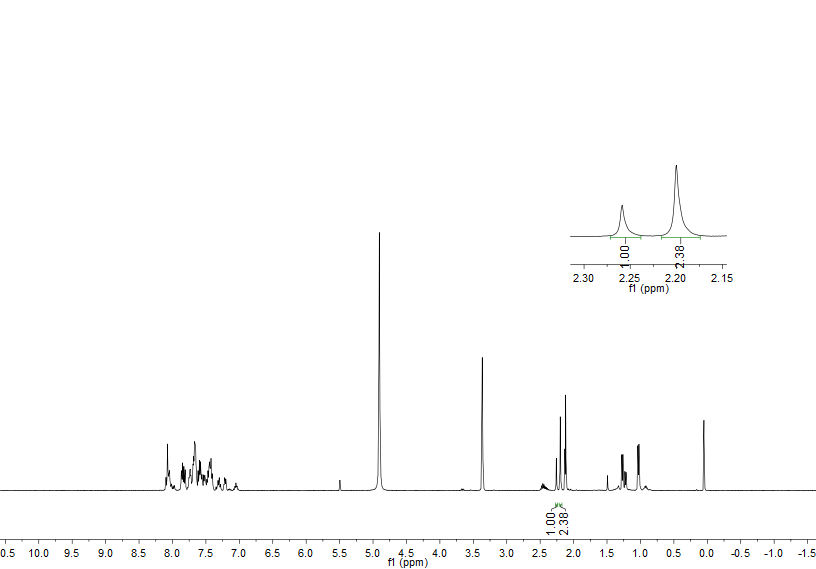
**

**HPLC of guest 1j' Daicel Chiralpak AD-H, hexane/iso-propanol= 90/10, flow rate 1.0 mL/min, λ = 254 nm, 25°С(Sample 5, ee 41.202%).**


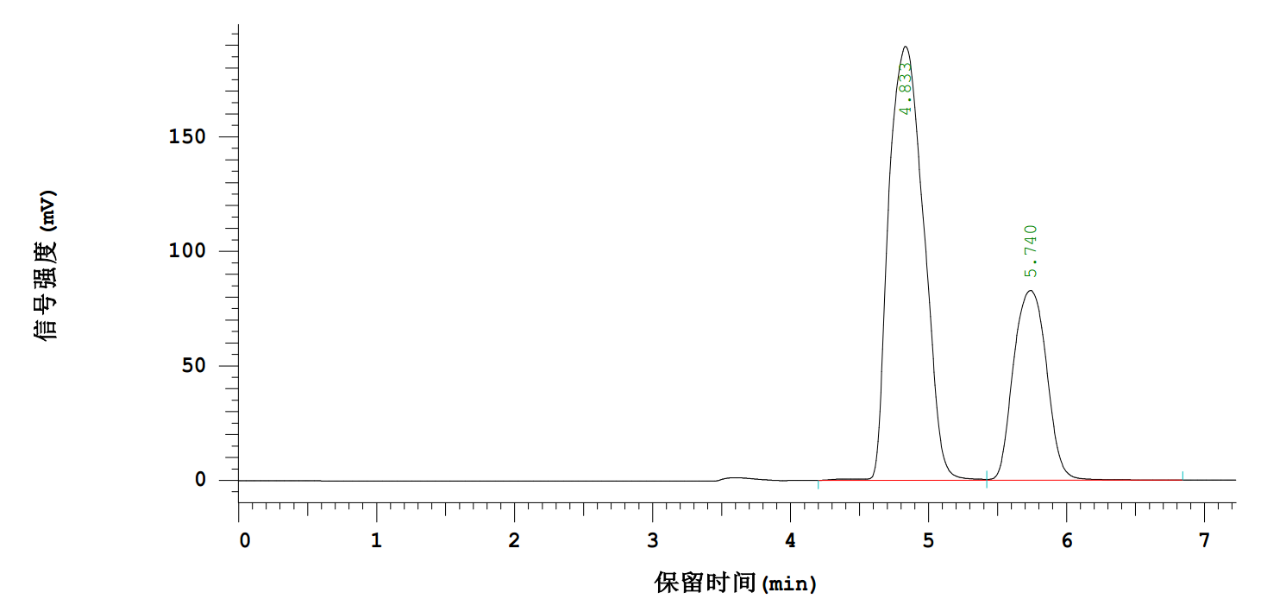


| Peak | Ret. Time | Area% |
| --- | --- | --- |
| A | 4.833 | 70.601 |
| B | 5.740 | 29.399 |

**^1^H NMR (500 MHz, 0.5ml CD_3_OD + 0.1ml CDCl_3_) of (*R*)-C1 and guest 1j(Sample 6, ee 27.798%).**

**
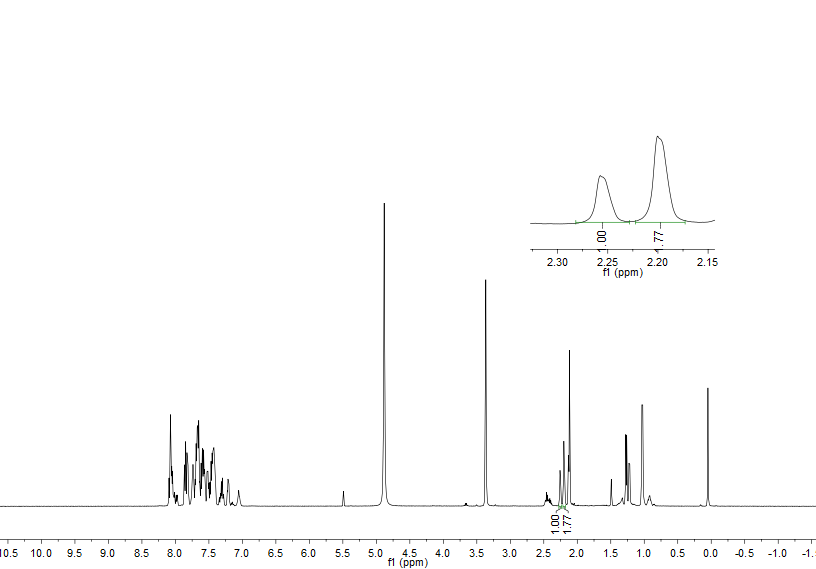
**

**HPLC of guest 1j' Daicel Chiralpak AD-H, hexane/iso-propanol= 90/10, flow rate 1.0 mL/min, λ = 254 nm, 25°С(Sample 6, ee 27.392%).**


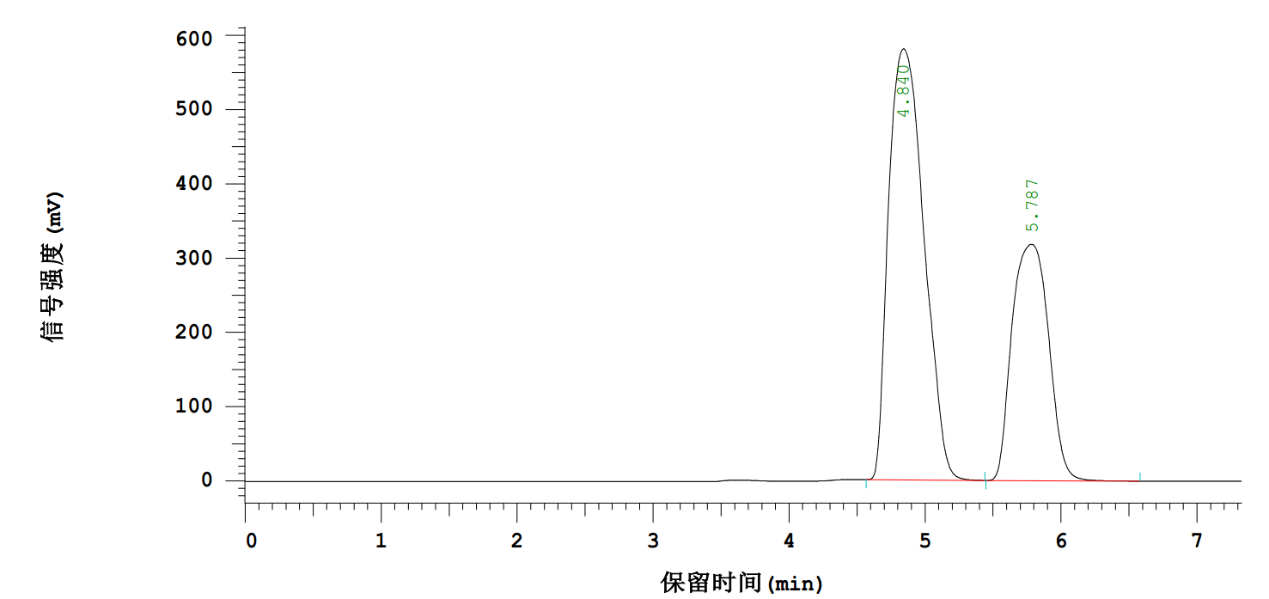


| Peak | Ret. Time | Area% |
| --- | --- | --- |
| A | 4.840 | 63.696 |
| B | 5.787 | 36.304 |

**^1^H NMR (500 MHz, 0.5ml CD_3_OD + 0.1ml CDCl_3_) of (*R*)-C1 and guest 1j (Sample 7, ee 17.012%).**

**
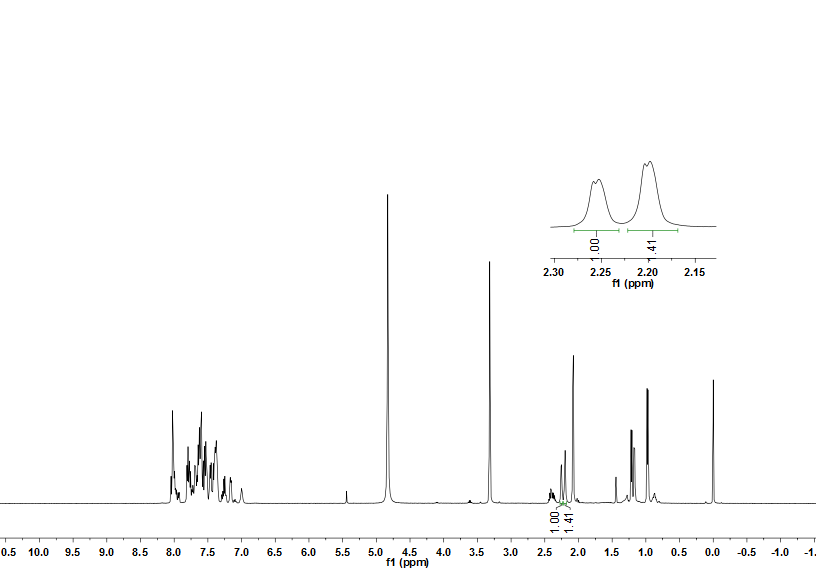
**

**HPLC of guest 1j' Daicel Chiralpak AD-H, hexane/iso-propanol= 90/10, flow rate 1.0 mL/min, λ = 254 nm, 25°С(Sample 7, ee 17.568%).**


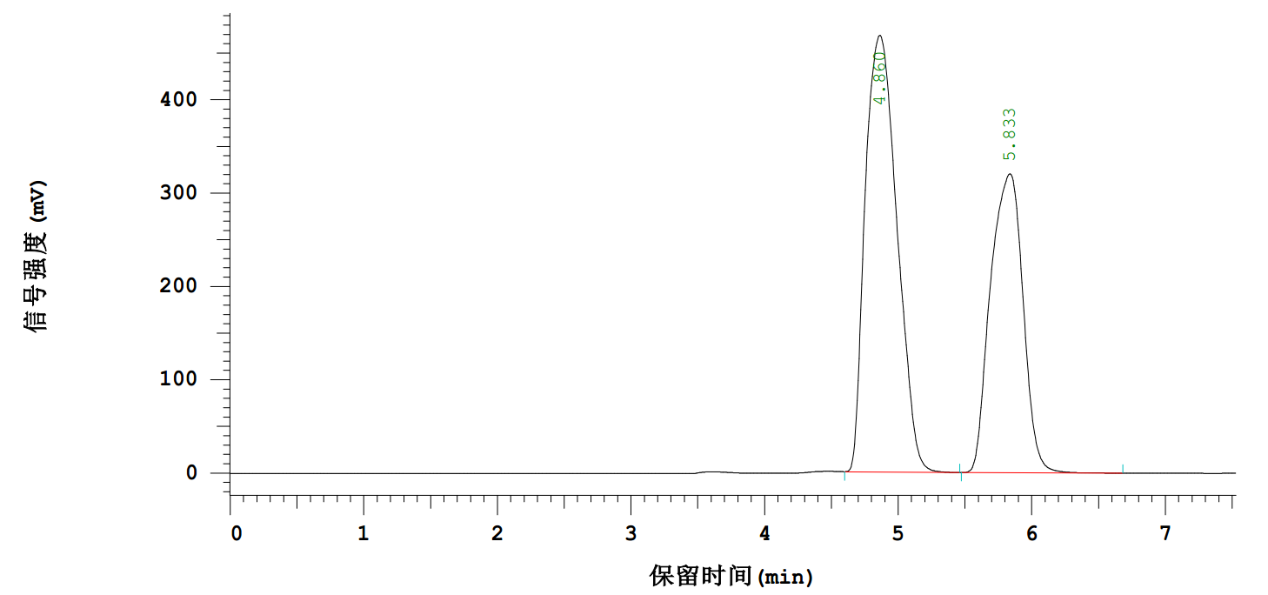


| Peak | Ret. Time | Area% |
| --- | --- | --- |
| A | 4.860 | 58.787 |
| B | 5.833 | 41.216 |

**^1^H NMR (500 MHz, 0.5ml CD_3_OD + 0.1ml CDCl_3_) of (*R*)-C1 and guest 1j (Sample 8, ee 0%).**

**
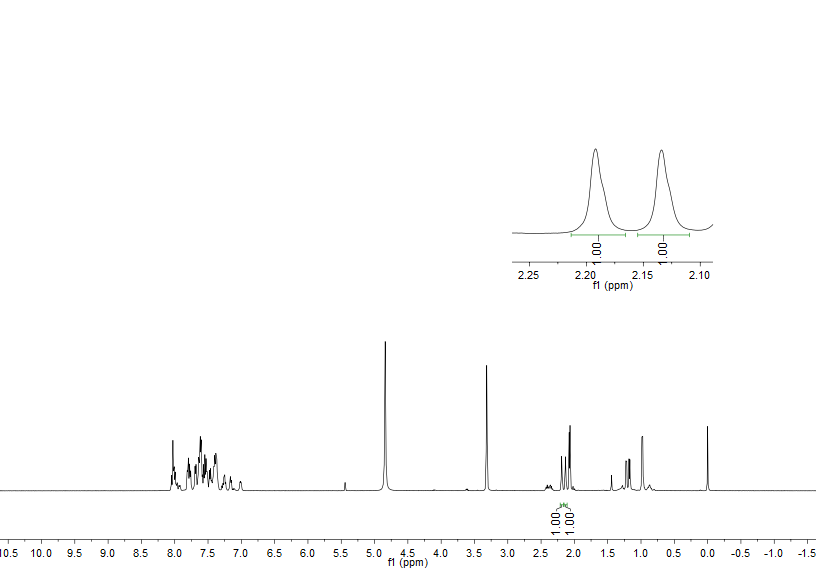
**

**HPLC of guest 1j' Daicel Chiralpak AD-H, hexane/iso-propanol= 90/10, flow rate 1.0 mL/min, λ = 254 nm, 25°С(Sample 8, ee 0.936%).**


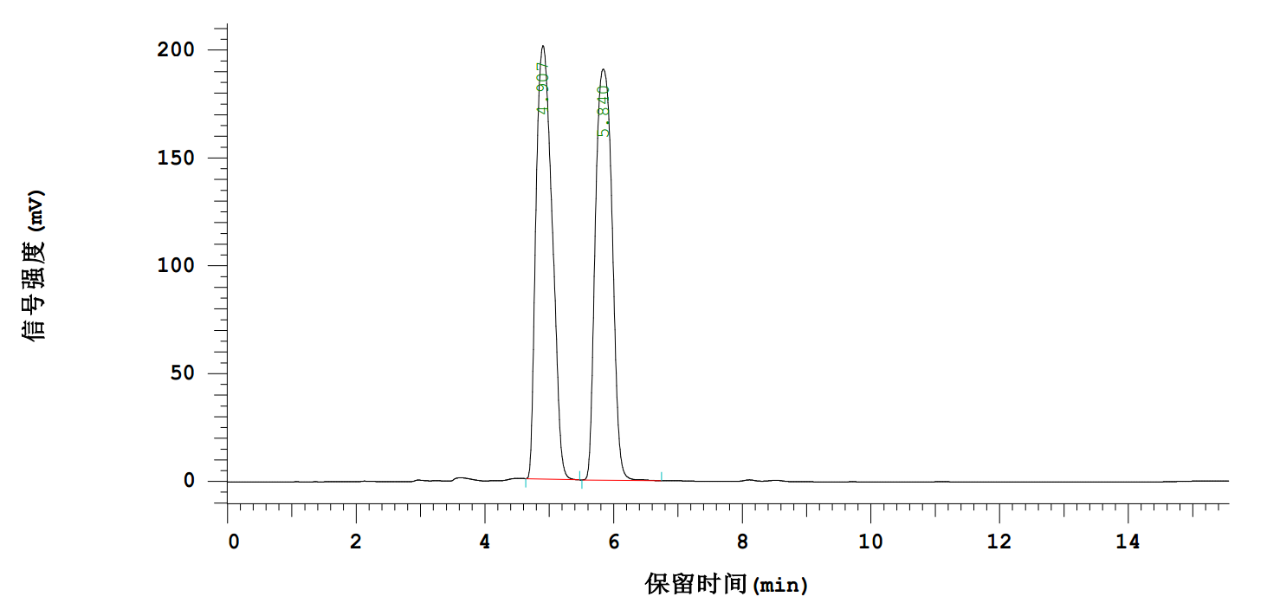


| Peak | Ret. Time | Area% |
| --- | --- | --- |
| A | 4.907 | 50.468 |
| B | 5.840 | 49.532 |

**8. Reference**

[1] Romanov-Michailidis, F.; Guénée, L.; Alexakis, A. *Org. Lett.*, **2013**, *15*, 5890.

[2] Tanji, Y.; Mitsutake, Y.; Fujihara, T.; Tsuji, Y. *Angew. Chem. Int. Ed.*, **2018**, 57, 10314.

[3] Cho, S. H.; Hartwig, J. F. *J. Am. Chem. Soc.*, **2013**, *135*, 8157.

[4] Evoniuk, C. J.; Gomes, G. P.; Hill, S. P.; Fujita, S.; Hanson, K.; Alabugin, I. V.;

*J. Am. Chem. Soc.*, **2017**, *139*, 16210.

[5] Fan, X.; Yu, L. Z.; Wei, Y.; Shi, M. *Org. Lett.*, **2017**, *19*, 4476.

[6] Ryabukhin, S. V.; Volochnyuk, D. M.; Plaskon, A. S.; Naumchik, V. S.; Tolmachev, A. A. *Synthesis.,* **2007**, *8*, 1214.

**9. ^1^H and ^13^C NMR Spectra of Aryl Quinolinones 1a-1s**

**1a**  ^1^H NMR (500 MHz, CDCl_3_)

**
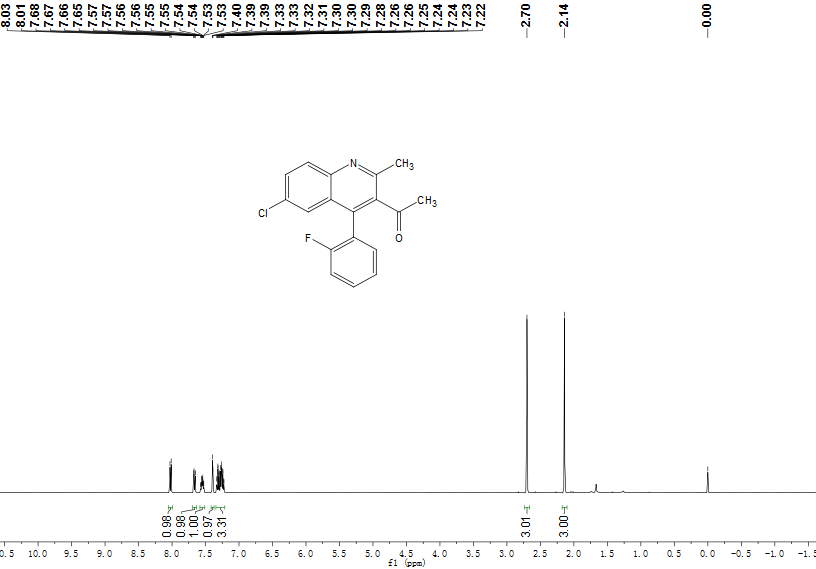
**

**1a** ^13^C NMR (126 MHz, CDCl_3_)


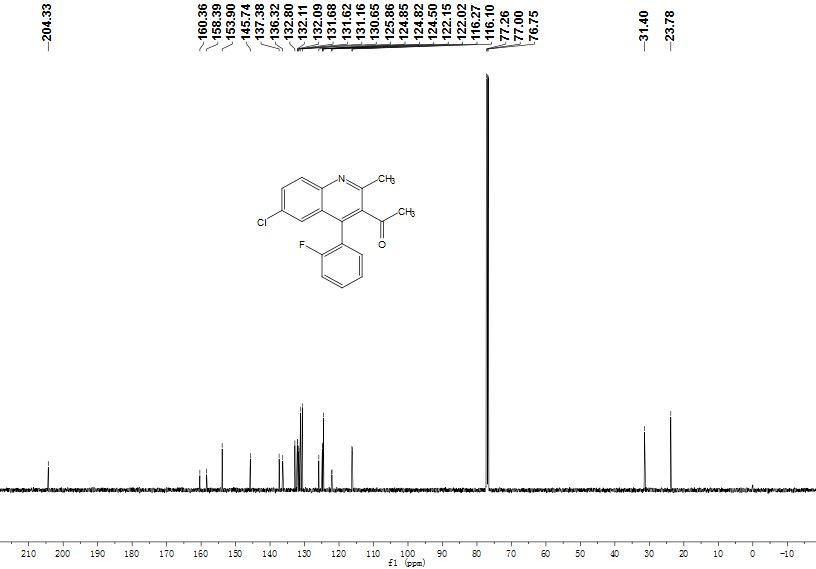


**1b**  ^1^H NMR (500 MHz, CDCl_3_)

**
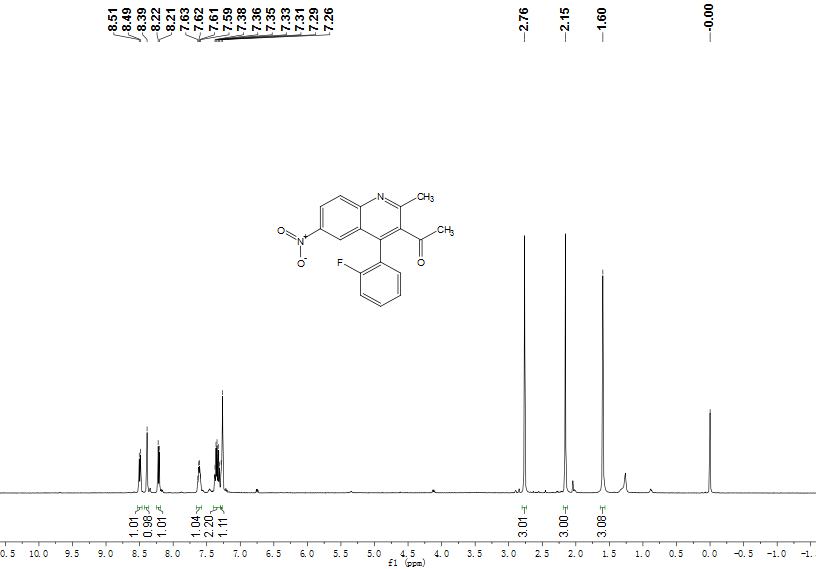
**

**1b**  ^13^C NMR (126 MHz, CDCl_3_)


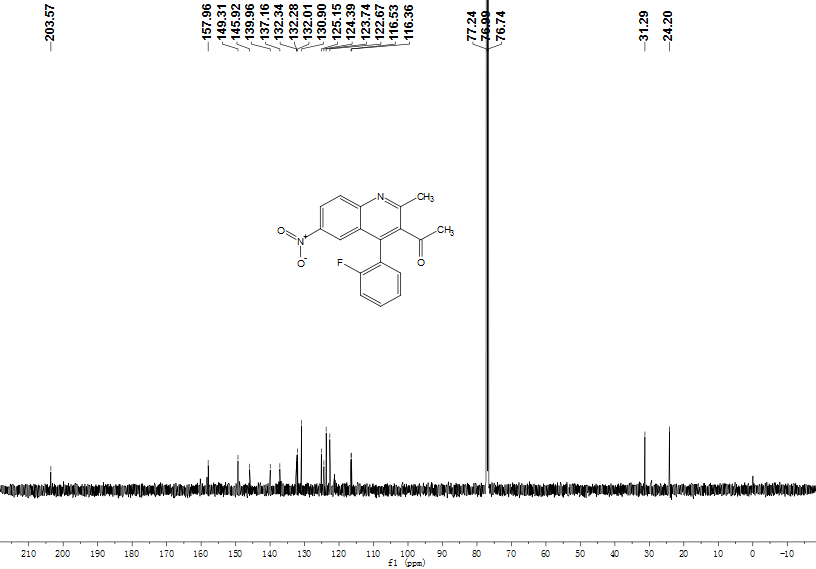


**1c**  ^1^H NMR (500 MHz, CDCl_3_)

**
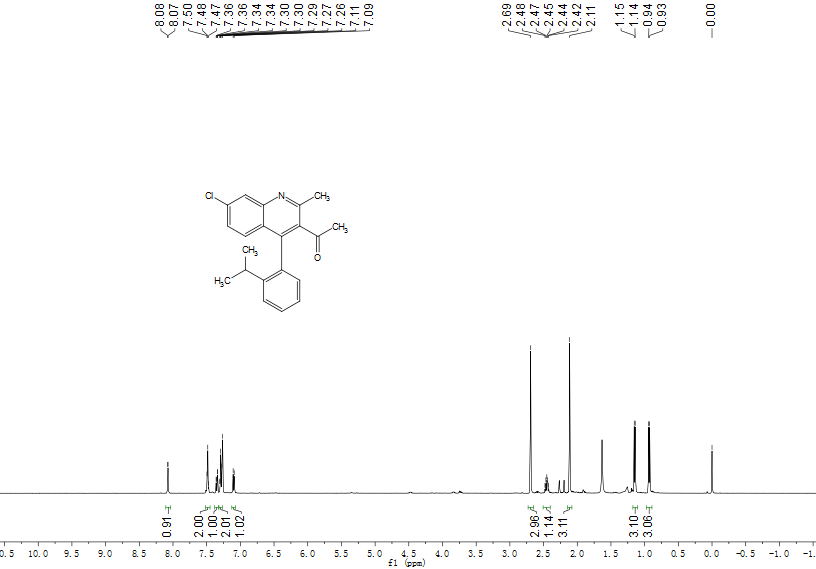
**

**1c**  ^13^C NMR (126 MHz, CDCl_3_)


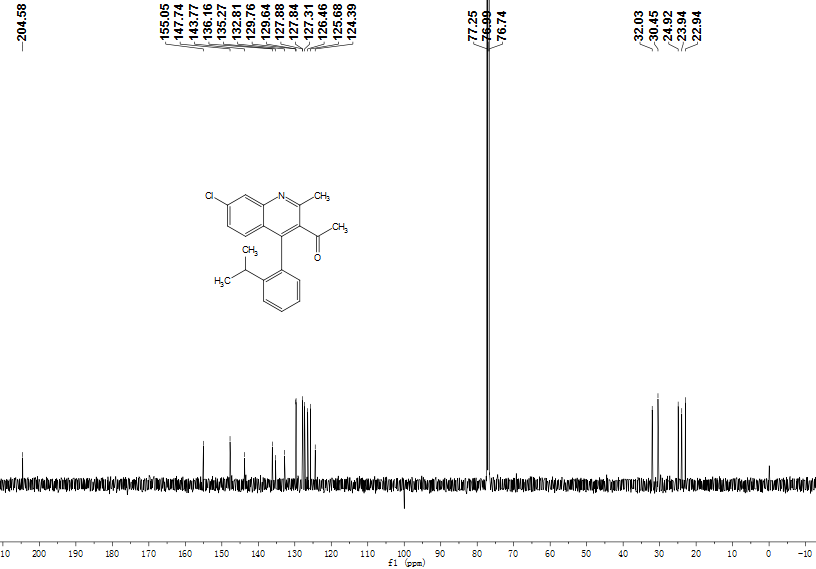


**1d**  ^1^H NMR (500 MHz, CDCl_3_)

**
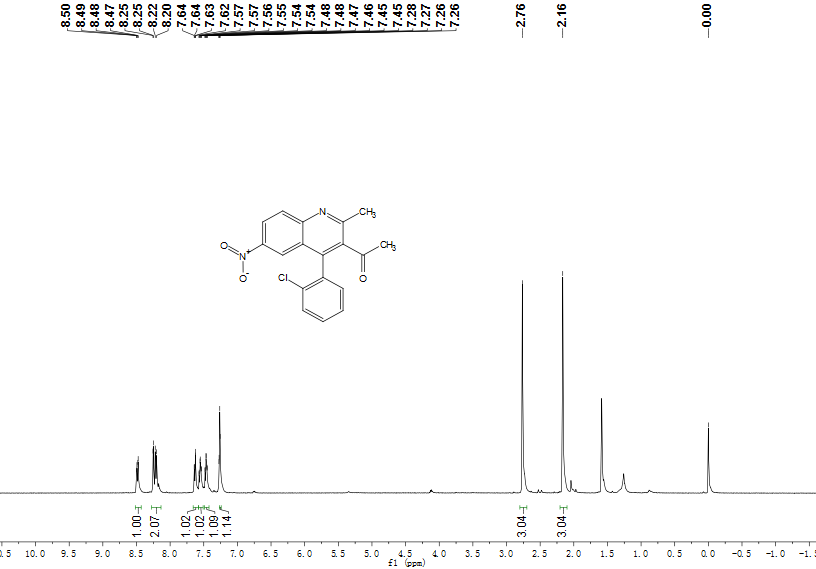
**

**1d**  ^13^C NMR (126 MHz, CDCl_3_)


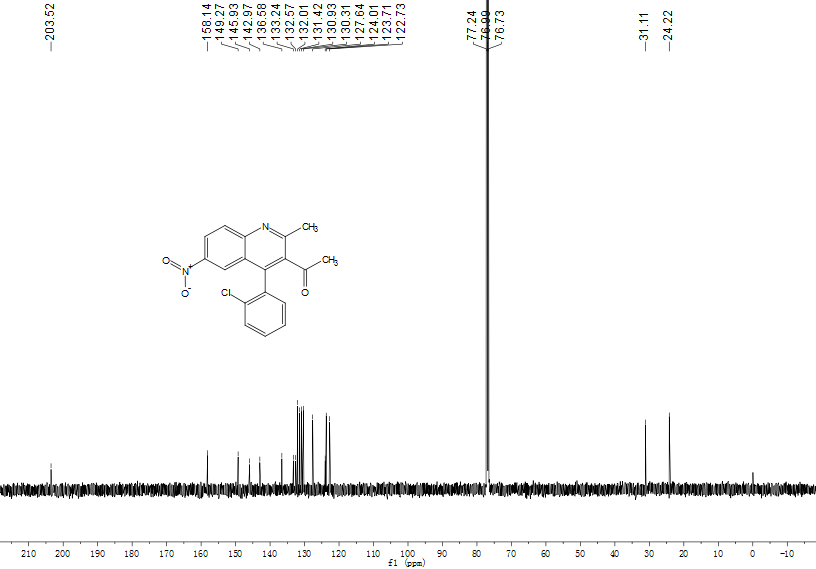


**1e**  ^1^H NMR (500 MHz, CDCl_3_)

**
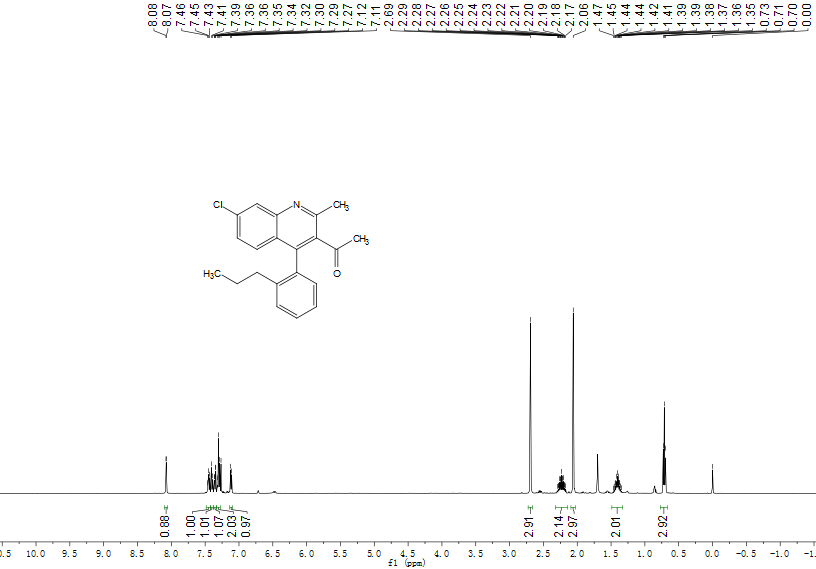
**

**1e**  ^13^C NMR (126 MHz, CDCl_3_)


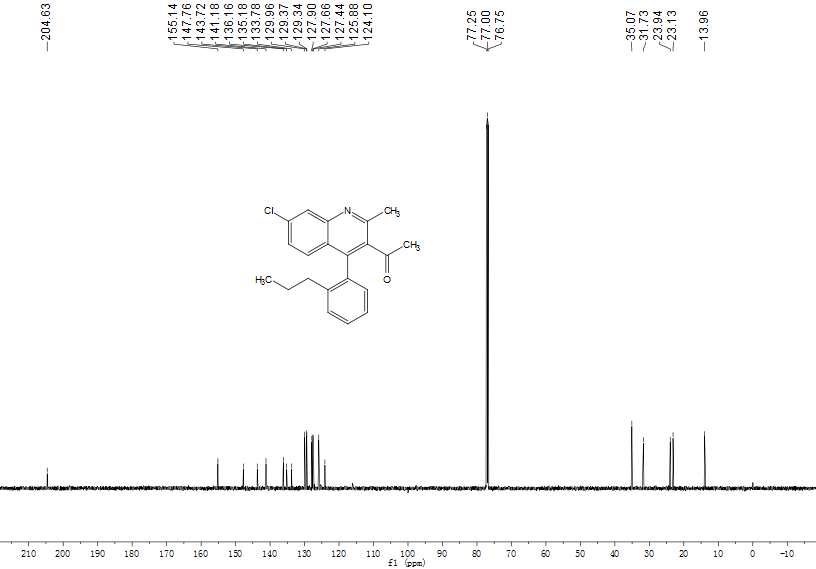


**1f**  ^1^H NMR (500 MHz, CDCl_3_)

**
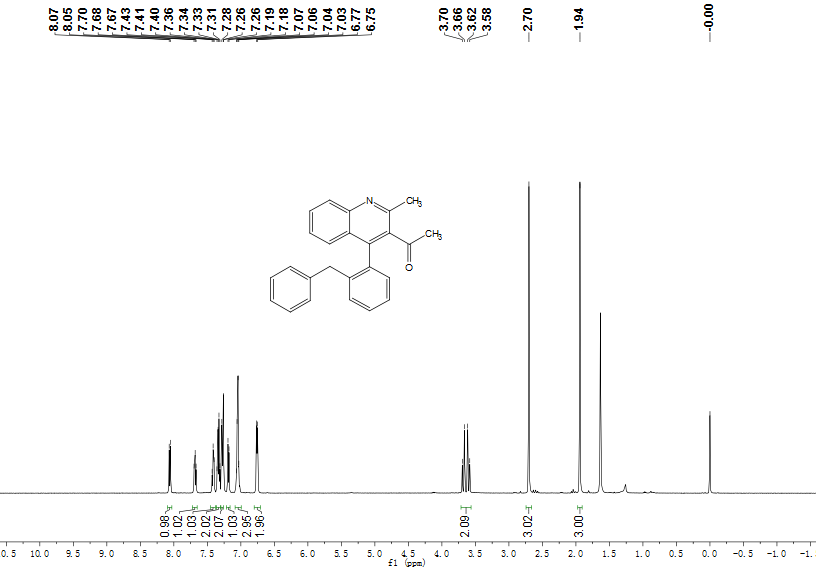
**

**1f**  ^13^C NMR (126 MHz, CDCl_3_)


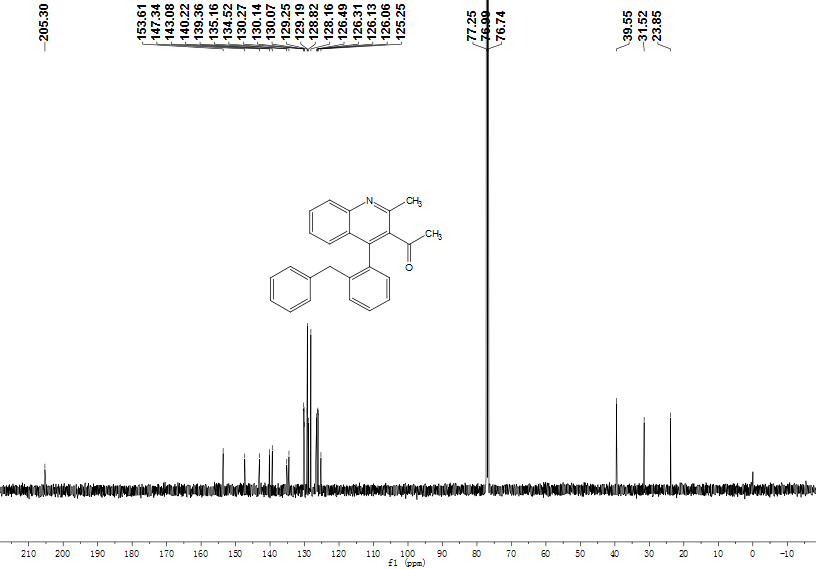


**1g**  ^1^H NMR (500 MHz, CDCl_3_)

**
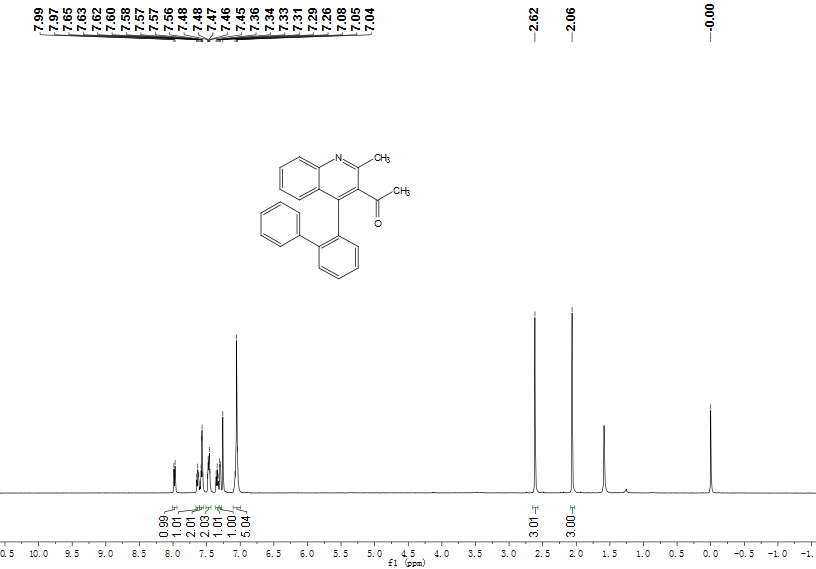
**

**1g**  ^13^C NMR (126 MHz, CDCl_3_)


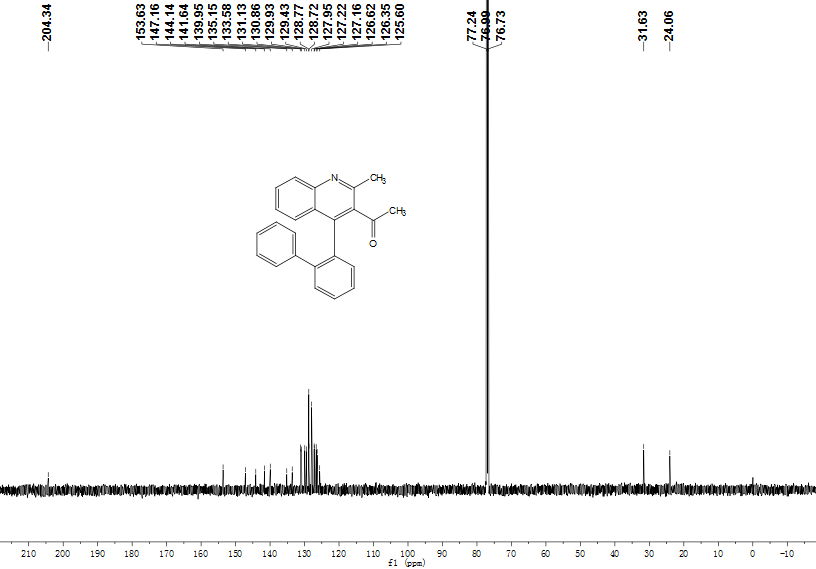


**1h**  ^1^H NMR (500 MHz, CDCl_3_)

**
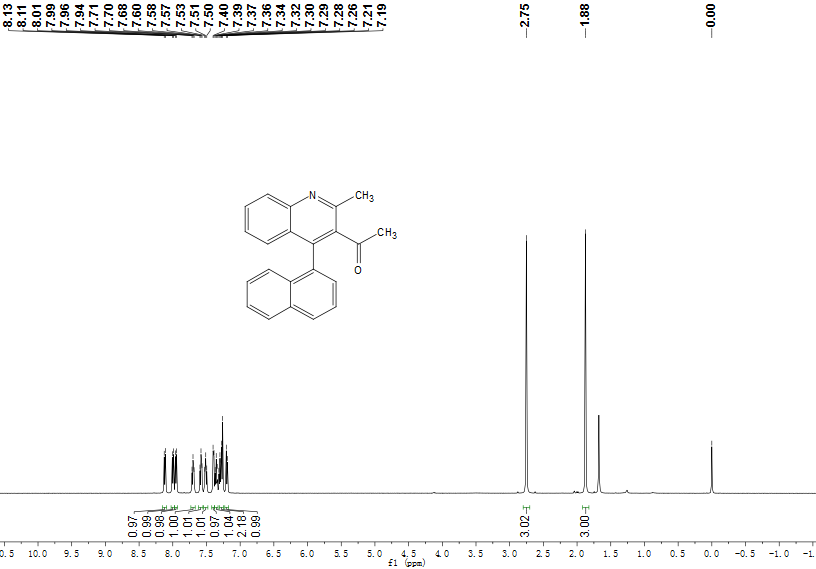
**

**1h**  ^13^C NMR (126 MHz, CDCl_3_)


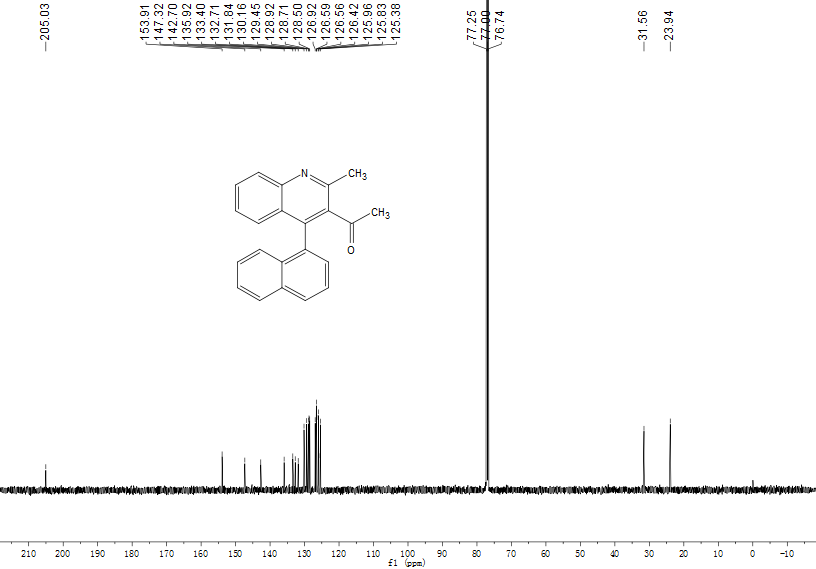


**1i**  ^1^H NMR (500 MHz, CDCl_3_)

**
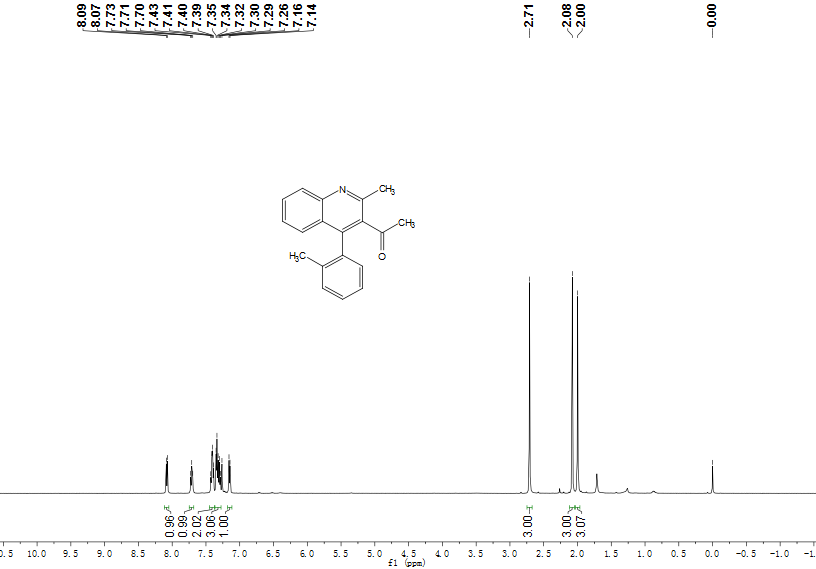
**

**1i**  ^13^C NMR (126 MHz, CDCl_3_)


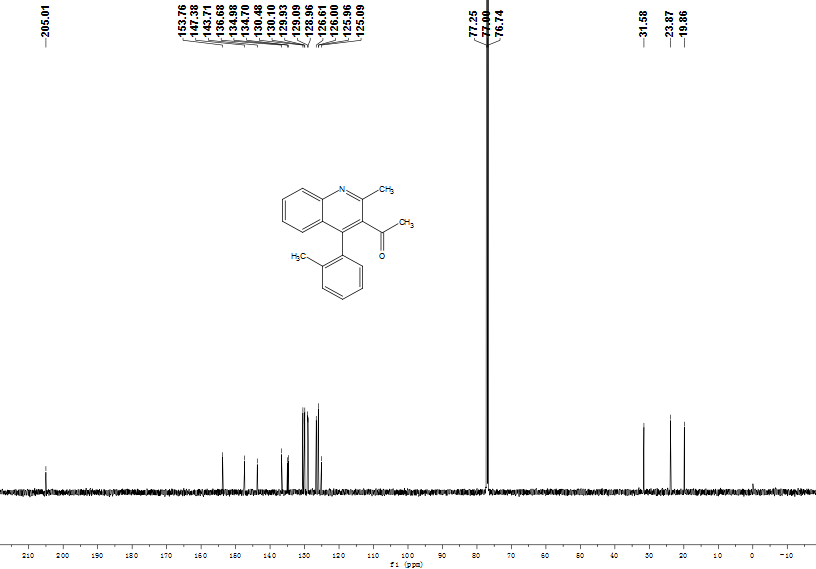


**1j**  ^1^H NMR (500 MHz, CDCl_3_)

**
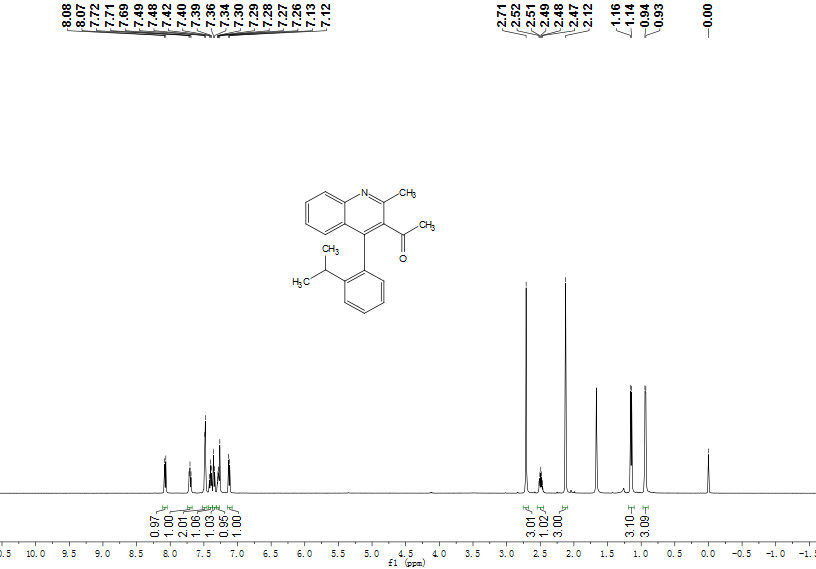
**

**1j**  ^13^C NMR (126 MHz, CDCl_3_)


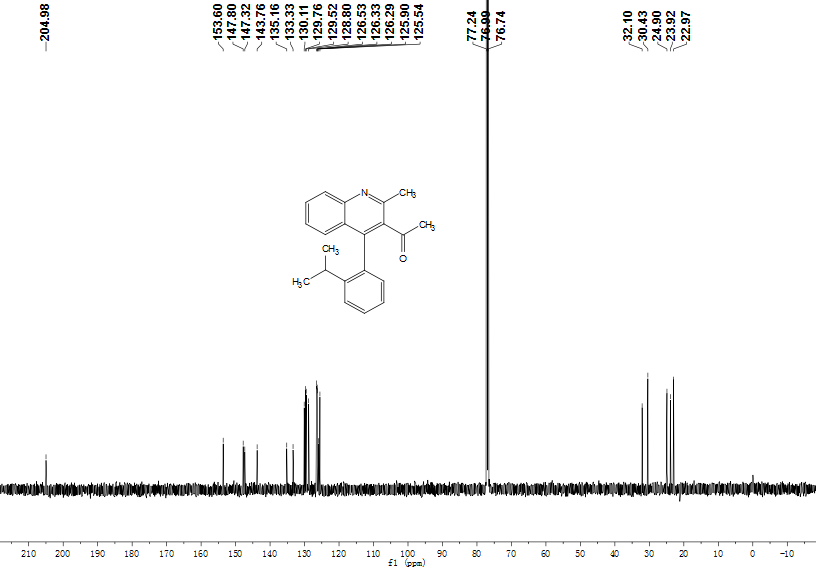


**1k**  ^1^H NMR (500 MHz, CDCl_3_)

**
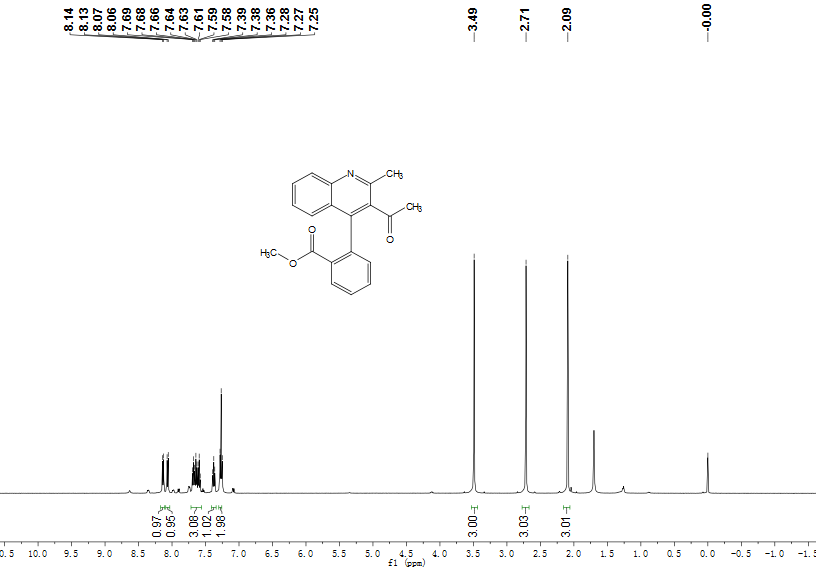
**

**1k**  ^13^C NMR (126 MHz, CDCl_3_)


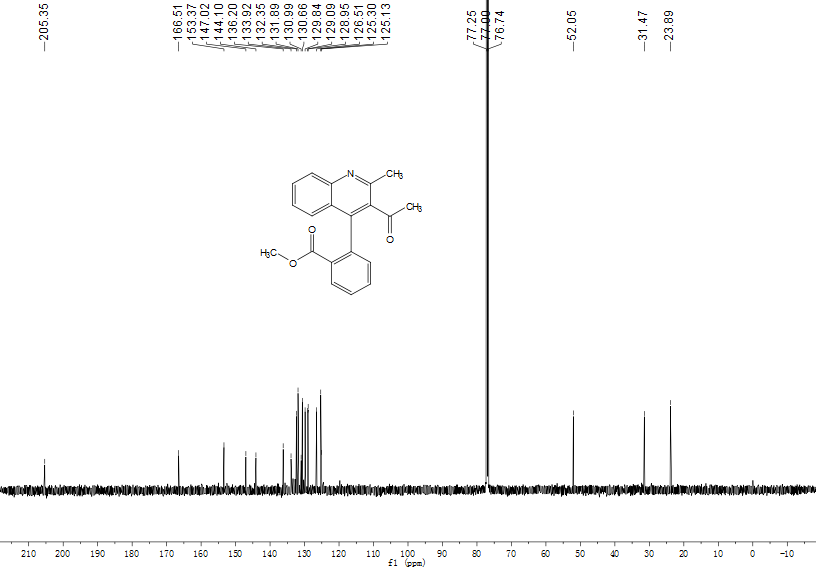


**1l**  ^1^H NMR (500 MHz, CDCl_3_)

**
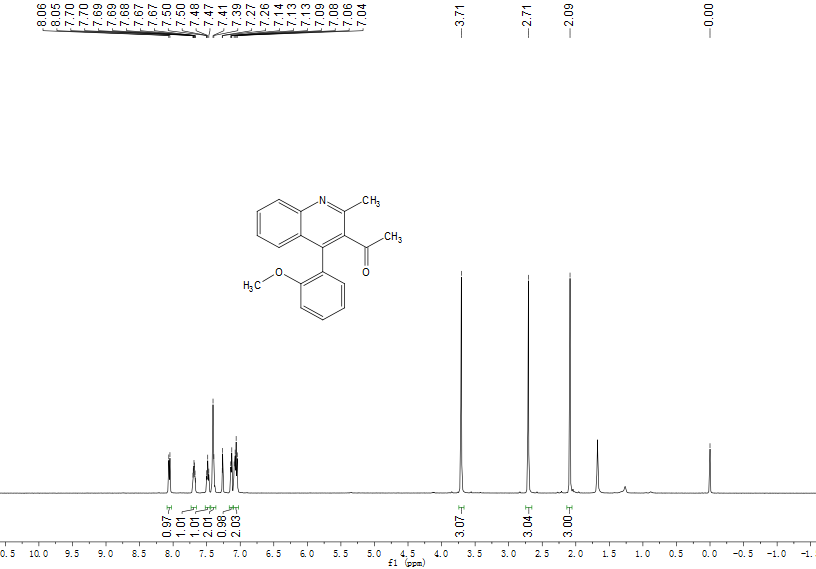
**

**1l**  ^13^C NMR (126 MHz, CDCl_3_)


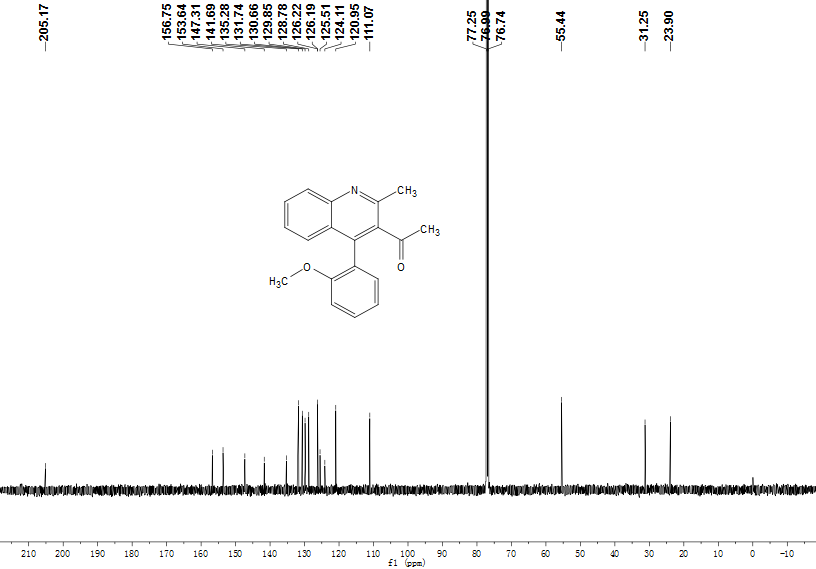


**1m**  ^1^H NMR (500 MHz, CDCl_3_)

**
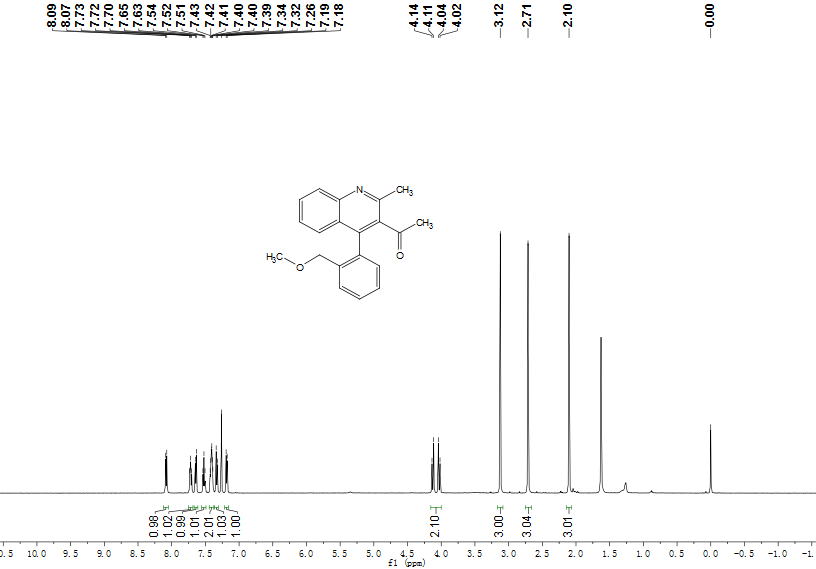
**

**1m**  ^13^C NMR (126 MHz, CDCl_3_)


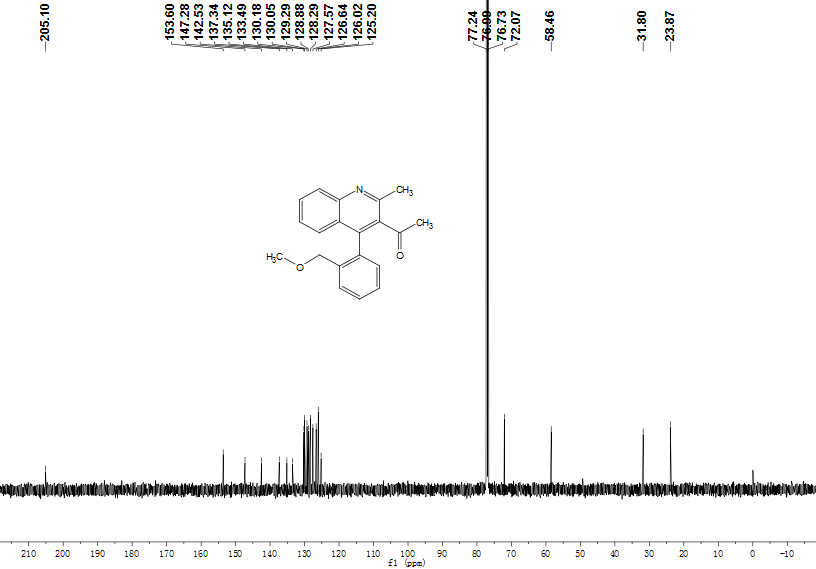


**1n**  ^1^H NMR (500 MHz, CDCl_3_)

**
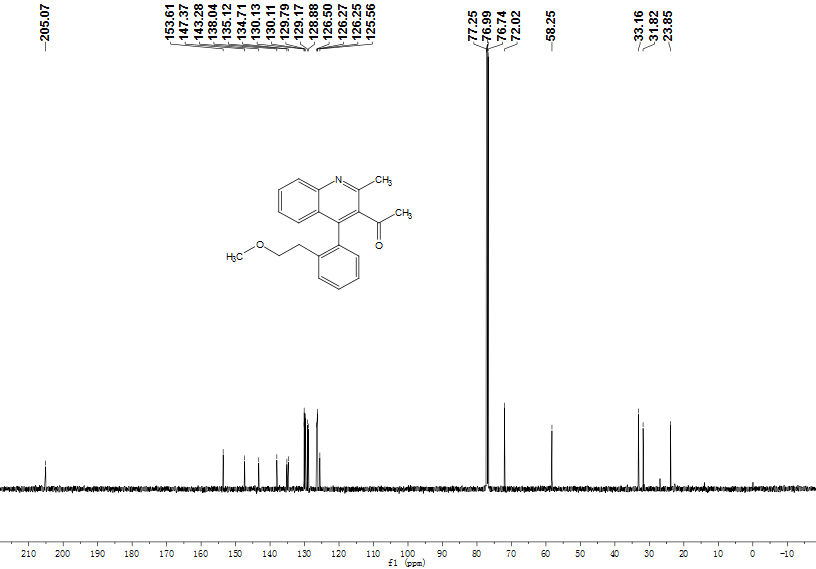
**

**1n**  ^13^C NMR (126 MHz, CDCl_3_)


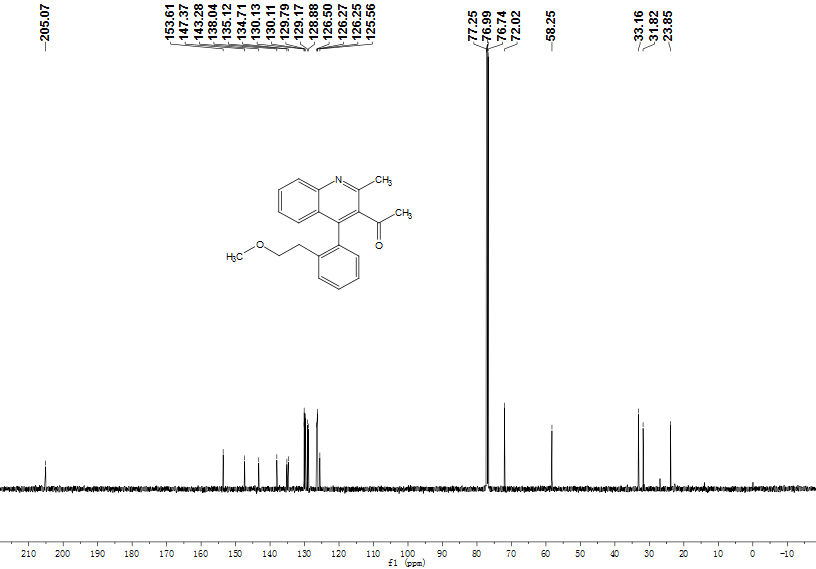


**1o**  ^1^H NMR (500 MHz, CDCl_3_)

**
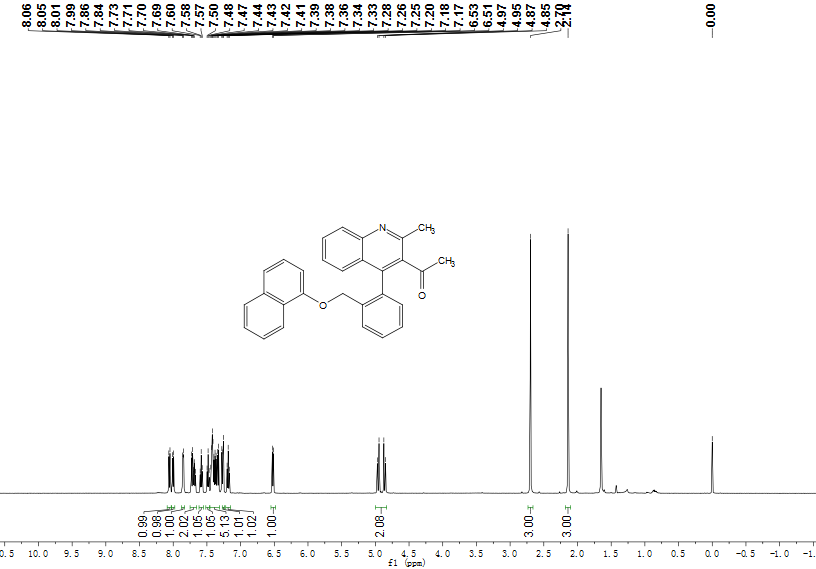
**

**1o**  ^13^C NMR (126 MHz, CDCl_3_)


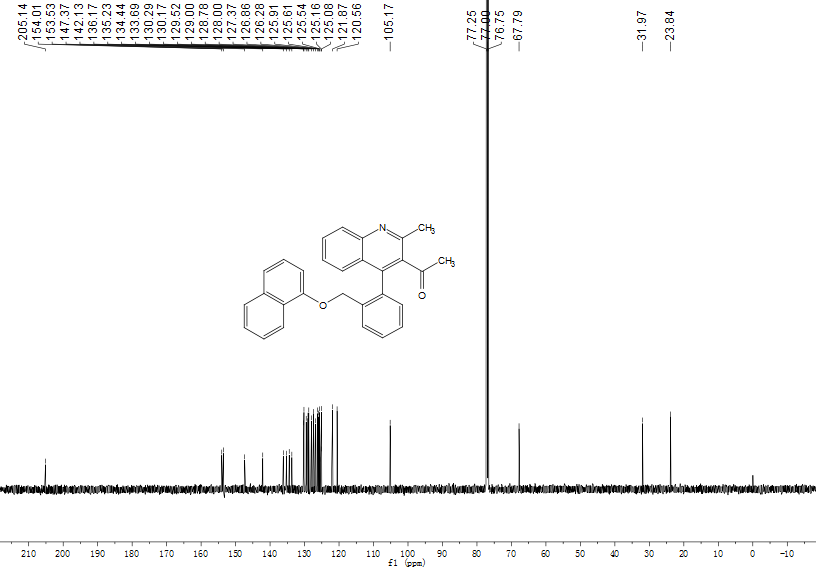


**1p**  ^1^H NMR (500 MHz, CDCl_3_)

**
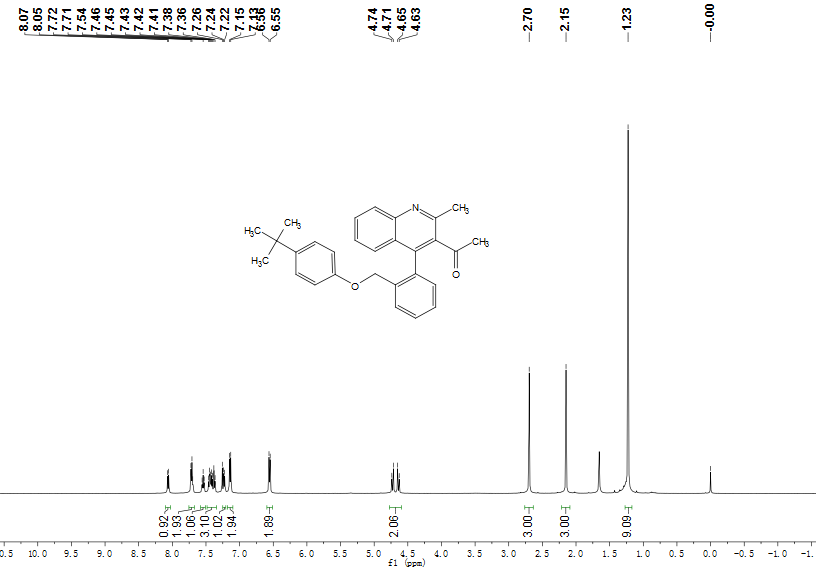
**

**1p**  ^13^C NMR (126 MHz, CDCl_3_)


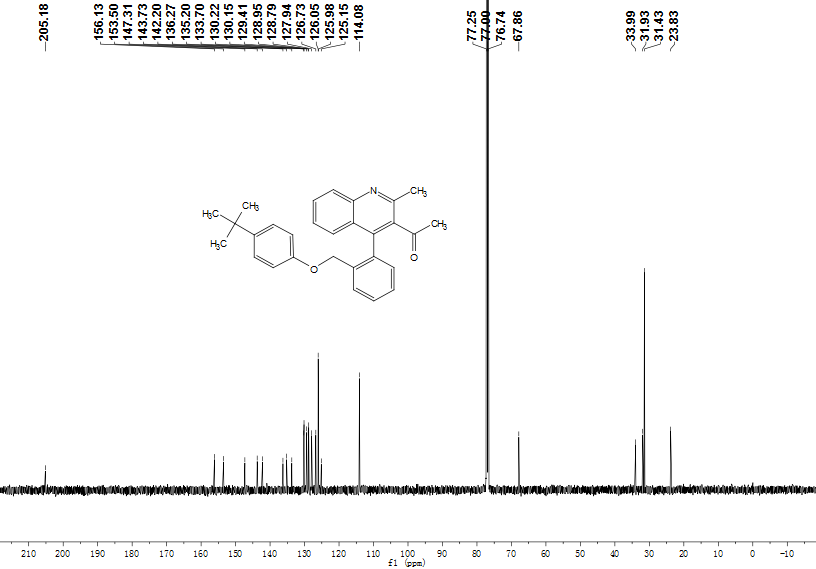


**1q**  ^1^H NMR (500 MHz, CDCl_3_)

**
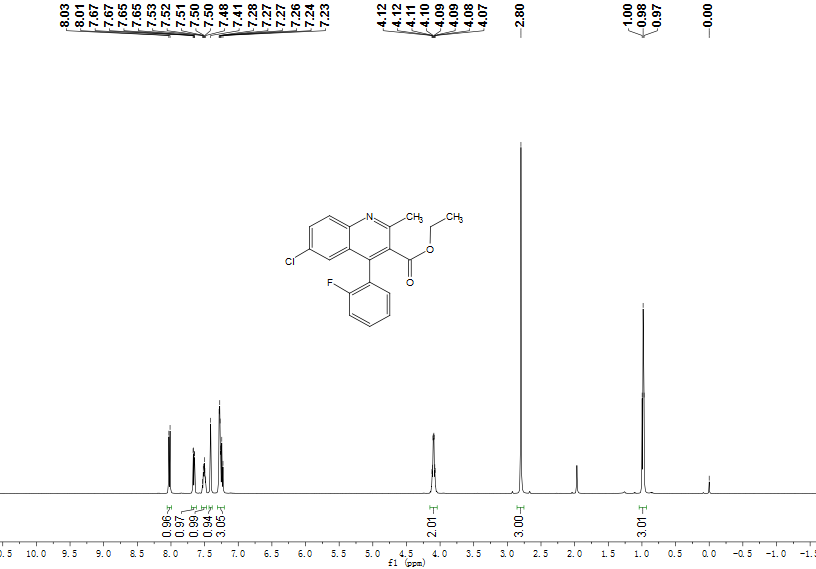
**

**1q**  ^13^C NMR (126 MHz, CDCl_3_)


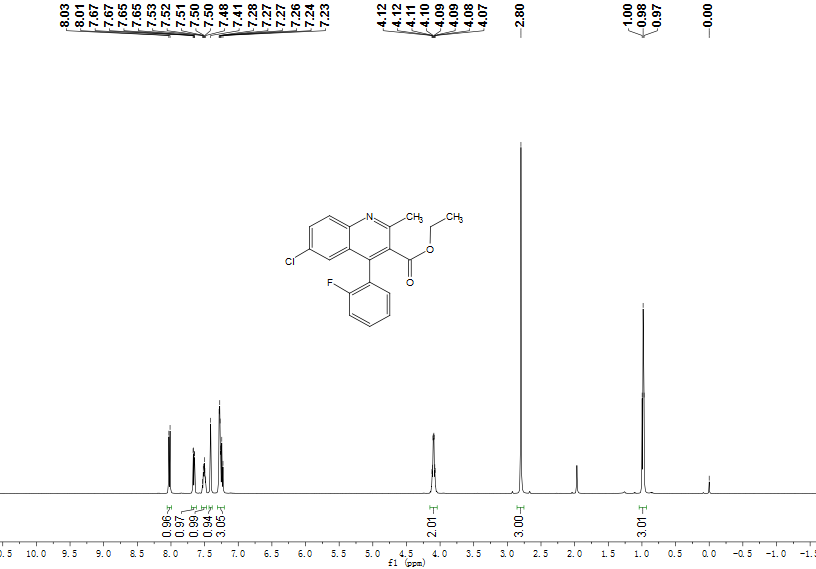


**1r**  ^1^H NMR (500 MHz, CDCl_3_)

**
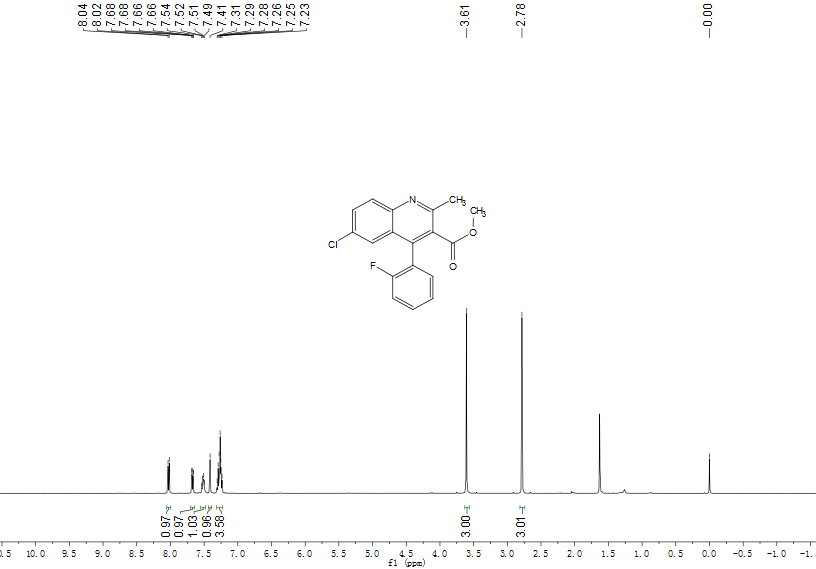
**

**1r**  ^13^C NMR (126 MHz, CDCl_3_)


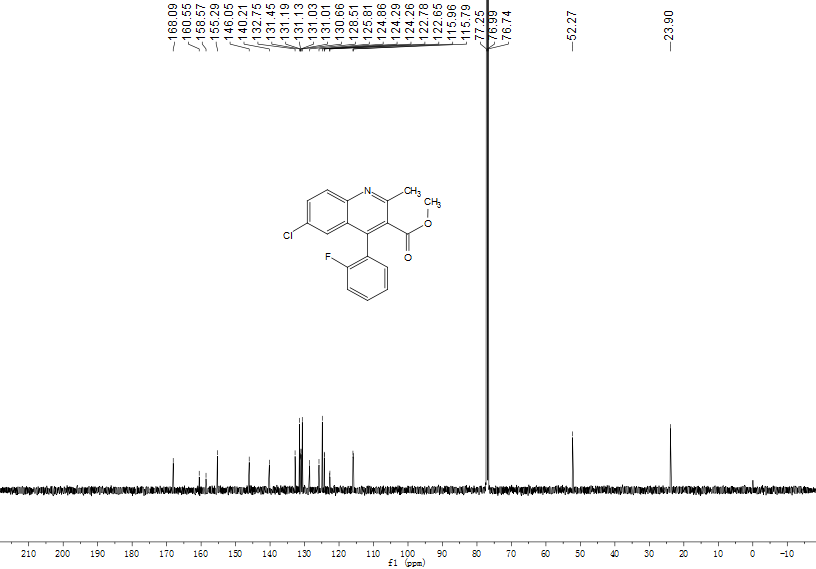


**1s**  ^1^H NMR (500 MHz, CDCl_3_)

**
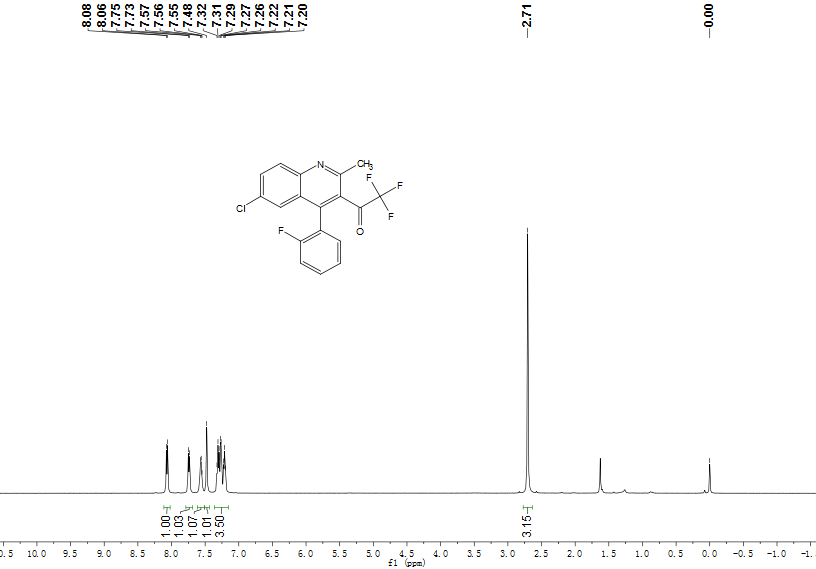
**

**1s**  ^13^C NMR (126 MHz, CDCl_3_)


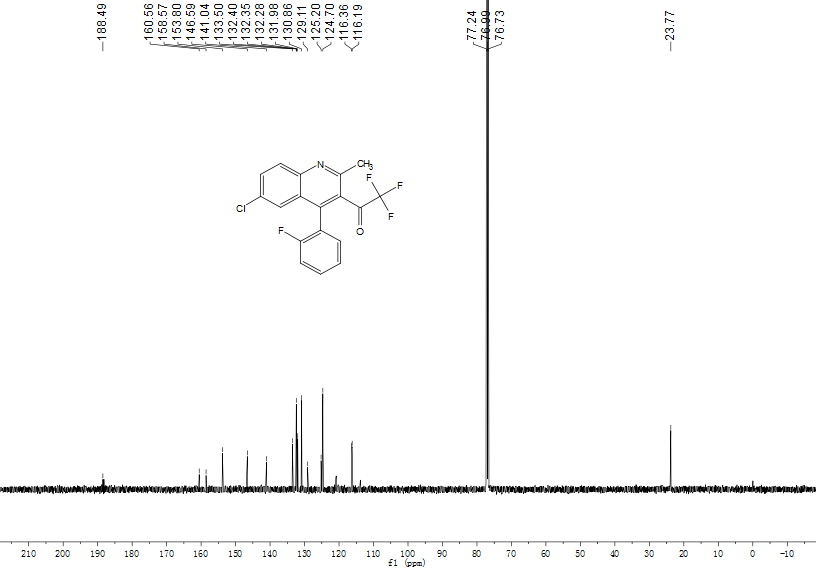

Supplement: Supplementary file 1 [file DataSheet1.docx]
